# Lysosome blockade induces divergent metabolic programs in macrophages and tumours for cancer immunotherapy

**DOI:** 10.1186/s13046-023-02768-0

**Published:** 2023-08-04

**Authors:** Jing Ma, Ruijuan Ma, Xueke Zeng, Liming Zhang, Jianing Liu, Wei Zhang, Tao Li, Hanjing Niu, Guochen Bao, Chaojie Wang, Peng George Wang, Jiajia Wang, Xia Li, Taotao Zou, Songqiang Xie

**Affiliations:** 1 School of Pharmacy, Institute of Chemical Biology, Henan Province Engineering Research Center of High Value Utilization to Natural Medical Resource in Yellow River Basin, State key Laboratory of Antiviral Drugs, School of Pharmacy, Henan University, Kaifeng, 475004 Henan China; 2 Joint National Laboratory for Antibody Drug Engineering, Henan University, Kaifeng, Henan, 475004 China; 3 Institute for Biomedical Materials and Devices (IBMD), Faculty of Science, University of Technology Sydney, Sydney, New South Wales Australia; 4 The Key Laboratory of Natural Medicine and Immuno-Engineering, Henan University, Kaifeng, 475004 China; 5 School of Medicine, The Southern University of Science and Technology, Shenzhen, 518005 Guangdong China; 6 School of Pharmaceutical Sciences Sun Yat, Sen University, Guangzhou, 510006 Guangdong China

## Abstract

**Background:**

Platinum-drugs based chemotherapy in clinic increases the potency of tumor cells to produce M2 macrophages, thus leading to poor anti-metastatic activity and immunosuppression. Lysosome metabolism is critical for cancer cell migration and invasion, but how it promotes antitumor immunity in tumours and macrophages is poorly understood and the underlying mechanisms are elusive. The present study aimed to explore a synergistic strategy to dismantle the immunosuppressive microenvironment of tumours and metallodrugs discovery by using the herent metabolic plasticity.

**Methods:**

Naphplatin was prepared by coordinating an active alkaline moiety to cisplatin, which can regulate the lysosomal functions. Colorectal carcinoma cells were selected to perform the in vivo biological assays. Blood, tumour and spleen tissues were collected and analyzed by flow cytometry to further explore the relationship between anti-tumour activity and immune cells. Transformations of bone marrow derived macrophage (BMDM) and M2-BMDM to the M1 phenotype was confirmed after treatment with naphplatin. The key mechanisms of lysosome-mediated mucolipin-1(Mcoln1) and mitogen-activated protein kinase (MAPK) activation in M2 macrophage polarization have been unveiled. RNA sequencing (RNA-seq) was used to further explore the key mechanism underlying high-mobility group box 1(HMGB1)-mediated Cathepsin L(CTSL)-lysosome function blockade.

**Results:**

We demonstrated that naphplatin induces divergent lysosomal metabolic programs and reprograms macrophages in tumor cells to terminate the vicious tumour-associated macrophages (TAMs)-MDSCs-Treg triangle. Mechanistically, macrophages treated with naphplatin cause lysosome metabolic activation by triggering Ca^2+^ release *via* Mcoln1, which induces the activation of p38 and nuclear factor-κB (NF-κB) and finally results in polarizing M2 macrophages. In contrast, HMGB1-mediated lysosome metabolic blockade in cancer cells is strongly linked to antitumor effects by promoting cytoplasmic translocation of HMGB1.

**Conclusions:**

This study reveals the crucial strategies of macrophage-based metallodrugs discovery that are able to treat both immunologically “hot” and “cold” cancers. Different from traditional platinum-based antitumour drugs by inhibition of DNAs, we also deliver a strong antitumour strategy by targeting lysosome to induce divergent metabolic programs in macrophages and tumours for cancer immunotherapy.

**Supplementary Information:**

The online version contains supplementary material available at 10.1186/s13046-023-02768-0.

## Background

Cancer immunotherapy is an effective anti-tumor strategy that has been developed in recent years and inhibits tumor metastasis by improving systematic immunity. However, the benefits of immunotherapy [1, 2] have largely bypassed the majority of patients with solid organ cancer, who are regarded as immunologically “cold”. Strategies that reinvigorate innate immune cells including tumour-associated macrophages (TAMs) are underrepresented within current immuno-oncology therapies [2]. Approximately half of the patients with cancer who receive chemotherapy are treated with clinically approved platinum drugs, such as cisplatin, carboplatin and oxaliplatin [3, 4]. Nevertheless, platinum-containing drug-based chemotherapy increases the probability of tumour cell lines inducing IL-10-producing M2 macrophages [3–6], which leads to poor antitumour metastasis activity and immunosuppression. By inducing caspase-3/GSDME-mediated secondary necrosis, platinum-based drug also exerts their cytotoxicity on macrophages, further limiting their antitumour efficiency [7]. Therefore, studies on novel chemotherapeutic candidates that can achieve antitumor efficacy *via* reinvigorating TAMs instead of depleting them [8–10] are crucial.

Macrophages [11–14] are a type of specialized phagocyte with a strong ability to phagocytose extracellular substances and effectively degrade them in lysosomes [10, 15, 16]. Recent studies indicate that interacting with subcellular compartments, including lysosomes, instead of nuclei [17] can effectively improve the efficacy of platinum-based drugs [18–24]. Therefore, regulating lysosomal metabolic states is a major challenge and a potential breakthrough in the development of anti-TAM therapies and chemotherapeutic drugs. To date, antineoplastic protocols, reported by our group [24–30] and others [3, 4], have failed to simultaneously trigger divergent lysosomal metabolic characteristics in tumour cells and macrophages to prevent chemotherapy immunosuppression [28–30]. In particular, several attributes of lysosomes as metabolic regulators suggest a specific role in cancer immunotherapy [31–34], but how it promotes antitumor immunity in tumours and macrophages is poorly understood and the underlying synergistic mechanisms are elusive.

**Fig. 1:**
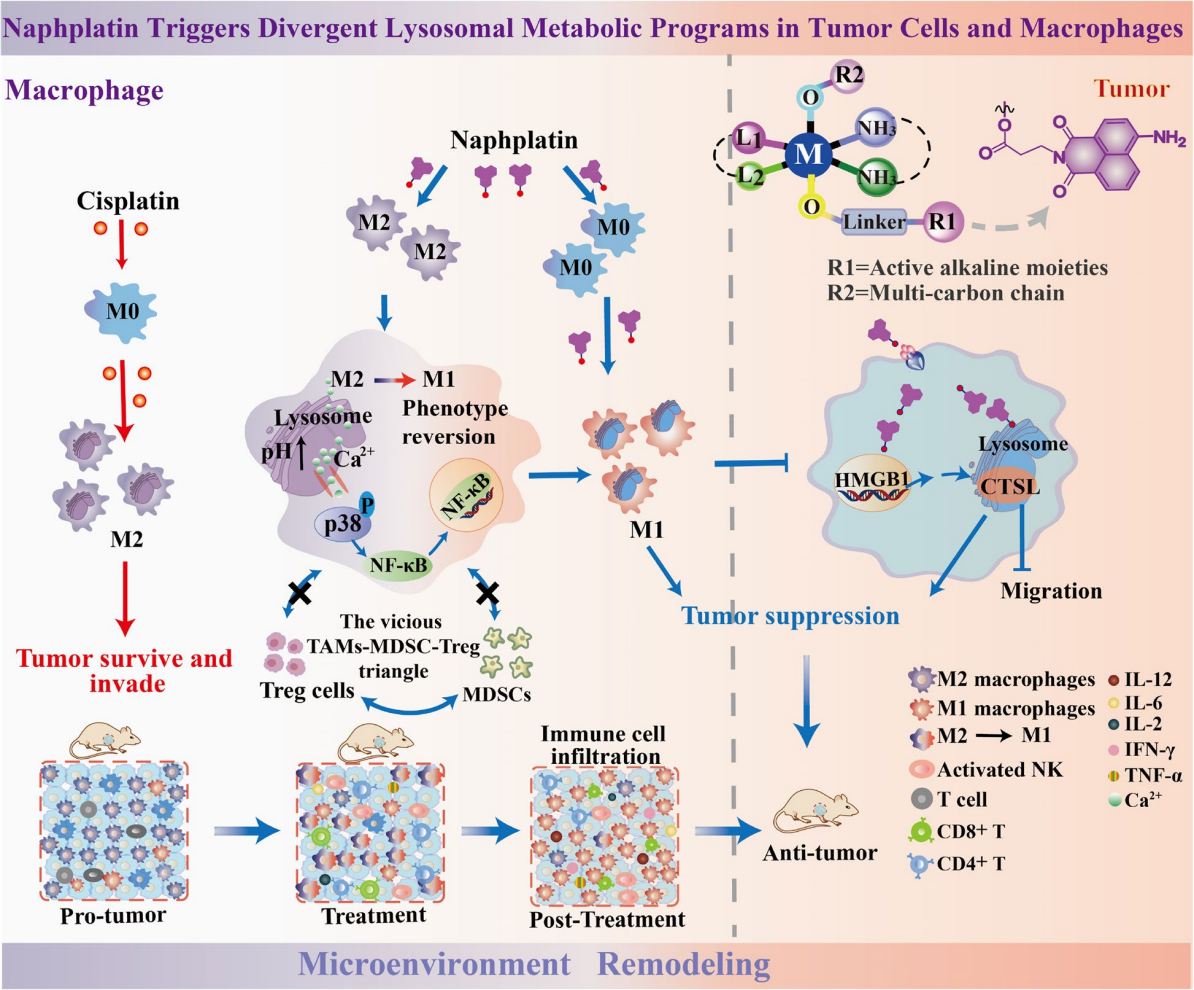
The schematic illustration of antitumor mechanisms of naphplatin. The active alkaline moieties are designed to explore the inherent lysosomal metabolic plasticity of cells. The scheme showing naphplatin triggers divergent lysosomal metabolic programs in tumour cells and macrophages to deter chemotherapy immune evasion. Naphplatin could regulate HMGB1-mediated lysosomal function of tumour cells and macrophages for remodeling the tumour microenvironment. In contrast, cisplatin decreased the macrophages in the spleen and increased the M2 macrophages to induce severe tumour immunosuppression

Here, we demonstrated a new synergistic effect for cancer immunotherapy by activation of lysosomal functions in macrophages, and inhibition in colorectal cancers, which leads to termination of the vicious TAMs-myeloid derived suppressor cells (MDSCs)-regulatory T cell (Treg) triangle (Fig. 1). By exploring a novel naphthalimide-platinum drug (named naphplatin) as an immune modulator, we found that in contrast to cisplatin, naphplatin-induced antitumor immunogenic cell death (ICD) is macrophage dependent which is closely connected with naphplatin’s antitumour and anti-metastasis efficacy. Notably, naphplatin localizes in lysosome of both BMDM and M2-BMDM to trigger Ca^2+^ release *via* Mcoln1, inducing the activation of MAPK p38 and NF-κB and induction of M1 macrophages. We also discovered that HMGB1-mediated CTSL and autophagy-lysosome pathway blockade in cancer cells plays vital roles in the antitumor effects by inhibiting the formation of the Beclin-1-PI3K-III complex.

## Materials and methods

### Cell lines and reagents

Murine macrophage cell line Raw264.7 was isolated from marrow. Bone marrow-derived macrophage (BMDM) was cultured with high glucose-Dulbecco’s modified Eagle’s medium (Corning) supplemented with 10% feral bovine serum (FBS) (Gibco, Australia) at 37°C with 5% CO_2_. Purified anti-mouse Ly-6G/Ly-6C (Gr-1) antibody (RB6-8C5) and rat IgG2bκ isotype control, Anti-CD3 (145-2C11), anti-CD8 (53-6.7), anti-CD25 (PC-61.5.3) mouse neutralizing antibody, and isotype control (N/A) were purchased from BioLegend. The macrophage depletion reagent, clodronate liposomes(Clo) and CsA were purchased from FormuMax Scientific USA.

### In vitro inhibitory effect on cell proliferation

Cell lines from colorectal cancer (HT-29, HCT-116 and CT-26), hepatic cancer (HepG-2 and Huh-7), breast cancer (MDA-MB-231 and MCF-7) and lung cancer A549 (5000/well) were incubated in 96-well plates at 37 °C with 5% CO_2_ cultured with 100 µL high glucose-Dulbecco’s modified Eagle’s medium (Corning) or RPMI supplemented with 10% feral bovine serum (FBS) (Gibco, Australia) for 24 h. Drugs dissolved at different concentrations in freshly prepared culture medium (100 µL) were incubated for another 48 h. Then MTT (5 mg/mL, 20 µL) was added and incubated for another 3 h. We next removed the medium and added 150 µL DMSO to dissolve the purple crystal. By a Bio-Rad 680 microplate reader, the absorbance was measured at 570 nm. Based on three parallel experiments, the IC_50_ values were counted using GraphPad Prism software.

### Transwell migration assay

Transwell migration assays were performed *via* a 8 μm pore using Boyden’s chamber in 24-well culture plate. Cells were seeded onto transwell inserts and incubated with naphplatin or cisplatin at 37 ºC in 5%CO_2_ for 24h. Cells were fixed with 4% paraformaldehyde for 20 minutes and stained with crystal violet for 5 minutes. Cells that did not migrate across the transwell membrane were then removed by gently wiping with a cotton swab. Migrated cells were record images with Leika inverted microscope.

### Annexin V-FITC/propidium iodide staining

We evaluated apoptosis in CT-26 and HCT-116 cells by Annexin V-FITC/PI(BD Biosciences) staining and flow cytometry. After incubated cells (1 × 10^5^ cells/well) in six-well plates for 24 h, naphplatin (1 or 2 μM) or cisplatin (2 μM) were added and hatched for another 24 h. The cells were centrifuged at 3000 r/min for 5 min, then the supernatant was discarded and the pellet was resuspended in 1 × binding buffer at a density of 1.0 × 10^5^-1.0 × l0^6^ cells per mL. One hundred μL of the sample solution was transferred to a 5 mL culture tube, and incubated with 5 μL of FITC-conjugated annexin V and 5 μL of PI for 15 min at room temperature in the dark. Four hundred μL of 1×binding buffer was added to each sample tube, and the samples were analyzed by flow cytometry.

### Western blot assay

Targeted proteins including p53, p62, LC3II/I, Phospho-histone H2A-X (Ser139), HMGB1, nuclear-HMGB1, cytosolic-HMGB1, ERK, p38, p65, Arg1, iNOS and β-catenin were detected by western blot analysis. CT-26 and HCT-116 cells were seeded in 6-well plates and incubated for 24 h. Naphplatin at 5, 10, and 15 μM was added and incubated for another 24 h. After being harvested and centrifuged, the cells were washed with PBS for three times. Using a BCA assay kit (Beyotime, China) to determine the total protein, the total lysates were denatured in 5 × SDS-loading buffer at 100 °C for 10 min, the total proteins were separated by 12% SDS-PAGE for another 2 h and transferred onto PVDF membranes, which were blocked with 5% milk in Tris-buffered saline with Tween® 20 detergent (TBST) and then incubated with primary antibodies at 4 °C overnight followed by appropriate secondary antibodies at room temperature for 1 h. Immunoreactive protein bands were visualized with an enhanced chemiluminescence system (Amersham™ Imager 600; GE Healthcare Bio-Sciences Corp., Piscataway, NJ, USA) and an Odyssey® M Infrared Imaging System ( LI-COR Biosciences, Lincoln, NE, USA).

### Real-time qPCR

Total RNA (1 μg) was extracted from cells or tumour tissues by using the TRIzol reagent (Invitrogen, USA) and reverse-transcribed into cDNA with ReverTra Ace Kit (Toyobo). The THUNDERBIRD SYBR qPCR Mix (Toyobo) on a StepOnePlus Real-Time PCR System (Thermo Fisher Scientific) was used to amplify the cDNA. The mRNA levels were normalized by β-actin. The primer sequences are shown at Supplementary Table.

### Generation of mouse BMDM and M2-BMDM

The bone marrow cells were isolated from female C57BL/6 mice femurs and cultured with 20 ng mL^−1^ recombinant M-CSF (PeproTech) for 5 days. On day 6, naive macrophages (BMDMs) were collected and then the BMDM-M2 or BMDM-M1 macrophages were generated by stimulating with 20 ng mL^−1^ IL-4 (PeproTech) or 100 ng mL^−1^ LPS (Sigma-Aldrich) plus 20 ng mL^−1^ IFN-γ (PeproTech), respectively.

### Tumour-infiltrating lymphocyte and macrophage isolation

Tumour tissues were collected and cut into small pieces in PBS, enzyme digestion was performed with 2 units mL^−1^ hyaluronidase (Sigma-Aldrich), 1 mg mL^−1^ collagenase (Sigma-Aldrich) and 0.1 mg mL^−1^ DNase (Sigma-Aldrich) for 1 h after centrifugation. The cell suspension was centrifuged with Ficoll to get the mononuclear cells and/or sorted with anti-F4/80 microbeads (Miltenyi Biotec) to get tumor-infiltrating macrophages. For tumor-infiltrating lymphocyte and macrophage isolation in tumor ascites, ascites volume and the CD45− cells were counted, ascites were centrifuged with Ficoll directly to get mononuclear cells. Then sorted with anti-F4/80 microbeads (Miltenyi Biotec) to get tumor-infiltrating macrophages.

### Lysosomal pH value assay

After trypsinzing and collecting the cells, the lysosomal pH value of BMDM-M1, BMDM-M2, and naphplatin-treated BMDM-M2 cells was measured by staining with LysoSensor Yellow/Blue DND-160 (1:1,000 diluted and pre-incubated in complete culture medium for 30 min at 37 ℃. Thermo Fisher Scientific, L7545) for 3 min at 37 ℃. Cells were transferred into a black 96-well plate (1 × 10^6^ cells per 200 μl per well) after being washed with PBS. The fluorescence intensity was measured on the Synergy H1 (BioTek) at Ex-360/Em-440 and Ex-360/Em-550 with 10 μM of valinomycin and 10 μM of nigericin was added. We used the intracellular pH Calibration Buffer Kit (Thermo Fisher Scientific, P35379) to measure the standard curve of lysosomal pH value. For LysoSensor Green probes (Thermo Fisher Scientific, L7535) staining in BMDM-M1 and M2 cells, cells were incubated with probes for 30 min to 1 hour, then the PBS was used to collect and wash the cells to measure the mean fluorescence intensity by flow cytometry.

### Intracellular calcium concentration assay

Cells were incubated with 5 μM Fluo-4 AM (Thermo Fisher Scientific, F14201) in PBS. We determined the calcium concentration by the Fluo-4 AM mean fluorescence intensity with ionomycin stimulated (Cayman, 10004974) on a real-time live-cell imaging with a high-speed laser confocal microscope.

### ICP-MS determination of cellular accumulation and binding to nuclear DNA

By using MDA-MB-231, MCF-7, HT-29, HCT-116, CT-26, HepG-2, and A549 cells, cells (2× 10^5^ cells/well) was seeded in 6-well plates and incubated for 24 h. After incubated cells with 10 µM naphplatin or cisplatin for 8 h, the cells were washed with PBS for three times and then harvested by trypsinization. To determine the intracellular distribution of platinum, DNA, endoplasmic reticulum(ER), lysosomes and mitochondria were isolated by corresponding preparation kits, which were then digested with nitric acid and detected by ICP-MS.

### Immunofluorescence and immunohistochemical staining

For immunofluoresence staining, cells were seeded in confocal dish with or without naphplatin and CsA pretreatment for 24 h. The cells were fixed with 4% paraformaldehyde in PBS pH 7.4 for 10 min at room temperature and then permeabilizated with 0.5% Triton X-100 in PBS for 10 min. After being blocked with 2% BSA in PBS containing 0.1% Tween-20 for 30 min, cells were incubated overnight at 4 °C with Lamp1 (Abcam, ab25245, 1: 500), and Lamp2 (Abcam, ab25339, 1: 100), p65 (Cell Signaling Technology, #8242, 1: 400), in 2% BSA in PBS containing 0.1% Tween-20. After washing and incubating with secondary antibody for 1 h at room temperature, the DAPI (4',6-diamidino-2-phenylindole) (2 μg mL^−1^) was used to stain nucleus, which was then used for immunofluoresence analysis through OLYMPUS two-photon microscope. For immunohistochemical staining, melanoma tissues were isolated from tumour-bearing mice and fixed in 37% formalin and embedded in paraffin. Next, the Ultra Vision Quanto Detection System kit (Thermo Scientific) was used to incubate the section with the anti-Granzyme B (Abcam, ab4059, 1: 1,000) and observed by using the DAB Quanto kit (Thermo Scientific).

### Immunoprecipitation analysis

Cells were lysed in RIPA buffer (Millipore) at 4°C. Samples were pre-cleared with protein sepharose (Millipore) containing equal amounts of proteins and subsequently statistical analysis. Next specific antibodies or irrelevant immunoglobulin in the presence of protein sepharose beads were incubated. The beads were washed with RIPA buffer for three times, and the immune complexes were eluted from the beads and subjected to SDS-PAGE and western blot analysis.

### In vivo antitumor and anti-metastasis assays

For the in vivo antitumor assay, CT-26 cells (3.0 × 10^5^-3.5 × 10^5^ cells per mouse) were injected subcutaneously and then inoculated for 7 days. Then the drugs dissolved in the glucose solution in each group were injected intra-peritoneally once every 2 days for a total of four treatments (*n* = 8 mice per group). Finally, the mice were euthanized and the tumour tissues were weighed to calculate the inhibition rate as follows. Inhibitory rate (%) = [(the average tumour weight of the control group − the average tumour weight of the drug-treated or positive group)/ the average tumour weight of the control group× 100%]. To further explore the relationship between anti-tumour activity and immune cells, the blood, tumour and spleen tissues were collected and analyzed by flow cytometry to determine macrophages, M1/M2-macrophages, CD45^+^, CD19^+^ B cells, CD5^+^ T cells, CD4^+^ T cells, CD8^+^ T cells, Treg, dendritic cells, mMDSCs, and gMDSCs.

For in vivo anti-metastasis assay, CT-26 cells (3.0-3.5 × 10^5^ cells per mouse) were injected via the tail vein and also inoculated for 7 days. The mice were euthanized, the lung metastasis nodules were fixed with 4% paraformaldehyde and counted to calculate the inhibition rate as follows. Inhibitory rate (%) = [(the average nodules of the control group − the average nodules of the drug-treated or positive group group)/the average nodules of the control group × 100 %].

### In vivo macrophage-deficient antitumor assay

For in vivo macrophage-deficient antitumor assay, CT-26 cells (3.0-3.5 × 10^5^ cells per mouse) were injected subcutaneously and then inoculated for 4 days. clodronate liposomes (Clo, 200 μL) were injected intra-peritoneally once every 4 days for a total of four treatments (n = 8 mice per group). After another 4 days, the drugs was injected intra-peritoneally once every 2 days for a total of four treatments and then the mice were euthanized to calculate the inhibition rate. Finally, in order to further probe the interaction between anti-tumour effects and macrophages, the tumour, blood and spleen samples were analyzed by flow cytometry to explore the changes in the proportion of immune cells.

## Results

### Synthesis and characterization

We prepared naphplatin by coordinating the classical core of amonafide or mitonafide to cisplatin (Fig. 1), which are common active alkaline moieties that can regulate lysosomal functions [29–32]. For comparison, we synthesized cisplatin-based compounds (**14a**), the oxaliplatin-based scaffolds (**7a-7c** and **12a-12d**), and the compound **13** without naphthalimide modification (Fig. S1 and Scheme S1). The preparations of **7a-7c**, **12a-12d**, **14a**, and naphplatin are summarized in Schemes S1-S6. Naphthalimide intermediates **5a-5c** and (Scheme S6) were prepared as previously reported by our group [28–30] and their structures were also fully characterized (Figs. S2–S42). The purity of final platinum complexes was confirmed to be ≥ 95% by analytical HPLC (Table S1 and S2). Determination of the apparent oil–water partition coefficient (log P_app_ = 2.71) (Table S3) predicted its good solubility in cancers. Considering the possibility of many reducing agents in cells, acceptable stability was tested and confirmed in water and RPMI 1640 solution to be within 72 hours (Fig. S43).

### Naphplatin exhibits excellent activities of antitumor and antimetastasis activity in colorectal cancer in vitro and in vivo

We first evaluated the cytotoxicity against various cancer cell lines by MTT assay in vitro (Table 1, S4 and S5). Naphplatin exhibited significantly high antitumor efficacy among all the drugs on the multiple cancer cell lines, leading to over 200-fold lower IC_50_ values than commercial cisplatin and oxaliplatin. Notably, naphplatin exhibited better antitumour activities than Pt(IV) drug **13**, indicating the essential role of naphthalimide ligand in the antitumor potency. Drug uptake in cancer cells was determined using inductively coupled plasma-mass spectrometry (ICP-MS, Fig. S44a). The selectivity index (SI) values of naphplatin (SI = 4.83, Table S6) were much higher than those of cisplatin (SI = 0.24) and oxaliplatin (SI = 0.58).

**Table 1:** IC_50_ values (concentrations of 50% inhibition of cell proliferation) (µM) of naphplatin, the Pt(IV) prodrugs **13** without naphthalimide modified, the positive control cisplatin and oxaliplatin. The cell viability was determined by MTT assays^[a]^

	**Colorectal Cancer**			**Hepatic Cancer**		**Breast Cancer**		**Lung cancer**
	HT-29	HCT-116	CT-26	HepG-2	Huh-7	MDA-MB-231	MCF-7	A549
Naph.	0.09 ± 0.06	0.06 ± 0.06	0.03 ± 0.06	0.45 ± 0.06	1.34 ± 0.13	0.65 ± 0.05	2.21 ± 0.22	8.63 ± 0.15
**13**	16.23 ± 1.52	18.56 ± 1.85	NG	15.48 ± 1.50	16.23 ± 1.28	26.36 ± 2.56	19.48 ± 1.50	NG
Cis.	5.70 ± 0.02	6.78 ± 0.02	5.24 ± 0.02	8.90 ± 0.78	15.02 ± 1.50	32.48 ± 1.49	9.60 ± 0.60	10.20 ± 0.89
FI^[b]^	63.33	113.00	174.67	19.78	11.21	49.90	4.34	0.0090
Oxp.	11.77 ± 1.88	9.26 ± 0.38	6.03 ± 0.02	19.98 ± 1.89	16.17 ± 1.59	17.72 ± 0.89	11.62 ± 0.78	10.00 ±1.23
FI^[c]^	130.78	154.33	201.00	44.40	12.07	27.26	5.26	0.94

*NG* Not Given, *Naph* Naphplatin, *Cis* Cisplatin, *Oxp* Oxaliplatin

^a^An average of three measurements

^b^FI (fold increase) is defined as IC_50_(cisplatin)/IC_50_(Naph.)

^c^FI (fold increase) is defined as IC_50_(oxaliplatin)/IC_50_(Naph.)

Among the broad cancer cell lines including colorectal, hepatic, breast, and lung cancers, naphplatin exhibits lower IC_50_ values in colorectal carcinoma (90 nM for HT-29, 60 nM for HCT-116 and 30 nM for CT-26) than the other cancer cells, which are in accordance with the results of the cellular drug uptake and DNA platinization by ICP-MS (Fig. S44a), flow cytometry scatter plots and up-regulated p53 in vitro (Fig. S44b-e). Based on the above results, we selected colorectal carcinoma cells (CT-26) to perform the in vivo biological assays, including (1) the acute toxicity study, (2) antitumor assays in vivo, (3) anti-metastasis assays in vivo, (4) life extension antitumor assay and (5) macrophage-deficient antitumor assay. Both CT-26 and HCT-116 cells derived from mice and humans, respectively, were selected to perform the in vitro studies on the underlying mechanism.

In the acute toxicity study, we found that compared with cisplatin and oxaliplatin, naphplatin exhibited the highest maximum tolerated dose (MTD, 40.00 mg/kg) and the lethal dose (LD_50_, 78.00 mg/kg) values (Table S7), indicating the feasibility of naphplatin treatment even at high doses. We chose the maximum tolerated concentrations to perform the following in vivo biological assays as previously reported by our group [24–30] and others [3, 4]. The doses of naphplatin were lower than the positive control group to compare anti-tumor growth and metastasis effects in tumour bearing mice. Next, the antitumour assays in vivo (Fig. S45) demonstrated that naphplatin (Inhibitory Rate (IR): 84.35%, 2.79 mg Pt/kg) and naphplatin combined with 5-Fu (IR: 94.20%, 1.87 mg Pt/kg) remarkably suppressed tumour growth at a lower dosage, which was obviously more effective than that of oxaliplatin (IR: 54.20%, 2.95 mg Pt/kg), cisplatin (IR: 57.10%, 3.90 mg Pt/kg) and FOLFOX (oxaliplatin+ 5-Fu, IR: 60.98%) in clinic, suggesting the anticancer efficacy of naphplatin+5-Fu > naphplatin >> oxaliplatin+5-Fu (FOLFOX) > cisplatin > oxaliplatin.

Tumour metastasis is a major obstacle in current chemotherapy and leads to approximately 90% of the cancer-related deaths [35]. To ensure the potency of naphplatin against tumor metastasis in CT-26 and HCT-116 cell lines, pulmonary metastasis, in vitro transwell assays, and the expression of β-catenin expression models were performed. The results of transwell migration and downregulation of β-catenin implied that naphplatin could inhibit colon cancer migration more effectively than cisplatin and oxaliplatin in a dose-dependent manner (Fig. S46). The in vivo anti-metastasis assays indicated naphplatin (IR: 88.12%, 2.79 mg Pt/kg) and naphplatin/5-Fu (IR: 96.70%, 1.87 mg Pt/kg) preferentially accumulated in colorectal carcinoma metastases, which was 4-fold effective than that of oxaliplatin (IR: 20.83%, 2.95 mg Pt/kg), cisplatin (IR: 25.93%, 3.90 mg Pt/kg) and even the FOLFOX regimen alone (IR: 27.82%, 2.95 mg Pt/kg) (Fig. S47).

**Fig. 2:**
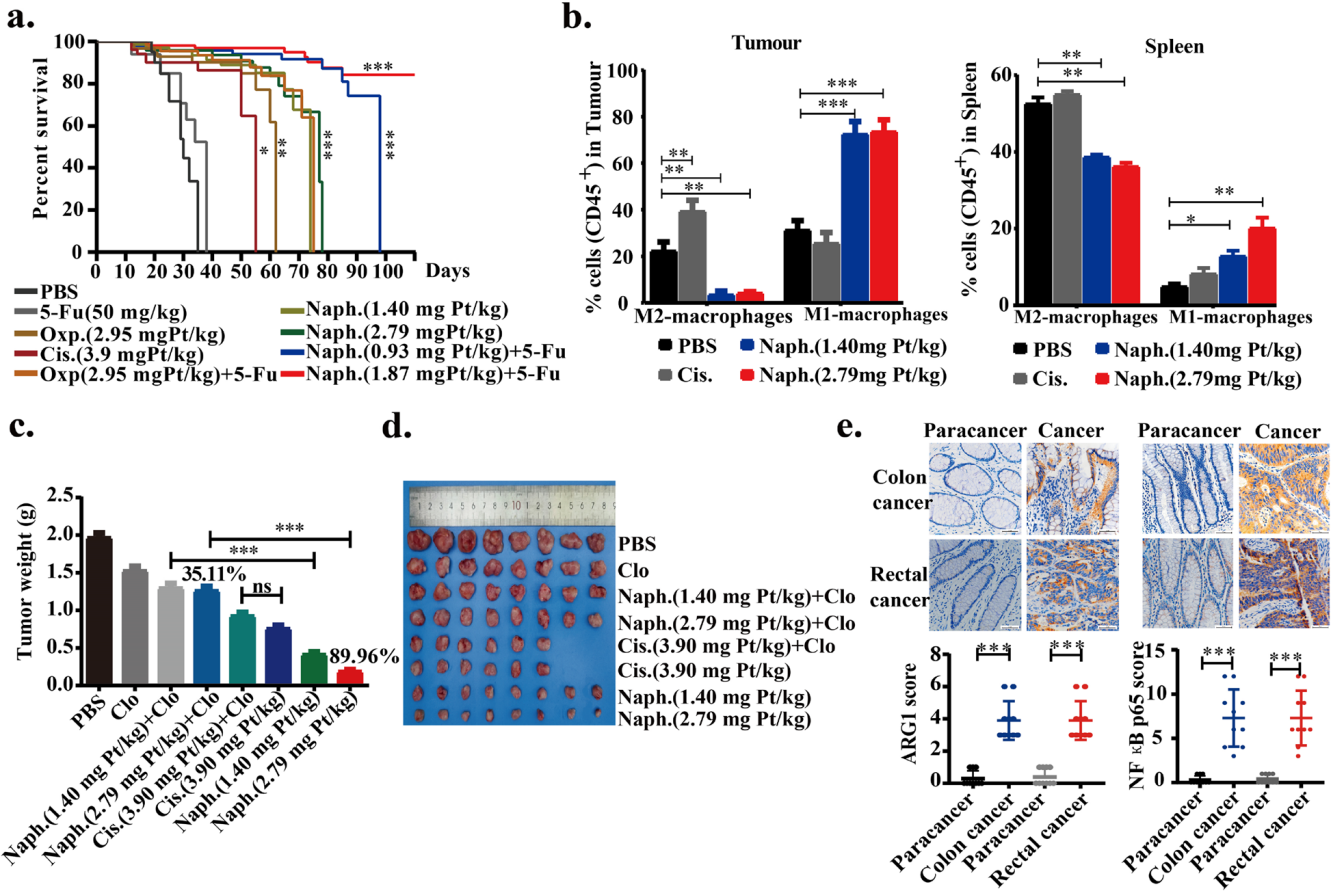
In opposite to cisplatin, naphplatin reinvigorated TAMs to remarkably inhibit colon cancer growth and pulmonary metastasis of mice in vivo. Colorectal carcinoma cells were selected to perform the in vivo biological assays, including (**a**) life extension antitumor assay, (**b**) antitumor assays in vivo, and (**c-d**) macrophage-deficient antitumor assay. **a** Survival of rate with different treatments in the life extension antitumor assay. All the positions marked with stars are compared with PBS, and the results show that Naph. could significantly prolong the survival time of mice. Survival curves are shown. **b** Changes in the proportion of M2-macrophages and M1-macrophages in tumour and spleen of antitumor assays in vivo. BALB/c mice weighing 18−22 g were treated with PBS, 5-Fu, cisplatin, oxaliplatin, naphplatin, oxaliplatin + 5-Fu, naphplatin+ 5-Fu on day 7 after CT-26 cells (3.0-3.5×10^6^ cells per mouse) injected the determination of the in vivo anticancer activity. The drugs dissolved in the glucose solution in each group were injected intra-peritoneally once every 2 days for a total of four treatments. **c** Tumour weight in each group at the end of the in vivo macrophage-deficient antitumor experiments. **d** Images of the tumours at the end of the in vivo macrophage-deficient antitumor experiments. BALB/c mice weighing 18−22 g were treated with drugs on day 7 after CT-26 cells (3.0-3.5×10^6^ cells per mouse). Clodronate liposomes (Clo, 200 μL) were injected intra-peritoneally once every 4 days for a total of four treatments. After another 4 days, the drugs was injected intra-peritoneally once every 2 days for a total of four treatments. **e** The expression of M2 macrophage marker Arg1 and the inflammatory transformation marker NF-κB p65 in 20 clinical specimens of colon and rectal cancer by Immunohistochemistry (IHC). *, *P*< 0.05; **, *P*< 0.01; ***, *P*< 0.001

Next, we investigated the life extension rate because it is an important indicator for overall survival of patients with metastatic cancer. Survival curves from the life extension antitumor assay demonstrated that life-prolonging rates in naphplatin (2.79 mg Pt/kg, Life Prolonging Rate (LPR): 82.71%) and naphplatin+5-Fu (1.87 mg Pt/kg, LPR: 136.84%) groups were much higher than those in cisplatin (3.90 mg Pt/kg, LPR: 19.17%), oxaliplatin (2.95 mg Pt/kg, LPR: 45.11%) and oxaliplatin+5-Fu (LPR: 67.29%) groups over 90 days of treatment (Fig. 2a and Table S8).

### In contrast to cisplatin, naphplatin reinvigorated TAMs to remarkably inhibit colon cancer growth and pulmonary metastasis in mice in vivo

To investigate the roles of the immune system in the antitumor activity of naphplatin, we assessed the capacity of naphplatin to induce immunogenic cell death (ICD) by detecting the hallmarks of ICD [12, 36]. After treatment of CT-26 colorectal carcinoma cells with naphplatin (Fig. S48a-b), three ICD biomarkers were observed, including HMGB1 release from the nuclei to the cytoplasm, CRT exposure, and a higher extracellular ATP secretion (151.67 nM) than the intracellular level (139.34 nM), which had not been observed in the cisplatin group [36].

To further explore the relationship between antitumour activity and immune cells, the blood, tumour and spleen tissues (Fig. S47d) in the above antitumor assays *in vivo* in Fig. S45 were collected and analyzed by flow cytometry, to determine macrophages, M1/M2-macrophages, CD45^+^, CD19^+^ B cells, CD5^+^ T cells, CD4^+^ T cells, CD8^+^ T cells, Treg, dendritic cells, mMDSCs, and gMDSCs. Surprisingly, in both the tumour and spleen, the number of M1 macrophages (iNOS^+^) was remarkably increased while the number of M2 macrophages (CD206) was reduced in naphplatin-treated tumour-bearing mice in comparison to the control. In contrast, in the cisplatin group, the macrophages in the spleen significantly decreased and the M2 macrophages increased, suggesting that cisplatin can induce severe tumour immunosuppression (Fig. 2b). Using flow cytometric analysis, we also found that the numbers of CD11b^+^LY6C^+^LY6G^-^M-MDSCs, CD11b^+^LY6C^-^LY6G^+^G-MDSC and CD4^+^CD25^+^Foxp3^+^T-reg cells in the tumour microenvironment were remarkably reduced, indicating that naphplatin treatment may terminate the vicious TAMs-MDSC-Treg triangle [37], thus promoting the antitumor immunity.

To further investigate whether the naphplatin-induced antitumor effects are dependent on macrophages, clodronate liposomes (Clo) as macrophage scavenger were intra-peritoneally injected into mice to remove macrophages without affecting T cells (Fig. S48c), and the clearance of macrophages in the spleen was also confirmed by flow cytometry (Fig. S48d). Our results discovered that naphplatin-mediated antitumour activities could be severely disrupted by macrophage depletion (Fig. 2c, d). Specifically, upon treatment with the combination of naphplatin and Clo (IR: 35.11%), the tumour weight increased by 54.85% in the naphplatin group (IR: 89.96%), which was not observed in the cisplatin group (IR: 61.31%) and the combination group of cisplatin and Clo (IR: 52.67%), suggesting macrophages play an essential role in the antitumour efficacy of naphplatin. Furthermore, we found that the number of M1 macrophages increased, while that of M2 macrophages decreased in naphplatin-treated tumour-bearing mice, which indicates the prevention of immunosuppression by naphplatin. In contrast, severe tumour immunosuppression was observed in the spleen and tumours after cisplatin treatment (Fig. S48e).

To support potential clinical translation of these laboratory findings, we studied the functions and phenotypes of macrophages in human tissue samples. We employed 10 pairs of clinical specimens of colon and rectal cancer to detect the expressions of the M2 macrophage marker Arg1 (Fig. 2e) and the inflammatory transformation marker NF-κB p65 *via* immunohistochemistry. Among them, the NF-κB p65 marker can induce transformation from the M2 to M1 phenotype. The immunohistochemical results indicated that both markers were expressed at higher levels in all types of cancer cell samples than in paracancers, proving that regulation of macrophage function may be an important strategy to prevent colon cancer metastasis. Moreover, the negligible change in body and organ weights suggested acceptable toxicity of naphplatin (Fig. S48f, g).

These mechanism studies indicate naphplatin could preferentially alleviate cancer metastases and ameliorate tumour immunosuppression by regulating the ratio of M2 and M1 phenotypes, which is different to the mechanism of action of cisplatin.

### Naphplatin could reset both BMDM and M2-BMDM to M1 phenotype

**Fig. 3:**
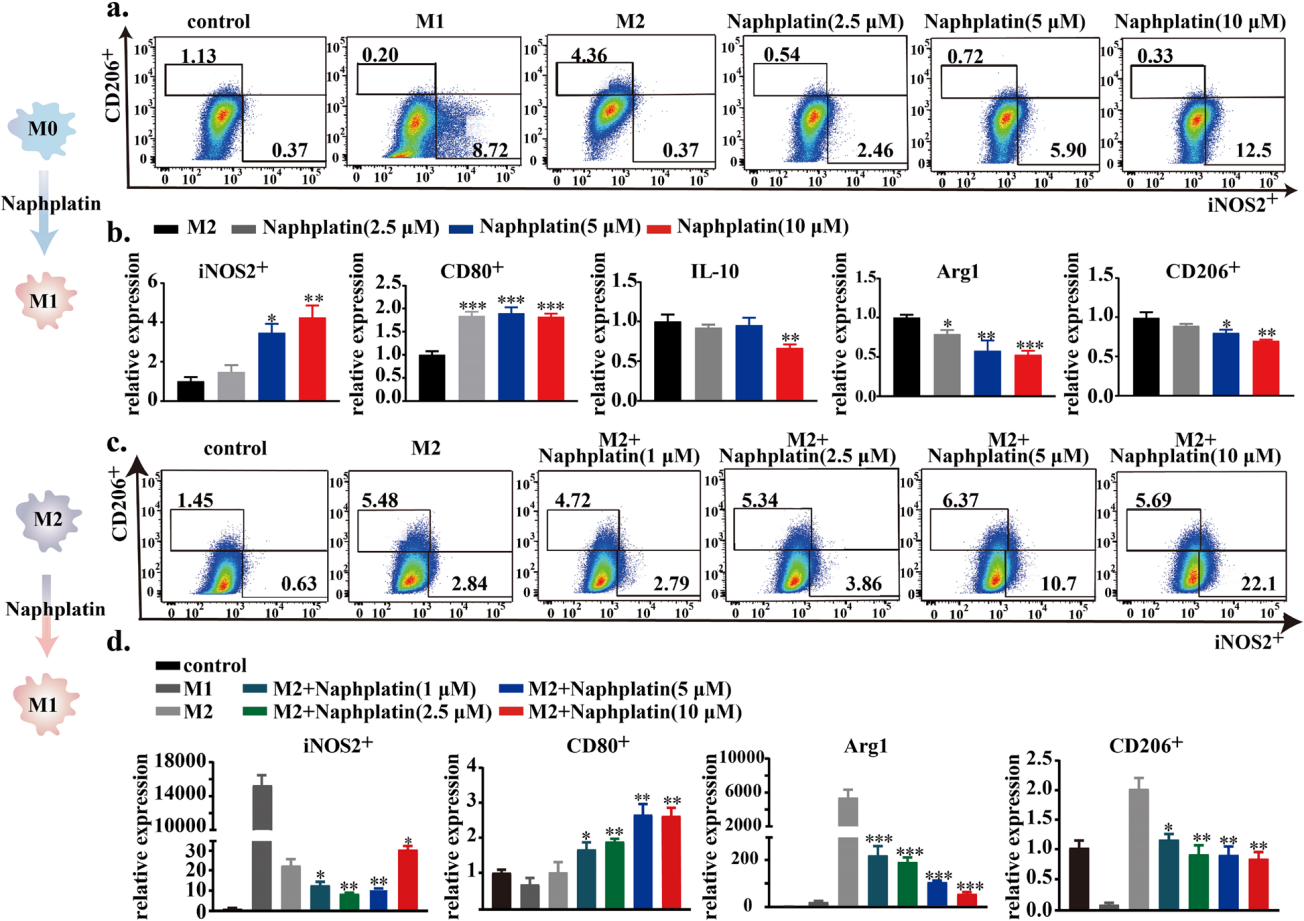
Reset of both BMDM and M2-BMDM to M1 phenotype by naphplatin. BMDM and M2-BMDM cells were treated with or without 2.5 μM, 5 μM, 10 μM naphplatin**,** LPS (100 ng/mL) or IL-4 (20 ng/mL). **a** Representative flow cytometric analysis and quantification of iNOS and CD206 in BMDM isolated from mice. **b** The mRNA expression of iNOS2, CD80, IL-10, Arg1 and CD206 in BMDM cells with or without naphplatin treatment by qPCR. **c** Representative flow cytometric analysis and quantification of iNOS and CD206 in M2-BMDM. **d** The mRNA expression of iNOS2 and CD80 in BMDM-M2 cells and Arg1, CD206 in BMDM-M1 cells with or without naphplatin treatment by qPCR. *, *P* < 0. 05 **, *P* < 0.01 ***, *P* < 0.001

Since naphplatin could strongly induce ICD, which in turn triggers an immune response to promote the therapeutic outcome [36]. We next evaluated the capacity of naphplatin to boost the immune response, especially for macrophages. BMDM were first isolated and stimulated with LPS (20 ng/mL) to generate M1 macrophages and IL-4 (100 ng/mL) to produce M2 macrophage [36]. Then, we explored whether naphplatin can transform BMDM (Fig. 3a, b and Fig. S49a) and M2-BMDM (Fig. 3c, d and Fig. S49b-d) to M1 phenotype. Compared with LPS (proportion: 8.72%) and IL-4 (proportion: 4.36%) groups, naphplatin treatment prominently up-regulated the expression of M1 macrophages marker iNOS2 (proportion: 12.50%) and down-regulated M2 marker CD206 (proportion: 0.33%). Moreover, naphplatin remarkably enhanced the expressions of inducible nitric oxide synthase (iNOS, an M1 marker) and CD80, while inhibiting M2-TAMs markers of immunosuppressive factors including IL-10, arginase 1 (Arg1, an M2 marker), and CD206 determined by PCR and western blotting analysis. In addition to directly inducing the BMDM to M1 phenotype, naphplatin could also promote similar expression of the above biomarkers in readily IL-4-conditioned M2 macrophages. These conclusive results demonstrate that naphplatin could regulate the transformation from BMDM and M2-BMDM to M1 phenotype to ameliorate tumour immuno-suppression.

### Lysosome-mediated Mcoln1 and MAPK activation for M2 macrophage polarization

**Fig. 4:**
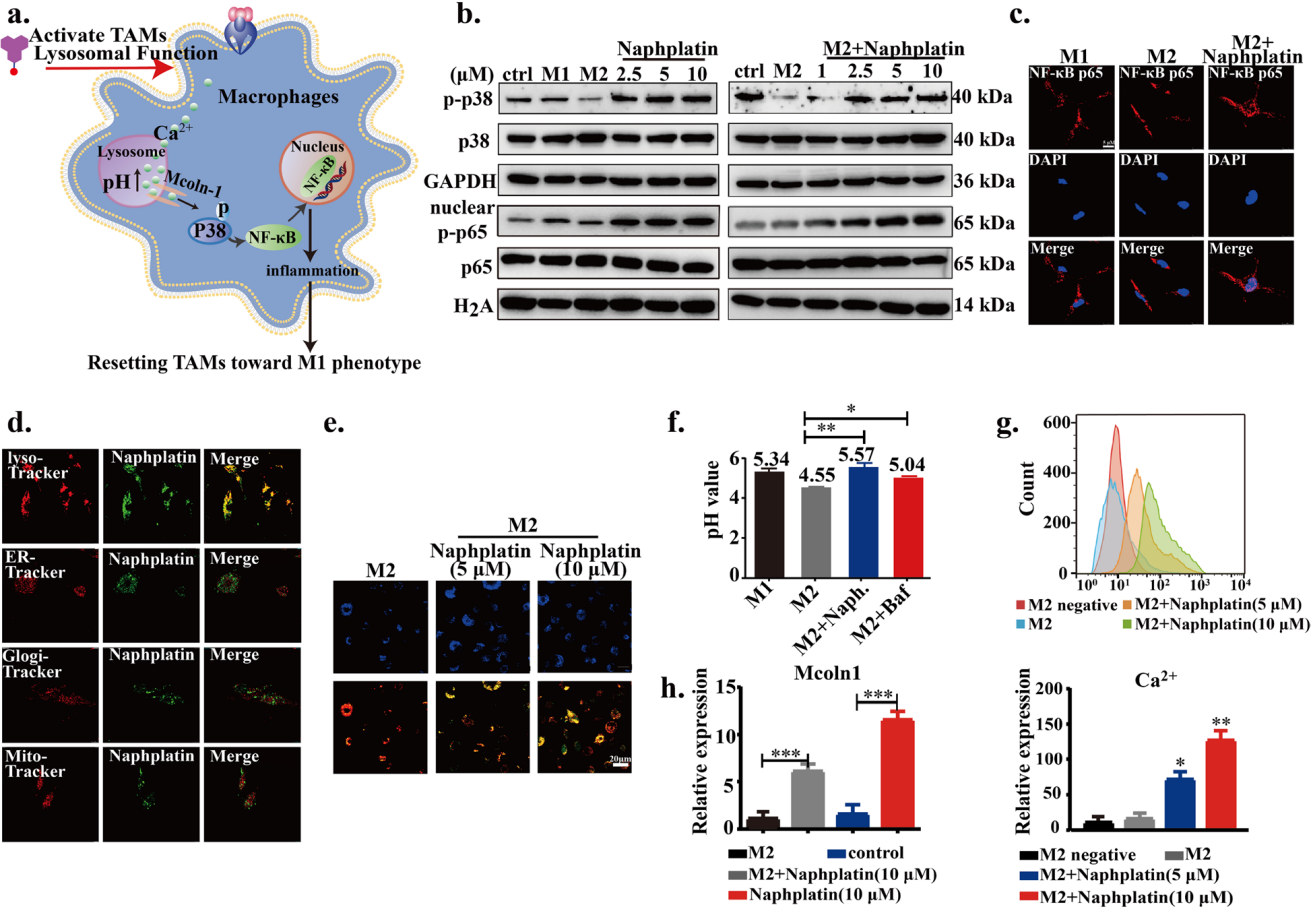
Lysosome-mediated Mcoln-1 and MAPK activation for M2 macrophage polarization. **a** Representational mechanism diagram of naphplatin-reset macrophages from M2 to M1 phenotype to ameliorate tumour immuno-suppression. **b** The expressions of p-p38, total p38, nuclear p-p65, and total p65 in BMDM and M2-BMDM cells with or without naphplatin treatment by western blotting. **c** Nuclear translocation of NF-κB p65 in BMDM-M2 cells with or without naphplatin treatment by confocal fluorescent microscope. Red, NF-κB p65; Blue, DAPI. Scale bar, 5 μm. **d** Images of naphplatin’s sub-organelle localization in M2-BMDM by confocal microscopy. M2-BMDM was treated with naphplatin (10 μM) for 2 h followed by staining with Mito-tracker, Golgi-tracker, ER-tracker, and Lyso-traker. **e** Images of the pH values of macrophage lysosome in M2-macrophage, M2-macrophage+ naphplatin (5 μM or 10 μM) *via* LysoSensor DR (Blue/Orange) kit, Blue is pH independent for cellular localization; Red indicates more acid pH, and Orange indicates acid pH. **f** The change of macrophage lysosomes pH values by confocal microscopy in M1-macrophage, M2-macrophage, M2-macrophage+ naphplatin (10 μM) or M2-macrophage+ Baf. **g** The mean fluorescence intensity (MFI) of Fluo-4 AM (**g**, up) and the statistics (**g**, down) of Ca^2+^ in BMDM-M2 cells treated with or without naphplatin. **h** The mRNA expression of Mcoln1 in BMDM-M2 cells in the presence and absence of naphplatin (10 μM)(*n* = 3). *, *P* < 0. 05 **, *P* < 0.01 ***, *P* < 0.001

Next, we found that naphplatin locates in lysosomes to increase macrophage lysosomal pH and then triggers Ca^2+^ release *via* Mcoln-1, which induces the activation of MAPK p38, but not Erk, and nuclear factor-κB (NF-κB) for M2 macrophage polarization. The probable mechanism diagram of naphplatin is firstly summarized in Fig. 4a. The mitogen-activated protein kinase (MAPK) signaling pathway is well known to be activated in LPS-induced M1 macrophages [38, 39] and is the key regulator of pro-inflammatory factors [31]. To confirm the role of MAPK in naphplatin-induced antitumor immunity, we analyzed the related protein changes in MAPK signaling pathway, including p38, Erk. Surprisingly, we found that only p38 phosphorylation (Fig. 4b and Fig. S49e, f) but not Erk (Fig. S49g, h) was up-regulated in M2-BMDM and BMDM cells after naphplatin treatment, implying that p38 might be involved in the switch of macrophage phenotype. Then, NF-κB p65 was tested by checking its nuclear translocation and the results demonstrated that naphplatin treatment resulted in the entry of NF-κB p65 into the nucleus of BMDM and M2-BMDM (Fig. 4b, c and Fig. S49g, h).

Based on the natural fluorescence properties of naphplatin (Fig. S50a), M2-BMDM was selected to verify the subcellular organelle localization by laser confocal microcopy and the results revealed that naphplatin predominately accumulated in lysosomes (Fig. 4d). It is well known that changing the pH value of macrophage lysosomes can switch TAMs from the M2 to tumour-killing M1 phenotype. We were interested in measuring the changes in pH values in the lysosome after treatment with naphplatin, an alkaline drug. To this end, M2 were first treated with or without Naphplatin for 24 h, followed by incubation with LysoSensor DR for another 30 min. The confocal microscopy results showed that, with the increasing concentration of naphplatin, the lysosome pH values were elevated with colour changing from red to orange, suggesting a less acidic pH in the Naphplatin treated group (Fig. 4e). In addition, by staining lysosomes with Lyso Sensor Green, we confirmed that naphplatin treatment could increase M2 lysosomal pH values from 4.55 to 5.57, which was similar to that of LPS-polarized M1 macrophages (pH 5.34) and the lysosomal targeted agent bafilomycin A1 (Baf) group (Fig. 4f). Although Baf could increase the lysosomal pH value in IL-4 induced M2 macrophages from 4.55 to 5.04, it was less able to increase iNOS expression.

Notably, it is well documented that increasing the pH of M2 macrophages triggers lysosomes to release calcium [40]. Along with increased lysosomal pH, intracellular calcium concentration was also elevated in naphplatin-treated M2 macrophages (Fig. 4g up and down). Several Ca^2+^ channels have been known to mediate lysosomal calcium release, mucolipin-1 (Mcoln1), a major lysosomal calcium release channel [41], was found to be upregulated by Naphplatin (Fig. 4h). Moreover, the addition of cyclosporin A (CsA), a Ca^2+^ signaling inhibitor, obviously blocked the effect of naphplatin at 5 μM (Fig. S50b-d) or 10 μM (Fig. S50e-g) on the macrophage phenotype switch and p38 phosphorylation, indicating that Mcoln1-mediated lysosomal calcium release induced the activation of p38 for M1 macrophage polarization.

### HMGB1-mediated CTSL-lysosome function blockade in colorectal cancer

**Fig. 5:**
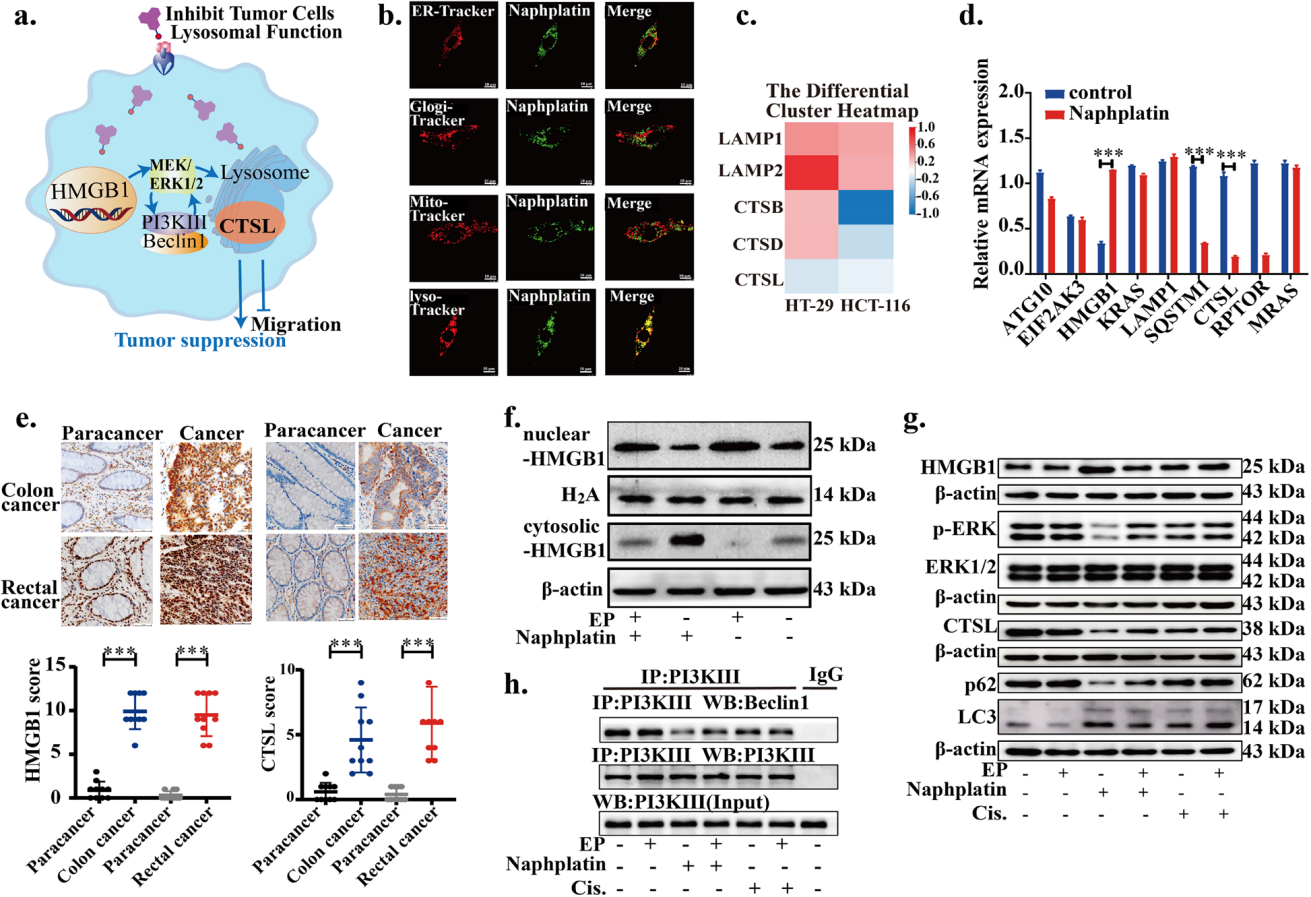
HMGB1-mediated CTSL-lysosome function blockade in colorectal cancer. **a** Representational mechanism diagram of naphplatin-reset lysosomal function of tumour cells, activated HMGB1-mediated autophagy through Beclin-1- PI3K-III/MEK/ERK signal pathway to deter cancer metastases and immune evasion. **b** Images of naphplatin’s sub-organelle localization in CT-26 cells by confocal microscopy. CT-26 cells were treated with naphplatin (10 μM) for 2 h followed by staining with Mito-tracker, Golgi-tracker, ER-tracker, and Lyso-traker. **c** The expression of genes related to lysosomal function and autophagy from RNA-seq analysis. **d** The mRNA expression of genes related to the common apelin pathways using real-time PCR in colorectal cancer cells (HT-29 and HCT-116) treated with naphplatin for 24 h. **e** The expression of HMGB1 and CTSL in 20 clinical specimens of colon and rectal cancer by immunohistochemistry. **f** Whole cell lysates, nuclear extracts and cytoplasmic fractions of HMGB1 by western blot in CT-26 cells pre-treated with or without ethyl pyruvate (EP, 10 mM, 1 h) before addition of naphplatin (10 μM) for 24 h. **g** The expression of HMGB1, p-ERK, ERK1/2, CTSL, p62, and LC3II/LC3I in CT-26 cells treated with 10 μM naphplatin or cisplatin in the presence or absence of EP (10 mM, 2 h) by western blot. **h** Changes of Beclin-1-PI3K-III core complex in CT-26 cells treated with 10 μM naphplatin or cisplatin in the presence or absence of EP (10 mM, 2 h) by co-immunoprecipitatio. *P< 0.05, **P< 0.01, ***P< 0.0001

Based on the above observations, we found that in contrast to cisplatin, naphplatin-induced antitumor immunogenic cell death (ICD) is macrophage-dependent, in which macrophages are the key to antitumour and anti-metastasis efficacy of naphplatin. Next, we further explored the remaining effects of naphplatin-mediated changes in tumour cells. We found that by promoting HMGB1 cytosolic translocation and inhibition of Beclin-1-PI3K-III core complex formation through the MEK/ERK1/2 pathway, naphplatin modulates CTSL and autophagy-lysosome function and enhances its sensitivity towards colorectal cancer (Fig. 5a).

Inspection of the subcellular organelles by laser confocal microscopy suggested that both naphplatin (Fig. 5b) and the released ligand 11a (Fig. S51a) were also selectively locate in the lysosomes in colon cancer cells. To further investigate the distribution of cellular naphplatin, ICP-MS analysis was employed, and it was found that platinum accumulation in the whole cells was 5 times greater than those observed for cisplatin and oxaliplatin. The intracellular distribution of platinum in ER, lyso, mito and nuclear DNA indicated that naphplatin-mediated antitumour effects resulted from synergistic inhibition of lysosomal function (32.00%) and nuclear DNA (42.70%) in colon cancer cells (Fig. S51b).

RNA sequencing (RNA-seq) was used to further profile the transcriptome to explore the underlying key mechanism of how naphplatin affects colorectal cancer. Among significantly altered genes, 13,748 intersecting genes were involved in all samples (Fig. S51c-f). The expressions of genes related to the lysosomes metabolic pathway including CTSL was found to decrease in naphplatin-treated cells compared to control cells (Fig. 5c). Next, we focused on the changes in the expression of genes associated with the first 20 pathways (Fig. S51d) and the lysosomal metabolic pathway after naphplatin treatment (Fig. S51f). The intersection of these pathways indicates the gene expressions associated with HMGB1, SQSTM1, and CTSL were significantly altered. Real-time PCR assays further confirmed that the gene expressions of HMGB1 and CTSL were remarkably decreased in naphplatin-treated cells (Fig. 5d). IHC in 20 clinical specimens of colon and rectal cancer also verified the expressions of HMGB1 and CTSL (Fig. 5e) were higher in colon cancer than in paracancerous tissues, raising the necessity to determine the changes associated with HMGB1-mediated CTSL and autophagy-lysosome pathway.

As we all know, HMGB1 is a highly conserved non-histone nuclear protein and functions in the cytoplasm as an extracellular signaling protein in inflammation, cell differentiation, tumour progression, cisplatin resistance, and induces the hallmarks of ICD [41–43]. CTSL up-regulation has been widely identified and correlated with metastatic aggressiveness and poor patient prognosis of colon cancer [44]. Nevertheless, the detailed molecular mechanism of HMGB1-mediated CTSL-lysosome function in colon cancer therapy and platinum drugs has not been clearly defined.

Therefore, we investigated naphplatin modulate HMGB1-meditated CTSL and autophagy-lysosome programs modulated by naphplatin in colorectal cancer. CT-26 and HCT-116 cells derived from mice and humans respectively, were selected to study the underlying mechanism of action. Western blot analysis of CT-26 and HCT-116 cells (Fig. S52a-d) revealed that naphplatin treatment led to a dose-dependent increase in the level of LC3-II and a decrease in p62, two selective markers of autophagy, which were consistent with the immunofluorescence results (Fig. S52b). Next, to determine the roles of naphplatin-induced autophagy in the drug treatment, CT-26 cells were cultured with an autophagy inhibitor, 3-methyladenine (3-MA), before addition of naphplatin. The sensitivity of CT-26 cells to naphplatin was markedly enhanced while the proliferation rate was greatly diminished after adding 3-MA (Fig. S52c). In addition to the above findings, we noticed that naphplatin could up-regulate the expression of HMGB1 in the colon cancer lines (Fig. S52a-d), which was in accordance with the previous observations (Fig. S48a). Our results indicated that naphplatin can effectively inhibit the gene and protein expressions in the MEK-ERK1/2 pathway (Fig. S52a-d) and the formation of the Beclin-1-PI3K-III complex by co-immunoprecipitation in CT-26 and HCT-116 cells (Fig. S52e), which is essential for vesicle nucleation in autophagic stages [42–45].

As indicated in Fig. S48a, naphplatin could induce the translocation of HMGB1 from the nucleus to cytoplasm to induce the hallmarks of ICD. We next explore the potential role of HMGB1 in the regulation of naphplatin-induced autophagy and apoptosis. Western blot analysis of nuclear and cytosolic fractions of CT-26 cells indicated that the HMGB1 levels in cytosolic were elevated after exposure to naphplatin (Fig. 5f and S52f). To clarify the role of HMGB1 in cytosolic translocation during chemotherapy, we analyzed the responses of colorectal cancer cells to naphplatin by adding ethyl pyruvate (EP), a pharmacological inhibitor of HMGB1 cytoplasmic translocation (Fig. S52c, g, 5g, h, S53, and S54), and observed an enhanced cell proliferation and migration in CT-26 (Fig. S53a, b, and S54a, c, d) and HCT-116 (Fig. S53c, d and S54b, e, f) cells. Ethyl pyruvate (EP), a pharmacological inhibitor of HMGB1 cytoplasmic translocation, can reverse the proapoptotic effect of naphplatin, which can not be observed in cisplatin group. Moreover, EP also inhibited HMGB1 cytosolic translocation in CT-26 and HCT-116 cells (Fig. 5f and S52f), however, we surprisingly found that naphplatin-induced CTSL and autophagy**-**lysosome function was reversed and Beclin-1-PI3K-III core complex formation was inhibited through the MEK/ERK1/2 pathway (Fig. 5g,h and S53e,f). In addition, the results of MEK/ERK1/2 pathway U0126 (Fig. S52h, i ) further confirmed that autophagy was also significant for naphplatin-apoptosis and migration. All these results suggest that naphplatin can promote HMGB1 cytosolic translocation and inhibits Beclin-1-PI3K-III core complex formation through the MEK/ERK1/2 pathway to modulate CTSL and autophagy-lysosome function, which therefore enhances the sensitivity of colorectal cancer to naphplatin.

## Discussion

To date, the promise of immunotherapy, including chimeric antigen receptor T cell therapy or checkpoint inhibitor monoclonal antibody (CIMA), is limited in treating “hot” cancers and has largely bypassed the greater majority of patients with solid organ cancers, considered immunologically “cold” [46–48]. In these tumours, an immune milieu and an abundance of immune-evasive cues are generated to induce an exhausted T cell phenotype or exclude cytotoxic T cells usually involving innate immune cells. Strategies that reinvigorate innate immune cells including TAMs, which can be attracted and reprogrammed by tumor cells to support tumour growth and metastatic spread, are underrepresented within current immuno-oncology therapies [49]. Remodeling the TAMs phenotype that occupies the main population of tumour-infiltrating immune cells, is the major challenge in the development of anti-TAM therapies and platinum-containing chemotherapy.

Currently, many studies focus on how to deplete M2 TAMs, to block CCL2/CCR2 recruitment pathway or to interfere with M2-related signaling pathways such as CD47/SIRPα [50, 51]. However, our present findings provide an alternative strategy of targeting M2 TAMs, which possesses several advantages as follows: (1) The increased potency of tumour cell lines to induce IL-10-producing M2 macrophages also limited the clinical use of platinum-containing chemotherapy anti-metastatic activities and blocked many anticancer platinum complexes moving from the laboratory to clinic [3–7]. In contrast to traditional platinum drugs, we demonstrated that immune elements dominate the antitumor efficacy of novel naphplatin’s antitumor efficacy, which induced antitumor ICD in a macrophage-dependent manner to reinvigorate TAMs and remarkably inhibit cancer growth and pulmonary metastasis of mice in vivo. (2) Naphplatin does not deplete M2 macrophages but instead reset them into M1 phenotype, thus better utilizing macrophages to reconstruct antitumor immune microenvironment. (3) Distinct from currently available immune-modulatory antibodies, naphplatin being a small chemical compound is easily distributed in tumor microenvironment to target TAMs. (4) In the present study, drugs that can target TAMs, such as chloroquine and Baf [52] could increase the lysosomal pH value in IL-4 induced M2 macrophages, but were less able to increase iNOS expression or less effective in anti-metastasis of mice. (5) We revealed how and why lysosome metabolism promotes antitumor immunity to terminate vicious TAMs-MDSCs-Treg triangle by exploring the inherent lysosomal metabolic plasticity. All in all, our findings provide new insight into the anticancer mechanisms of lysosome blockade in macrophages and tumours for cancer immunotherapy and identify naphplatin as a tumor immunotherapeutic agent.

An important finding in this study is that lysosome blockade can induce divergent metabolic programs in macrophages polarization and tumours metastasis for cancer immunotherapy. Phagocytosis of extracellular materials and their effective degradation in lysosomes are core tasks of macrophages. As a membrane-bound organelle, lysosomes are spherical vesicles that contain various hydrolytic enzymes that can break down all kinds of biomolecules [53]. However, this degradation process is dependent on the acidic pH value inside the lysosomal lumen [54]. Consequently, compared with M1-type macrophages, altering lysosomal pH value of M2 macrophages is an effective strategy to reset the macrophage subtype and functions. In addition, targeting lysosomes is also a promising cancer treatment strategy in tumour cellular processes such as signal transduction, autophagy and apoptosis. In this study, we provide the first example of naphplatin deterring platinum-containing chemotherapy immune evasion and cancer metastases *via* resetting HMGB1-mediated lysosomal function of tumour cells and macrophages.

The decreased antitumor effects in macrophage-deficient (34.25%) and natural (89.96%) mice indicate naphplatin-induced antitumor ICD is macrophage dependent, which cannot be observed in cisplatin group. We speculate that immune elements actually dominate naphplatin’s antitumor and anti-metastasis efficacy. Conversely, naphplatin preferentially accumulates in lysosome to remodel macrophage from tumor-promoting M2 to tumor-inhibiting M1 phenotype by increasing lysosomal pH value, which is totally different from cisplatin. The elevated pH values in M2-BMDM triggering Mcoln1-mediated lysosomal calcium release, induces the activation of p38 and NF-κB for M1 macrophage polarization. Moreover, the numbers of M-MDSC, G-MDSC and T-reg cells within the tumour microenvironment are remarkably reduced in naphplatin-treated tumor bearing mice, indicating naphplatin terminates the vicious TAM-MDSC-Treg triangle and promotes antitumor immunity, which was not observed in the cisplatin group.

In addition to its role in the nucleus, HMGB1 functions in the cytoplasm as an extracellular signaling protein during inflammation, cell differentiation, tumour progression, cisplatin resistance and induces the hall marks of ICD. Up to date, CTSL levels could be of diagnostic significance in colon cancer. Interestingly, for cancer cells, we found for the first time that HMGB1-mediated CTSL and autophagy-lysosome pathway by RNA-seq is strongly linked to the effects of naphplatin-mediated antitumor and anti-metastasis effects. All above results are employed to clarify the remaining effects in naphplatin-mediated changes tumour cells to dismantle metastases and the immunosuppressive microenvironment of cancers, as well as the exact molecular mechanism of HMGB1-mediated CTSL and autophagy-lysosome function in the tumour therapy of platinum drugs.

Previous reports [55, 56] on the novel platinum family with naphthalimide ligands focused on DNA-targeting properties and ignored the exploration of resetting lysosomal metabolic programs in tumour cells and macrophages. Collectively, in addition to establishing divergent lysosomal metabolic programs both in tumour cells and macrophages, our studies illuminate a previously unrecognized macrophage-based tumor immunotherapeutic modality of chemotherapy, which is totally different from traditional platinum-based antitumour drugs by inhibition of DNA [56]. Moreover, through the application of lysosomal metabolism blockade in cancer cells and activation in macrophage, we demonstrated the possibility of differential modulation of the metabolism of cancer cells and anti-tumour immune cells by exploring the inherent metabolic plasticity of each cell type. Our results provides a basis for future investigation of divergent metabolic programs as a strategic target for platinum-containing chemotherapy or combination with immunotherapy to deter cancer metastases and immune evasion in the clinic. This work offers new avenues for designing highly efficient anticancer drugs and advances our understanding of anticancer mechanisms in metal complex-based chemotherapy and currently available immune-modulatory antibodies. The results offer myriad opportunities to boost drug discoveries, facilitate clinical translations, and improve healthcare outcomes.

## Conclusion

In summary, we developed a novel anticancer drug (naphplatin) based on a naphthalimide-platinum structure and discovered a new anticancer mechanism in metal complex-based chemotherapy. Our studies illuminate a previously unrecognized macrophage-based tumour immunotherapeutic modality of chemotherapy to treat bypassed immunologically “cold” patients. This work offers avenues for designing highly efficient anticancer drugs and advances our understanding of anticancer mechanisms in metal complex-based chemotherapy, providing myriad opportunities to boost drug discoveries, facilitate clinical translations and finally improve healthcare outcomes.

### Supplementary Information

**Additional file 1.** 

## Data Availability

Supplementary information, chemical compound information and source data are available from the corresponding authors on reasonable request.

## Abbreviations

BMDM: Bone marrow derived macrophage
Mcoln1: Mucolipin-1
MAPK: Mitogen-activated protein kinase
HMGB1: High-mobility group box 1
CTSL: Cathepsin L
NF-κB: Nuclear factor-κB
Clo: Clodronate liposomes
RNA-seq: RNA sequencing
Treg: Regulatory T cell
ICD: Immunogenic cell death
3-MA: 3-Methyladenine
CIMA: Checkpoint inhibitor monoclonal antibody
iNOS: Inducible nitric oxide synthase
ICP-MS: Inductively coupled plasma-mass spectrometry
IR: Inhibitory Rate
LPR: Life Prolonging Rate
TAMs: Tumor-associated macrophages
