# Supplementary material for: Lysosome blockade induces divergent metabolic programs in macrophages and tumours for cancer immunotherapy
Source: J Exp Clin Cancer Res. 2023 Aug 4;42:192. doi: 10.1186/s13046-023-02768-0 (PMC10401909; doi:10.1186/s13046-023-02768-0)
Supplement: Supplementary file 1 — Additional file 1. [file 13046_2023_2768_MOESM1_ESM.doc]

**Supporting Information**

Lysosome blockade induces divergent metabolic programs in macrophages and tumours for cancer immunotherapy

Jing Ma,^1,7^ Ruijuan Ma,^1,7^ Xueke Zeng,^2,7^ Liming Zhang,^2^ Hanjing Niu,^2^ Jianing Liu,^2^ Wei Zhang,^1^ Guochen Bao,^6^ Chaojie Wang,^3^ Peng George Wang,^5^ Jiajia Wang,^2^,**^*^**Xia Li,^2^,**^*^** Taotao Zou,^4^ and Songqiang Xie,^1^,**^*^**

^1^ School of Pharmacy, Institute of Chemical Biology, Henan University, Kaifeng, Henan, 475004, (China)

^2^ Joint National Laboratory for Antibody Drug Engineering, Henan University, Kaifeng, Henan, 475004 (China)

^3^ The Key Laboratory of Natural Medicine and Immuno-Engineering, Henan University, Kaifeng, 475004(China)

^4^ School of Pharmaceutical Sciences Sun Yat-Sen University, Guangzhou, Guangdong, 510006 (China)

^5^ School of Medicine, The Southern University of Science and Technology, Shenzhen, Guangdong, 518005 (China)

^6^ Institute for Biomedical Materials and Devices (IBMD), Faculty of Science, University of Technology Sydney, Sydney, New South Wales, Australia

^7^ These authors contributed equally to this work.

*Correspondence: [jwang577@163.com](mailto:jwang577@163.com)(J. J. W); [lixia_kf@126.com](mailto:lixia_kf@126.com)(X. L); [xiesq@vip.henu.edu.cn](mailto:xiesq@vip.henu.edu.cn)(S. Q. X)

**Experimental Procedures**

**Reagents and instruments**

All reagents were used as received without further purification. Cisplatin and oxaliplatin were purchased from Yurui Chemical Co. Ltd. (Shanghai, China). All other chemicals were obtained from commercial suppliers, such as Alfa Aesar, Aldrich, J&K, and GL Biochem Ltd. and were of analytical grade. If necessary, the reactions were conducted in dry solvents and under an argon atmosphere. ^1^H NMR and ^13^C NMR spectra were recorded on a Bruker AVANCE AV300 (300 MHz). High-resolution mass spectra (HRMS) were obtained on an IonSpec QFT mass spectrometer with ESI ionization. 3-(4,5-dimethylthiazol-2-yl)-2,5–diphenyl tetrazolium bromide (MTT), ascorbic acid (AsA), 5’-GMP (purity ≥ 98.0%), DMEM and RPMI 1640 medium containing 10% foetal bovine serum were purchased from GL Biochem Ltd. A Genomic DNA mini preparation kit from Beyotime (China) was used for cellular drug uptake and DNA platination experiments. Phosphate-buffered saline (PBS) contained 137 mM NaCl, 2.7 mM KCl, 10 mM Na_2_HPO_4_ and 2 mM KH_2_PO_4_. Foetal bovine serum (FBS), 0.25% trypsin/EDTA solution, and penicillin-streptomycin solution were purchased from Invitrogen (Grand Island, NY, USA). A549cisR cells were maintained with 2 μg/mL cisplatin. HPLC analyses were performed on a Waters E2695-2998 system equipped with a Venusil MP C18 column (150 × 4.6 mm, 5 μm). HPLC profiles were recorded with a UV detector at 273 nm and a fluorescence detector at an excitation wavelength of 340 nm and an emission wavelength of 515 nm at room temperature. The mobile phase consisted of MeOH and H_2_O and was used at a flow rate of 0.5 mL/min (20 μL injection volume). Reactions involved in the preparation of platinum compounds were conducted in the dark. Platinum levels were examined by inductively coupled plasma mass spectrometry (ICP-MS, PE Elan 6100 DRC or PE Nexion 2000).

BALB/c mice (28-42 d) were purchased from the Laboratory Animal Center, Academy of Military Medical Science (Beijing, China). All of the animals received care in compliance with the guidelines outlined in the Guide for the Care and Use of Laboratory Animals. The projects in vivo were all approved by the ethical committee (HUSOM-2016-316). We determined the anticancer and anti-metastatic activities by healthy BALB/c mice aged 5 weeks (18−22 g) with the number of Cat. SCXK 2016-0006, Beijing, China from the Laboratory Animal Center, Academy of Military Medical Science.

**Synthesis and characterization**. The preparations of **7a-7c**, **12a-12d** and **14a,** naphplatin were summarized in Scheme S1-S6. As shown in Figure S1 and Scheme S1, the axial ligands were designed containing the classical core of amonafide or mitonafide in clinic, which were common active alkaline moieties potentially to reset lysosomal function. Naphthalimide intermediates **5a-5c** and **11a-11d** in Scheme S6 were prepared as previously reported by our group**^1-2^** and their structures were also fully characterized (Figures S1–S42). The purity of final platinum complexes was confirmed to be ≥ 95% by analytical HPLC (Table S1 and S2). Considering the possibility of many reducing agents in cells, we measured their stabilities in water and RPMI 1640, and the results showed that they were stable at least for 48 h (Figure S43).

**In vivo MTD and LD_50_ antitumor assays.** MTD were calculated by mean body weight loss <15% and <15% toxic deaths. LD_50_ and TI values were calculated according to previous reported1.

***In vivo* antitumor and anti-metastasis assays.** Purchased from the Laboratory Animal Center, Academy of Military Medical Science, Beijing, China, BALB/c mice (Cat. SCXK 2016-0006) weighed 18−22 g and aged 5 weeks, were raised in compliance with the guide for the care of laboratory animals published by Henan University. And all animal experiments were approved by the ethical committee (HUSOM-2016-316).

For the *in vivo* antitumor assay, CT-26 cells (3.0 × 10^5^-3.5 × 10^5^ cells per mouse) were injected subcutaneously and then inoculated for 7 days. And when tumours grows to 80-120 mm^3^, BALB/c mice were divided into 11 groups, including control group, 5-Fu (50 mg/kg) group, cisplatin (3.90 mg Pt/kg) group, oxaliplatin (2.95 mg Pt/kg) group, **12a**(2.50 mg Pt/kg) group, naphplatin(2.79 mg Pt/kg) group, oxaliplatin + 5-Fu group, **12a**(1.25 mg Pt/kg) + 5-Fu group, **12a**(2.50 mg Pt/kg) + 5-Fu group, naphplatin(0.93 mg Pt/kg) + 5-Fu group, naphplatin(1.87 mg Pt/kg) + 5-Fu group. And then the drugs dis-solved in glucose solution in each group were injected intra-peritoneally once every 2 days for a total of four treatments (n = 8 mice per group). We measured mice weight and the volume of the tumours every two days. To the end, the mice were sacrificed and the tumour tissues were weighed to calculate the inhibition rate as follows. Inhibitory rate (%) = [(the average tumour weight of the control group − the average tumour weight of the drug-treated or positive group)/ the average tumour weight of the control group] × 100%). And in order to further probe the interaction between anti-tumour effects and immune system, the tumour, blood and spleen samples in anti-tumour model were analysed by flow cytometry to explore the changes in the proportion of macrophages, M2-macrophages, M1-macrophages, CD45^+^, CD19^+^B cells, CD5^+^Tcells, CD4^+^Tcells, CD8^+^Tcells, Treg, Dendritic, mMDSCs and gMDSCs.

For *in vivo* anti-metastasis assay, CT-26 cells (3.0 × 10^5^-3.5 × 10^5^ cells per mouse) were injected *via* the tail vein and also inoculated for 7 days. And then drugs was dissolved in glucose solution and injected intra-peritoneally once every 2 days for a total of four treatments (n = 8 mice per group). To the end, the mice were sacrificed, the lung metastasis nodules were fixed with 4% paraformaldehyde and counted to calculate the inhibition rate as follows. Inhibitory rate (%) = [(the average nodules of the control group − the average nodules of the drug-treated or positive group group)/the average nodules of the control group × 100 %].

***In vivo* life extension antitumor assay.** Purchased from the Laboratory Animal Center, Academy of Military Medical Science, Beijing, China, BALB/c mice (Cat. SCXK 2016-0006) weighed 18−22 g and aged 5 weeks, were raised in compliance with the guide for the care of laboratory animals published by Henan University. And all animal experiments were approved by the ethical committee (HUSOM-2016-316). CT-26 cells (3.0 × 10^5^-3.5 × 10^5^ cells per mouse) were injected subcutaneously and then inoculated for 7 days. And when tumours grows to 80-120 mm^3^, BALB/c mice were divided into 9 groups, including control group, 5-Fu (50 mg/kg) group, cisplatin (3.90 mg Pt/kg) group, oxaliplatin (2.95 mg Pt/kg) group, naphplatin(2.79 mg Pt/kg) group, oxaliplatin + 5-Fu group, naphplatin(0.93 mg Pt/kg) + 5-Fu group, naphplatin(1.87 mg Pt/kg) + 5-Fu group. And then the drugs dis-solved in glucose solution in each group were injected intra-peritoneally once every 2 days for a total of four treatments (n = 8 mice per group). The time of death of mice were observed and recorded, and the average survival days were calculated. The life prolonging rate was calculated according to the average survival days. Life prolonging rate (%) = [(the average survival days of the control group − the average survival days of the drug-treated or positive group)/ the average survival days of the control group] × 100%).

***In vivo*** **macrophage-deficient antitumor assay.** For *in vivo* macrophage-deficient antitumor assay, CT-26 cells (3.0 × 10^5^-3.5 × 10^5^ cells per mouse) were injected subcutaneously and then inoculated for 4 days. BALB/c mice were divided into 8 groups, including control group, Clo group as macrophage scavenger, naphplatin(1.40 mg Pt/kg) + Clo group, naphplatin(2.79 mg Pt/kg) + Clo group, cisplatin (3.90 mg Pt/kg) + Clo group, cisplatin (3.90 mg Pt/kg) group, naphplatin(1.40 mg Pt/kg) group and naphplatin(2.79 mg Pt/kg) group. Clo (200 μL) was also injected intra-peritoneally at ten o 'clock in the morning once every 4 days for a total of four treatments (n = 8 mice per group). After another 4 days, when tumours grows to 80-120 mm^3^, the drugs including naphplatin and cisplatin was injected intra-peritoneally at five in the afternoon once every 2 days for a total of four treatments (n = 8 mice per group). Mice weight and the volume of the tumours were measured every two days. To the end, the mice were sacrificed to calculate the inhibition rate as follows. Inhibitory rate (%) = [(the average tumour weight of the control group − the average tumour weight of the drug-treated or positive group)/ the average tumour weight of the control group] × 100%). And in order to further probe the interaction between anti-tumour effects and macrophage, the tumour, blood and spleen samples were analysed by flow cytometry to explore the changes in the proportion of macrophages, M2-macrophages, M1-macrophages, CD45^+^, CD19^+^B cells, CD5^+^Tcells, CD4^+^Tcells, CD8^+^Tcells, Treg, Dendritic, mMDSCs and gMDSCs.

**Method for purification of target compounds.** The purities of all target compounds were determined by HPLC. The HPLC analyses were performed on a Waters E2695-2998 instrument equipped with a Venusil MP C18 column (150×4.6 mm, 5 μm). The purity of the platinum complexes (**7a-7c**, **12a-12d** and **14a, naphplatin**) was confirmed to be ≥95% by analytical HPLC. The method and purities results of target compounds were as follows (Table S1 and Table S2). The final concentration of their compounds in cell samples was also checked as the method for purity determination of target compounds by RP-HPLC at room temperature in dark.

**Table S1. Method for purification of target compounds.**

| Time (min) | A (water) | B (methanol) |
| --- | --- | --- |
| 0 | 90% | 10% |
| 5 | 90% | 10% |
| 35 | 0% | 100% |
| 45 | 0% | 100% |

**Table S2. Purities results of target compounds**

| Compounds | R.T. | Purity(%) | Compounds | R.T. | Purity(%) |
| --- | --- | --- | --- | --- | --- |
| **7a** | 6.80 | 95.79 | **12c** | 6.50 | 95.49 |
| **7b** | 6.60 | 96.83 | **12d** | 6.49 | 95.73 |
| **7c** | 6.59 | 96.69 | **14a** | 6.48 | 95.00 |
| **12a** | 6.40 | 95.14 | **naphplatin** | 6.39 | 95.16 |
| **12b** | 6.40 | 95.14 |  |  |  |

**Determination of the apparent oil–water partition coefficient.** The shaking flask method was used for determination. A proper amount of naphplatin was dissolved in water-saturated N-octanol, the 1.2 pH hydrochloric acid solution, and the 2.0, 5.0, 6.8, and 7.4 pH phosphate buffers, respectively. Thus, a series of drug-saturated N-octanol solutions were prepared. A volume of 1.0 mL of each of the above solutions was measured precisely and put into 6 plug test tubes; next, 4 mL of water and the corresponding N-octanol-saturated pH buffer were successively added. After vortexing for 5 min and shaking at (37 ± 2) °C on a constant temperature oscillation box for 48 h, the tubes were taken out and kept standing still for 30 min. After this, the two phases were separated by 10,000 r/min centrifugation for 10 min. The samples were diluted with the mobile phase 10 times and analysed by HPLC method. Allmeasurements were conducted in triplicate. The concentration of naphplatin and the log P_app_ were calculated(Table S3). The calculation formula was as follows: P_app_ = C_oil_/C_water_ = 4*(Ct)/(C_0_-C_t_)

In the above equation, P_app_ was the apparent oil–water partition coefficient; C_0_ was the initial concentration of the drug in N-octanol; and C_t_ was the concentration measured in the oil phase at the equilibrium partition of the drug.

**Table S3. Determination of the apparent oil–water partition coefficient.**

| Solvent | log P_app_ |
| --- | --- |
| Water | 2.71 |
| Hydrochloric acid solution pH1.2 | 1.79 |
| Phosphate buffer pH2.0 | 0.95 |
| Phosphate buffer pH5.0 | 0.74 |
| Phosphate buffer pH6.8 | 0.74 |
| Phosphate buffer Ph7.4 | 0.78 |

**Statistical analyses.** Statistical significance was examined via an unpaired two-tailed Student’s t-test by the Graphpad 6.0 software and the results were presented as mean ± SEM. Long-term survival curve was analyzed by the log-rank (Mantel–Cox) test. The P-value < 0.05 was considered as statistically significant. All the data are presented as the mean ± SD and analyzed using Student’s t test or analysis of variance (ANOVA) followed by q-test. *p < 0.05, **p < 0.01, and ***p < 0.001 were considered to be statistically significant.

**Scheme S1**. Chemical structures and synthetic route of **7a-7c**, **12a-12d** and **14a**, naphplatin in yield of 25%-30%. Reagents and conditions: (a) Na_2_Cr_2_O_7_, AcOH, 100℃; (b) HNO_3_, 50℃, 3 h; (c) Pd/C, H_2_, MeOH, rt, 2 h; (d) EtOH, 85℃, 5 h; (e) DMF, TBTU,TEA, rt, 24h; (f) HNO_3_, 50℃, 3 h.

**General Procedure for the Synthesis of Compound 15a**.

**Scheme S2**. Chemical structures and synthetic route of **15a**.

The key step involved the preparation of oxoplatin **15a** which was reacted successively in water (12 mL) with cisplatin (0.2 g) and H_2_O_2_ (20 mL) at 60 °C for 4 h with yield of 70% as our previous reported ^1^.

**General Procedure for the Synthesis of Compound 15b.**

**Scheme S3**. Chemical structures and synthetic route of **15b**.

The key step involved the preparation of oxoplatin **15b** which was reacted successively in water (12 mL) with oxaliplatin (0.2 g) and H_2_O_2_ (20 mL) at 60 °C for 4 h with the yield of 75% as our previous reported ^1^.

**General Procedure for the Synthesis of Compound 16a.**

**Scheme S4.** Chemical structures and synthetic route of **16a**.

In our case, we used cis, cis, trans-[PtCl_2_(NH_3_)_2_ (palmitate)] (**16a**) as the precursor for the synthesis of the conjugates. **16a** was prepared from oxoplatin **15a** (1 equiv.) and Palmitic anhydride (4 equiv.) in anhydrous DMSO at r.t. for 7 days. DMSO was removed under vacuum to afford a yellow oil. And then the oil was washed with dichloromethane and diethylether, and dried in a vacuum. The target complex **16a** was obtained as a pale yellow solid after purification by recrystallization with the yield of 89% as our previous reported ^1^.

**General Procedure for the Synthesis of Compound 16b.**

**Scheme S5.** Chemical structures and synthetic route of **16b**.

In our case, we used **16b** as the precursor for the synthesis of the conjugates. **16b** was prepared from oxoplatin **15b** (1 equiv.) and Palmitic anhydride (4 equiv.) in anhydrous DMSO at r.t. for 7 days. DMSO was removed under vacuum to afford a yellow oil. And then the oil was washed with dichloromethane and diethylether, and dried in a vacuum. The target complex **16b** was obtained as a pale yellow solid after purification by recrystallization with the yield of 90% as our previous reported ^1^.

**General Procedure for the Synthesis of Compound 5a-5c and 11a-11d.**

**Scheme S6.** Chemical structures and synthetic route of naphthalimide intermediates **5a-5c**, and **11a-11d** in yield of 55%-70%. Reagents and conditions: (a) Na_2_Cr_2_O_7_, AcOH, 100℃; (b) HNO_3_, 50℃, 3 h; (c) Pd/C, H_2_, MeOH, rt, 2 h; (d) EtOH, 85℃, 5 h; (e) HNO_3_, 50℃, 3 h.

Known intermediate **3** and **10a-10b** was prepared by a conventional method using concentrated nitric acid in glacial acetic acid **^2^**. To a solution of **3** or **10a-10b** (1 mmol) and K_2_CO_3_ in EtOH (10 mL) was added a solution containing **4** (1 mmol) in EtOH (5 mL) at 0 °C. Then the mixture was heated to 85 °C for 5 h. After monitoring by TLC, the reaction mixture was cooled to room temperature and concentrated under vacuum to give an oily residue. After extraction and purification by column chromatography with dichloromethane/methanol (100:1 −100:3, v/v) as elution solvent, **5a-5c** and **11a-11d** was obtained in yield of 60-70%.

**5a**, ^1^H NMR (300 MHz, DMSO-*d*_6_) δ 8.07 – 7.86 (m, 3H), 7.57 (t, *J* = 7.8 Hz, 1H), 7.25 (s, 1H), 5.94 (s, 2H), 4.21 (t, *J* = 7.7 Hz, 2H), 2.56 (t, *J* = 7.6 Hz, 2H). ^13^C NMR (75 MHz, DMSO-*d*_6_) δ 172.99 , 164.10 , 163.90 , 148.19 , 133.91 , 131.96 , 127.36 , 125.93 , 122.87 , 122.05 , 121.01 , 112.33 , 36.11 , 32.74 . ESI-MS (positive ion mode): m/z [M]^－^: calcd: 284.08; obsd: 282.95. Elemental Analysis for C_15_H_12_N_2_O_4_ C 63.38%, H 4.26%, N 9.85%; Found C 63.36%, H 4.16%, N 9.80%.

**5b**, ^1^H NMR (300 MHz, DMSO-*d*_6_) δ 12.07 (s, 1H), 8.00 (dd, *J* = 12.8, 7.7 Hz, 2H), 7.94 (d, *J* = 2.3 Hz, 1H), 7.56 (t, *J* = 7.8 Hz, 1H), 7.25 (d, *J* = 2.3 Hz, 1H), 5.99 (s, 2H), 4.04 (t, *J* = 6.9 Hz, 2H), 2.30 (t, *J* = 7.3 Hz, 2H), 1.87 (t, *J* = 7.1 Hz, 2H). ^13^C NMR (75 MHz, DMSO-*d*_6_) δ 174.48 , 164.29 , 164.12 , 148.24 , 133.92 , 131.79 , 127.26 , 125.78 , 122.99 , 122.18 , 122.12 , 112.09 , 40.47 , 39.64 , 31.81 , 23.52 . ESI-MS (positive ion mode): m/z [M]^－^: calcd: 298.09; obsd: 296.96. Elemental Analysis for C_16_H_14_N_2_O_4_ C 64.42%, H 4.73%, N 9.39%; Found C 64.30%, H 4.70%, N 9.40%.

**5c**, ^1^H NMR (300 MHz, DMSO-*d*_6_) δ 12.35 – 11.64 (m, 1H), 8.01 (ddd, *J* = 21.3, 14.2, 8.0 Hz, 3H), 7.73 – 7.46 (m, 1H), 7.27 (d, *J* = 8.9 Hz, 1H), 6.00 (d, *J* = 8.7 Hz, 2H), 3.99 (dt, *J* = 14.2, 7.5 Hz, 2H), 2.23 (dt, *J* = 11.1, 7.1 Hz, 2H), 1.58 (dq, *J* = 17.6, 8.2 Hz, 4H), 1.35 (t, *J* = 8.5 Hz, 2H). ^13^C NMR (75 MHz, DMSO-*d*_6_) δ 174.98 , 164.09 , 148.35 , 131.94 , 127.40 , 125.86 , 123.00 , 122.23 , 112.23 , 34.05 , 27.85 , 26.52 , 24.70 . ESI-MS (positive ion mode): m/z [M]^－^: calcd: 326.12; obsd: 325.15. Elemental Analysis for C_18_H_18_N_2_O_4_ C 66.25%, H 5.56%, N 8.58%; Found C 66.30%, H 5.46%, N 8.56%.

**11a**, ^1^H NMR (300 MHz, DMSO-*d*_6_) δ 8.59 (d, *J* = 8.3 Hz, 1H), 8.40 (d, *J* = 7.2 Hz, 1H), 8.16 (d, *J* = 8.3 Hz, 1H), 7.63 (t, *J* = 7.8 Hz, 1H), 6.84 (d, *J* = 8.4 Hz, 1H), 4.20 (t, *J* = 7.8 Hz, 2H), 2.88 (d, *J* = 7.2 Hz, 2H). ^13^C NMR (75 MHz, DMSO-*d*_6_) δ 173.62 , 134.40 , 131.51 , 130.11 , 129.80 , 124.46 , 122.08 , 119.76 , 108.64 , 36.11 , 33.46. ESI-MS (positive ion mode): m/z [M]^－^: calcd: 284.08; obsd: 282.95. Elemental Analysis for C_15_H_12_N_2_O_4_ C 63.38%, H 4.26%, N 9.85%; Found C 63.36%, H 4.16%, N 9.80%.

**11b**, ^1^H NMR (300 MHz, DMSO-*d*_6_) δ 8.55 (d, *J* = 8.4 Hz, 1H), 8.38 (d, *J* = 7.3 Hz, 1H), 8.15 (d, *J* = 8.4 Hz, 1H), 7.61 (t, *J* = 7.8 Hz, 1H), 6.83 (d, *J* = 8.4 Hz, 1H), 4.03 (d, *J* = 6.9 Hz, 2H), 2.26 (t, *J* = 7.4 Hz, 2H), 1.84 (t, *J* = 7.1 Hz, 2H). ^13^C NMR (75 MHz, DMSO-*d*_6_) δ 174.54 , 164.39 , 163.52 , 153.07 , 134.37 , 131.49 , 129.64 , 124.45 , 122.11 , 119.74 , 108.65 , 108.01 , 39.14 , 31.82 , 23.66 . ESI-MS (positive ion mode): m/z [M]^－^: calcd: 298.09; obsd: 296.96. Elemental Analysis for C_16_H_14_N_2_O_4_ C 64.42%, H 4.73%, N 9.39%; Found C 64.30%, H 4.70%, N 9.40%.

**11c**, ^1^H NMR (300 MHz, DMSO-*d*_6_) δ 11.93 (s, 1H), 8.59 (dd, *J* = 8.4, 1.2 Hz, 1H), 8.39 (dd, *J* = 7.3, 1.1 Hz, 1H), 8.17 (d, *J* = 8.4 Hz, 1H), 7.62 (dd, *J* = 8.4, 7.3 Hz, 1H), 6.83 (d, *J* = 8.4 Hz, 1H), 3.98 (t, *J* = 7.4 Hz, 2H), 2.21 (t, *J* = 7.3 Hz, 2H), 1.61 – 1.50 (m, 4H), 1.33 (q, *J* = 5.6, 4.3 Hz, 2H). ^13^C NMR (75 MHz, DMSO-*d*_6_) δ 174.89 , 164.17 , 163.31 , 153.10 , 134.34 , 131.37 , 129.67 , 124.35 , 122.21 , 119.80 , 108.59 , 108.03 , 40.80 , 39.69 , 33.97 , 27.89 , 26.56 , 24.72 . ESI-MS (positive ion mode): m/z [M]^－^: calcd: 326.12; obsd: 325.15. Elemental Analysis for C_18_H_18_N_2_O_4_ C 66.25%, H 5.56%, N 8.58%; Found C 66.30%, H 5.46%, N 8.56%.

**11d**, ^1^H NMR (300 MHz, DMSO-*d*_6_) δ 7.38 (d, *J* = 7.9 Hz, 1H), 7.27 (dt, *J* = 16.0, 6.8 Hz, 3H), 6.79 (t, *J* = 7.5 Hz, 1H), 2.99 (t, *J* = 7.5 Hz, 2H), 1.37 (d, *J* = 7.5 Hz, 2H). ^13^C NMR (75 MHz, DMSO-*d*_6_) δ 175.86 , 166.02 , 165.22 , 152.25 , 134.96 , 133.34 , 132.88 , 132.00 , 131.37 , 127.48 , 125.85 , 125.71 , 39.37 , 35.38 . ESI-MS (positive ion mode): m/z [M]^－^: calcd: 326.12; obsd: 325.15. Elemental Analysis for C_15_H_10_N_2_O_6_ C 57.33%,H 3.21%, N 8.91%; Found C 57.40%,H 3.30%, N 8.87%.

**General procedure for the synthesis of compound 7a-7c, 12a-12d and 14a, naphplatin.**

**Synthetic route of 7a.**

To a solution of **5a** (276 mg) in DMF (20 mL) was added a DMF solution (0.5 mL) containing TBTU (0.57 mmol). This mixture stirred for 10 min at room temperature. A DMF solution containing **4** (1.5 mmol) and DIPEA (0.92 mmol) was added to the resulting solution. The mixture was stirred at room temperature for 24 h in the dark. The DMF was then removed under vacuum to afford a yellow oil. Compound **7a** was purified by silica gel column chromatography as a yellow solid in 54% yield. ^1^H NMR (300 MHz, Chloroform-d) δ 8.44 (s, 2H), 7.77 (s, *J* = 8.5 Hz, 2H), 6.32 (s, 3H), 3.20 (t, *J* = 6.7 Hz, 5H), 2.42 (dd, 2H), 2.17 (t, *J* = 18.3 Hz, 2H), 1.87-1.54 (m, *J* = 26.2 Hz, 3H), 1.40-1.26 (m, *J* = 19.4 Hz, 28H), 0.83 (t, 3H). ^13^C NMR (75 MHz, Chloroform-d) δ 165.42, 165.42, 138.96, 127.35, 126.40, 116.21, 111.78, 47.03, 31.89, 29.34, 29.17, 25.70, 22.65, 14.08, 8.76. ESI-MS (positive ion mode): m/z [M]^＋^: calcd: 935.3644; obsd: 936.3702. Elemental Analysis for C_39_H_56_N_4_O_10_Pt C 50.05%, H 6.03%, N 5.99%; Found C 50.10%, H 6.08%, N 6.04%.

**Synthetic route of 7b.**

To a solution of **5b** (276 mg) in DMF (20 mL) was added a DMF solution (0.5 mL) containing TBTU (0.57 mmol). This mixture stirred for 10 min at room temperature. A DMF solution containing **4** (1.5 mmol) and DIPEA (0.92 mmol) was added to the resulting solution. The mixture was stirred at room temperature for 24 h in the dark. The DMF was then removed under vacuum to afford a yellow oil. Compound **7b** was purified by silica gel column chromatography as a yellow solid in 64% yield. 1H NMR (300 MHz, Chloroform-d) δ 8.07 (s, J = 7.3 Hz, 1H), 7.91 – 7.85 (m, 2H), 7.66 (m, J = 28.3, 5.3 Hz, 8H), 3.19 – 2.96 (m, 11H), 2.54 (dd, 2H), 2.28 (dt, J = 8.5 Hz, 4H), 1.42 – 1.29 (m, 4H), 1.28-1.23 (m, J = 7.3 Hz, 11H), 1.21-1.10 (m, 24H), 0.76 (t, J = 6.5 Hz, 7H). 13C NMR (75 MHz, Chloroform-d) δ 168.74, 154.93, 144.22, 130.53, 120.80, 50.81, 45.87, 43.80, 42.41, 35.82, 33.60, 26.58, 17.94, 12.60. ESI-MS (positive ion mode): m/z [M]^＋^: calcd: 949.3800; obsd: 950.3835. Elemental Analysis for C_40_H_58_N_4_O_10_Pt C 50.57%, H 6.15%, N 5.90%; Found C 50.60%, H 6.18%, N 5.94%.

**Synthetic route of 7c.**

To a solution of **5c** (276 mg) in DMF (20 mL) was added a DMF solution (0.5 mL) containing TBTU (0.57 mmol). This mixture stirred for 10 min at room temperature. A DMF solution containing **4** (1.5 mmol) and DIPEA (0.92 mmol) was added to the resulting solution. The mixture was stirred at room temperature for 24 h in the dark. The DMF was then removed under vacuum to afford a yellow oil. Compound **7c** was purified by silica gel column chromatography as a yellow solid in 56% yield. 1H NMR (300 MHz, Methanol-d4) 7.86 – 7.41 (m, 5H), 3.49 (t, 2H), 2.48 (dd, 2H), 2.35 (dt, 4H), 1.70 – 1.55 (m, 14H), 1.51-1.13 (m, J = 3.2 Hz, 26H), 0.85 (t, J = 6.4 Hz, 7H). 13C NMR (75 MHz, Chloroform-d) δ 126.71, 126.41, 110.89, 42.09, 41.89, 39.85, 29.61, 25.66, 22.59, 13.96. ESI-MS (positive ion mode): m/z [M]^＋^: calcd: 977.4113; obsd: 978.4168. Elemental Analysis for C_42_H_62_N_4_O_10_Pt C 51.58%, H 6.39%, N 5.73%; Found C 51.60%, H 6.48%, N 5.94%.

**Synthetic route of 12a.**

To a solution of **11a** (276 mg) in DMF (20 mL) was added a DMF solution (0.5 mL) containing TBTU (0.57 mmol). This mixture stirred for 10 min at room temperature. A DMF solution containing **4** (1.5 mmol) and DIPEA (0.92 mmol) was added to the resulting solution. The mixture was stirred at room temperature for 24 h in the dark. The DMF was then removed under vacuum to afford a yellow oil. Compound **12a** was purified by silica gel column chromatography as a yellow solid in 57% yield. 1H NMR (300 MHz, Chloroform-d) δ 8.29 – 8.13 (m, 3H), 7.51 (d, J = 7.7 Hz, 2H), 3.28 (t, J = 11.0 Hz, 2H), 2.71 (t, 2H), 2.34 (dd, J = 7.6 Hz, 2H), 2.28 – 1.80 (m, 6H), 1.60 – 0.94 (m, 22H), 0.77 (t, J = 6.5 Hz, 3H). 13C NMR (75 MHz, Methanol-d4) δ 173.95, 164.42, 151.22, 134.31, 131.60, 128.09, 124.46, 109.03, 39.20, 31.64, 29.61, 23.42, 13.96. ESI-MS (positive ion mode): m/z [M]^＋^: calcd: 935.3644; obsd: 936.3702. Elemental Analysis for C_39_H_56_N_4_O_10_Pt C 50.05%, H 6.03%, N 5.99%; Found C 50.10%, H 6.08%, N 6.04%.

**Synthetic route of 12b.**

To a solution of **11b** (276 mg) in DMF (20 mL) was added a DMF solution (0.5 mL) containing TBTU (0.57 mmol). This mixture stirred for 10 min at room temperature. A DMF solution containing **4** (1.5 mmol) and DIPEA (0.92 mmol) was added to the resulting solution. The mixture was stirred at room temperature for 24 h in the dark. The DMF was then removed under vacuum to afford a yellow oil. Compound **12b** was purified by silica gel column chromatography as a yellow solid in 53% yield. 1H NMR (300 MHz, Chloroform-d) δ 8.14 (d, J = 7.3 Hz, 1H), 8.05 (s, 1H), 7.96 – 7.88 (m, 2H), 7.82 (s, 2H), 7.57 (s, 3H), 4.37 (s, 4H), 3.63 (t, J = 4.3 Hz, 2H), 3.26 (dd, J = 26.8 Hz, 4H), 2.76 (t, J = 4.2 Hz, 2H), 2.45 (dt, J = 33.4 Hz, 4H), 2.06 (t, 2H), 1.89-1.57 (m, 7H), 1.49-1.12 (m, J = 10.8 Hz, 28H), 0.97 (t, J = 5.7 Hz, 3H). 13C NMR (75 MHz, Methanol-d4) δ 176.30, 141.29, 134.11, 126.95, 125.89, 117.19, 110.08, 29.41, 22.35, 13.09, 7.85. ESI-MS (positive ion mode): m/z [M]^＋^: calcd: 949.3800; obsd: 950.3835. Elemental Analysis for C_40_H_58_N_4_O_10_Pt C 50.57%, H 6.15%, N 5.90%; Found C 50.60%, H 6.18%, N 5.94%.

**Synthetic route of 12c.**

To a solution of **11c** (276 mg) in DMF (20 mL) was added a DMF solution (0.5 mL) containing TBTU (0.57 mmol). This mixture stirred for 10 min at room temperature. A DMF solution containing **4** (1.5 mmol) and DIPEA (0.92 mmol) was added to the resulting solution. The mixture was stirred at room temperature for 24 h in the dark. The DMF was then removed under vacuum to afford a yellow oil. Compound **12c** was purified by silica gel column chromatography as a yellow solid in 47% yield. 1H NMR (300 MHz, Chloroform-d) δ 8.28 (d, J = 8.4 Hz, 1H), 8.09 (s, J = 8.4 Hz, 1H), 7.99 (d, J = 7.8 Hz, 1H), 7.83 (d, J = 8.3 Hz, 2H), 4.67 (s, 6H), 3.93 (t, 2H), 3.35 (dd, J = 1.6 Hz, 2H), 2.97 – 2.59 (m, 4H), 1.61 (ddd, J = 43.7, 20.6, 6.3 Hz, 12H), 1.27 – 1.15 (m, 30H), 0.88 (t, J = 6.5 Hz, 5H). 13C NMR (75 MHz, Methanol-d4) δ 184.02, 164.60, 126.77, 110.90, 46.80, 31.83, 29.60, 29.56, 29.26, 23.85, 22.58, 13.87. ESI-MS (positive ion mode): m/z [M]^＋^: calcd: 977.4113; obsd: 978.4168. Elemental Analysis for C_42_H_62_N_4_O_10_Pt C 51.58%, H 6.39%, N 5.73%; Found C 51.60%, H 6.48%, N 5.94%.

**Synthetic route of 12d.**

To a solution of **11d** (276 mg) in DMF (20 mL) was added a DMF solution (0.5 mL) containing TBTU (0.57 mmol). This mixture stirred for 10 min at room temperature. A DMF solution containing **4** (1.5 mmol) and DIPEA (0.92 mmol) was added to the resulting solution. The mixture was stirred at room temperature for 24 h in the dark. The DMF was then removed under vacuum to afford a yellow oil. Compound 4a1 was purified by silica gel column chromatography as a yellow solid in 57% yield.1H NMR (300 MHz, Methylene Chloride-d2) δ 8.76 – 8.39 (m, 4H), 8.36 – 8.17 (m, 1H), 4.28 (t, J = 6.4 Hz, 2H), 2.81 – 2.46 (m, 4H), 2.16 (dd, J = 7.3 Hz, 2H), 1.39 – 1.15 (m, 38H), 0.83 (t, J = 6.7 Hz, 3H). 13C NMR (75 MHz, Chloroform-d) δ 163.13, 162.33, 138.60, 130.97, 126.42, 115.94, 111.95, 31.93, 29.34, 19.17, 14.13, 8.83.

**Synthetic route of 14a.**

To a solution of **11d** (276 mg) in DMF (20 mL) was added a DMF solution (0.5 mL) containing TBTU (0.57 mmol). This mixture stirred for 10 min at room temperature. A DMF solution containing **4** (1.5 mmol) and DIPEA (0.92 mmol) was added to the resulting solution. The mixture was stirred at room temperature for 24 h in the dark. The DMF was then removed under vacuum to afford a yellow oil. Compound 4a1 was purified by silica gel column chromatography as a yellow solid in 57% yield. 1H NMR (300 MHz, Chloroform-d) δ 8.78 (d, J = 9.5 Hz, 1H), 8.69 – 8.56 (m, 2H), 8.35 (t, J = 9.0 Hz, 1H), 2.55 (t, J = 7.8 Hz, 2H), 2.32 – 2.09 (m, 2H), 1.59-1.50 (m, J = 39.8, 9.5 Hz, 4H), 1.16 (s, J = 25.8 Hz, 24H), 0.86 (t, J = 6.5 Hz, 7H). 13C NMR (75 MHz, Chloroform-d) δ 177.35, 164.05, 162.68, 133.89, 131.59, 125.93, 125.00, 51.62, 39.31, 36.58, 33.78, 31.93, 29.70, 24.46, 22.70, 14.13. ESI-MS (positive ion mode): m/z [M]^＋^: calcd: 837.2598; obsd: 838.2653. Elemental Analysis for C_31_H_48_Cl_2_N_4_O_6_Pt C 44.39%, H 5.77%, N 6.68%; Found C 44.42%, H 5.83%, N 6.73%.

**Synthetic route of naphplatin.**

To a solution of **11a** (276 mg) in DMF (20 mL) was added a DMF solution (0.5 mL) containing TBTU (0.57 mmol). This mixture stirred for 10 min at room temperature. A DMF solution containing **4** (1.5 mmol) and DIPEA (0.92 mmol) was added to the resulting solution. The mixture was stirred at room temperature for 24 h in the dark. The DMF was then removed under vacuum to afford a yellow oil. Compound naphplatin was purified by silica gel column chromatography as a yellow solid in 57% yield. 1H NMR (300 MHz, Methanol-d4) δ 8.49 (d, J = 8.5 Hz, 1H), 8.27 (d, J = 8.4, 2.8 Hz, 1H), 7.62 (d, J = 7.9 Hz, 1H), 6.86 (d, J = 8.5 Hz, 1H), 2.59 (t, 2H), 2.29 (t, J = 28.0, 7.5 Hz, 2H), 1.57 (dt, J = 7.3 Hz, 2H), 1.28 (s, 26H), 0.88 (t, J = 6.9 Hz, 3H).^13^C NMR (75 MHz, CDCl_3_) δ = 182.08, 173.24, 156.65, 156.21, 155.48, 54.39, 32.20, 31.96, 31.87, 29.68, 28.71, 28.45, 27.73, 22.64, 18.01, 14.08, 8.02. ESI-MS (positive ion mode): m/z [M]^＋^: calcd: 837.2598; obsd: 838.2653. Elemental Analysis for C_31_H_48_Cl_2_N_4_O_6_Pt C 44.39%, H 5.77%, N 6.68%; Found C 44.42%, H 5.83%, N 6.73%.

**Results and Discussion**

**Figure S1.** Chemical structures of amonafide, mitonafide, cisplatin and oxaliplatin in clinic, key intermediates **5a-5c** and **11a-11d**, **7a-7c**, **12a-12d** and **14a-14b**, Pt(IV) prodrugs without naphthalimide modified **6**, **13**, **1a** and **2a**.


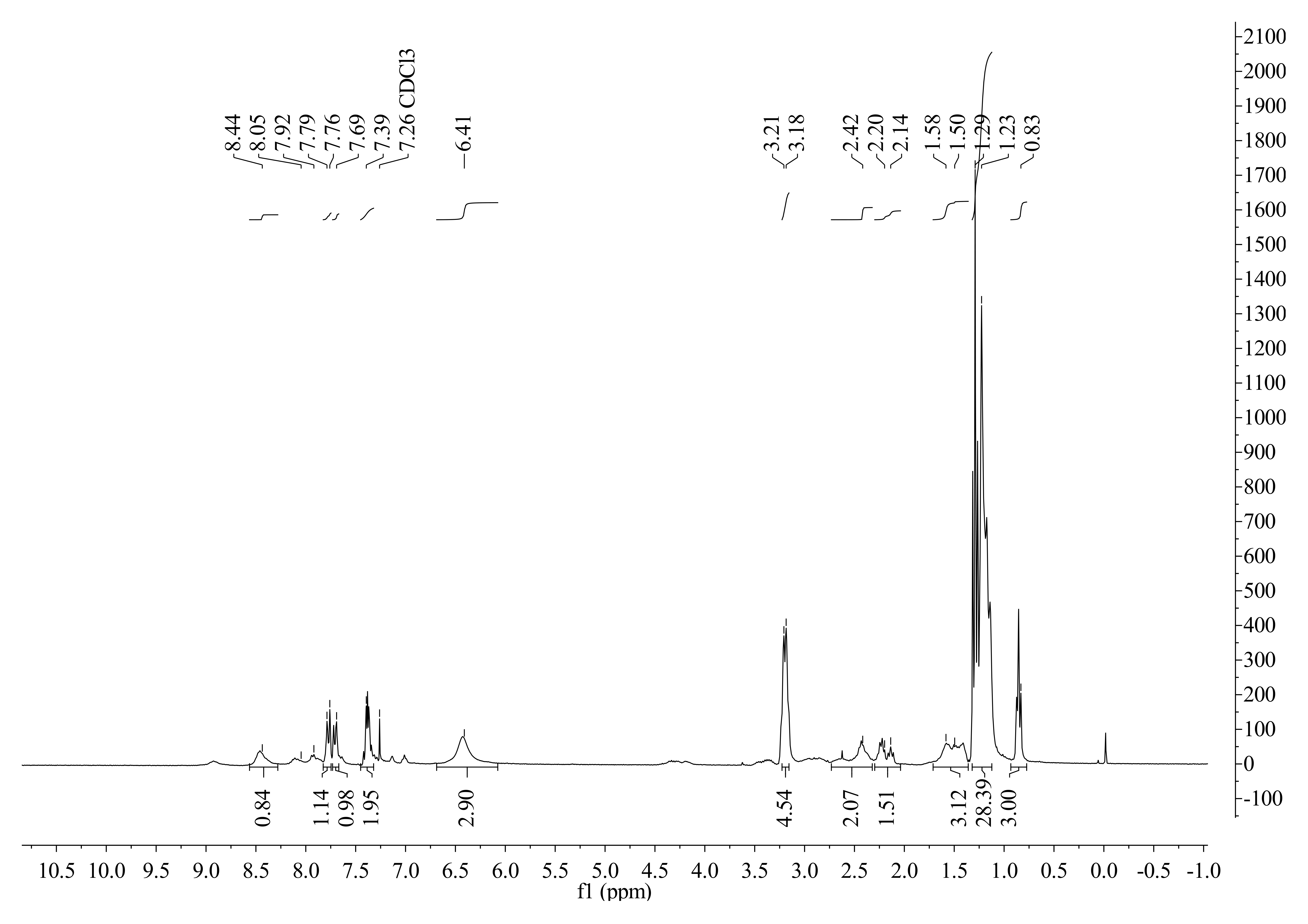

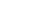

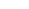


**Figure S2.** ^1^H NMR spectrum of compound **7a**.


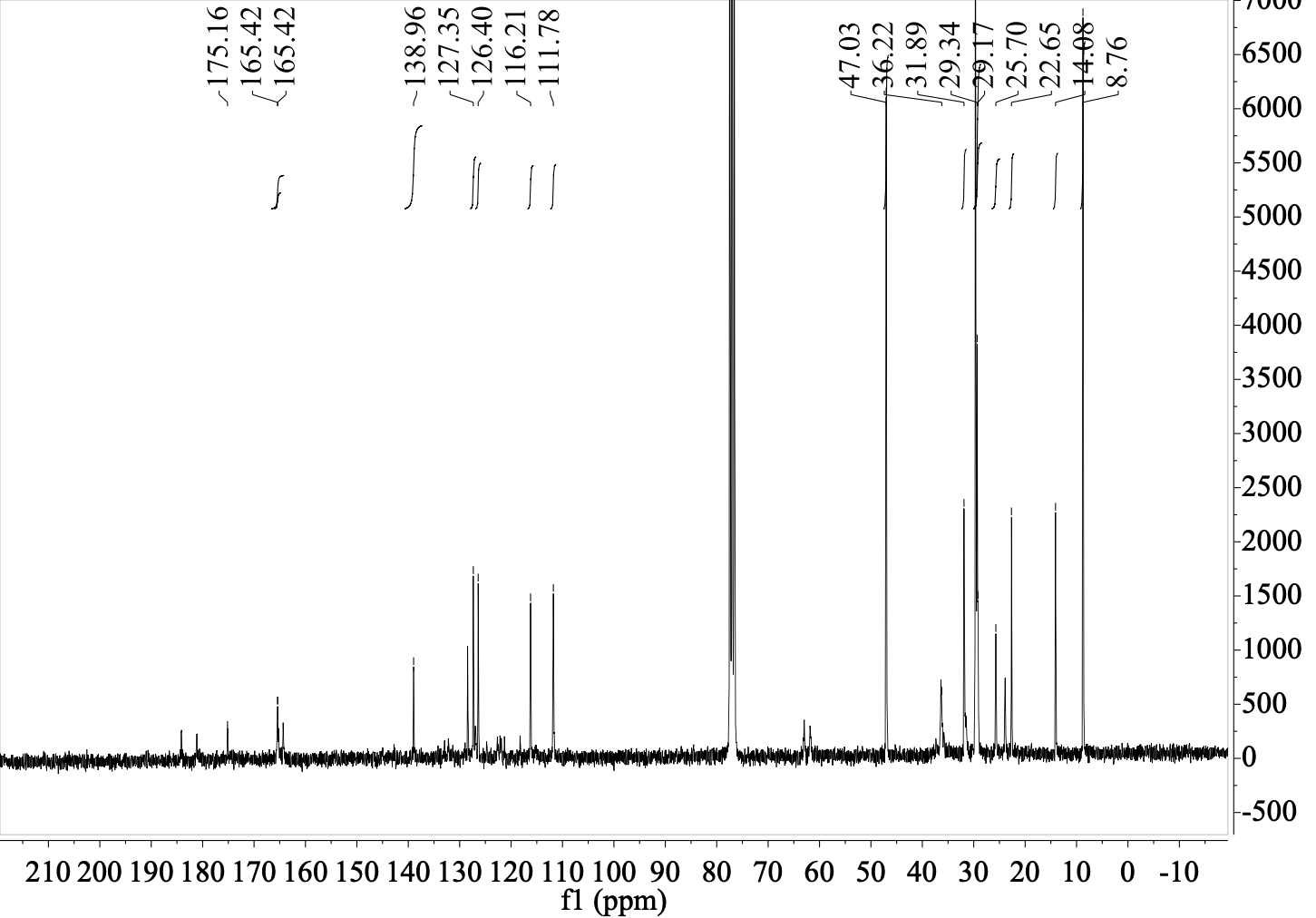

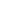

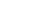

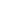


**Figure S3.** ^13^C NMR spectrum of compound **7a**.


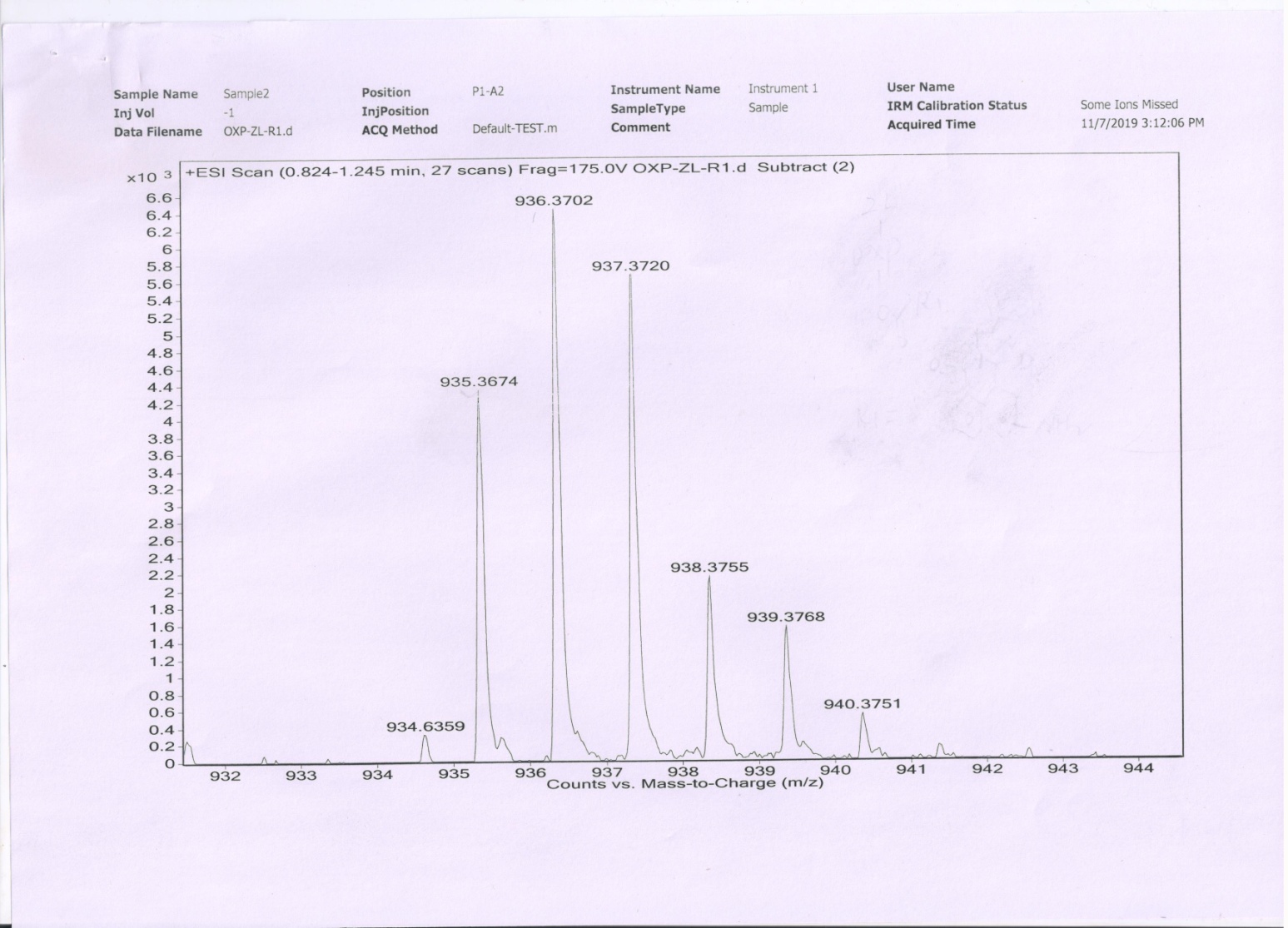


**Figure S4.** ESI mass spectrum of compound **7a.**


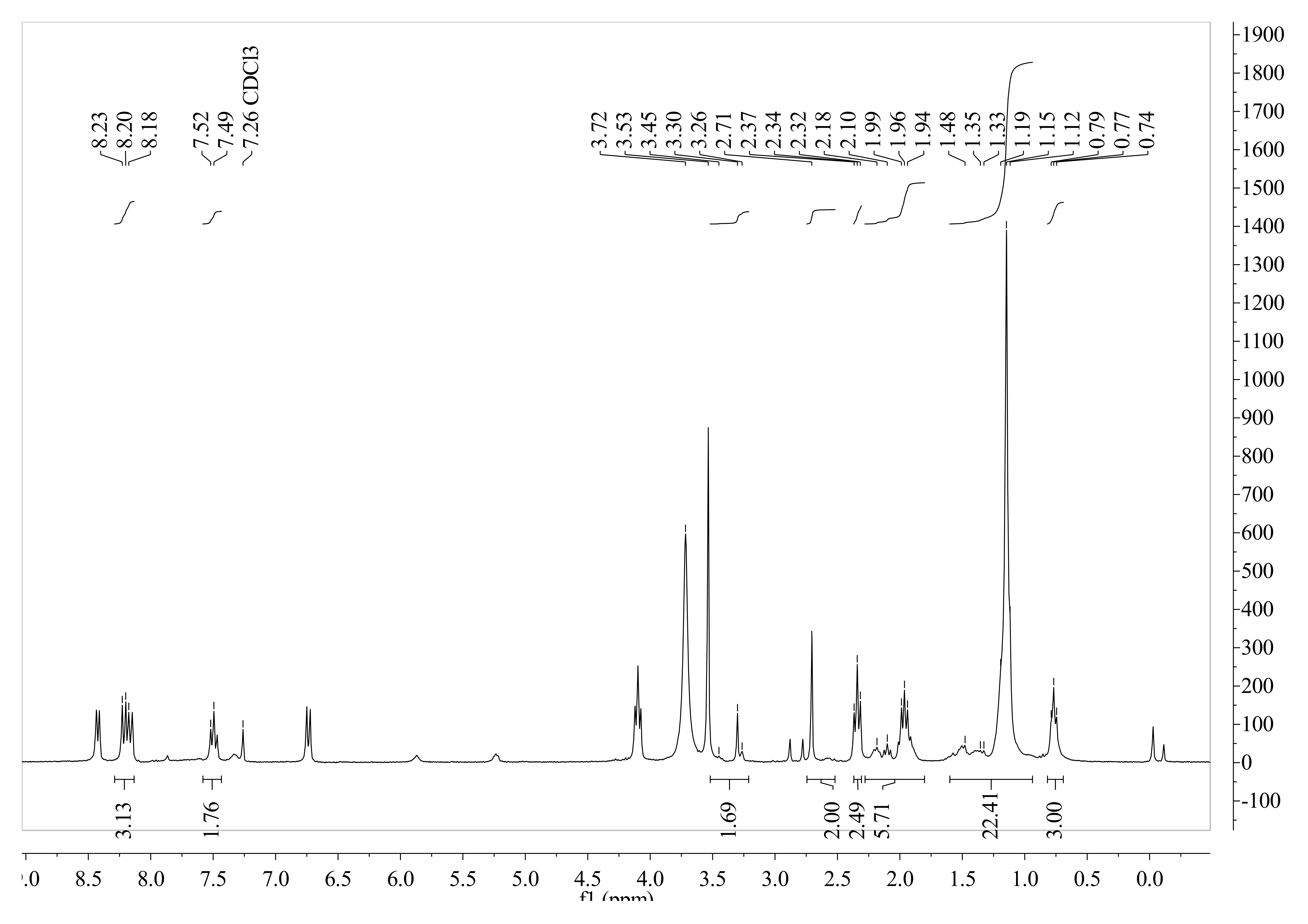


**Figure S5.** ^1^H NMR spectrum of compound **12a**.


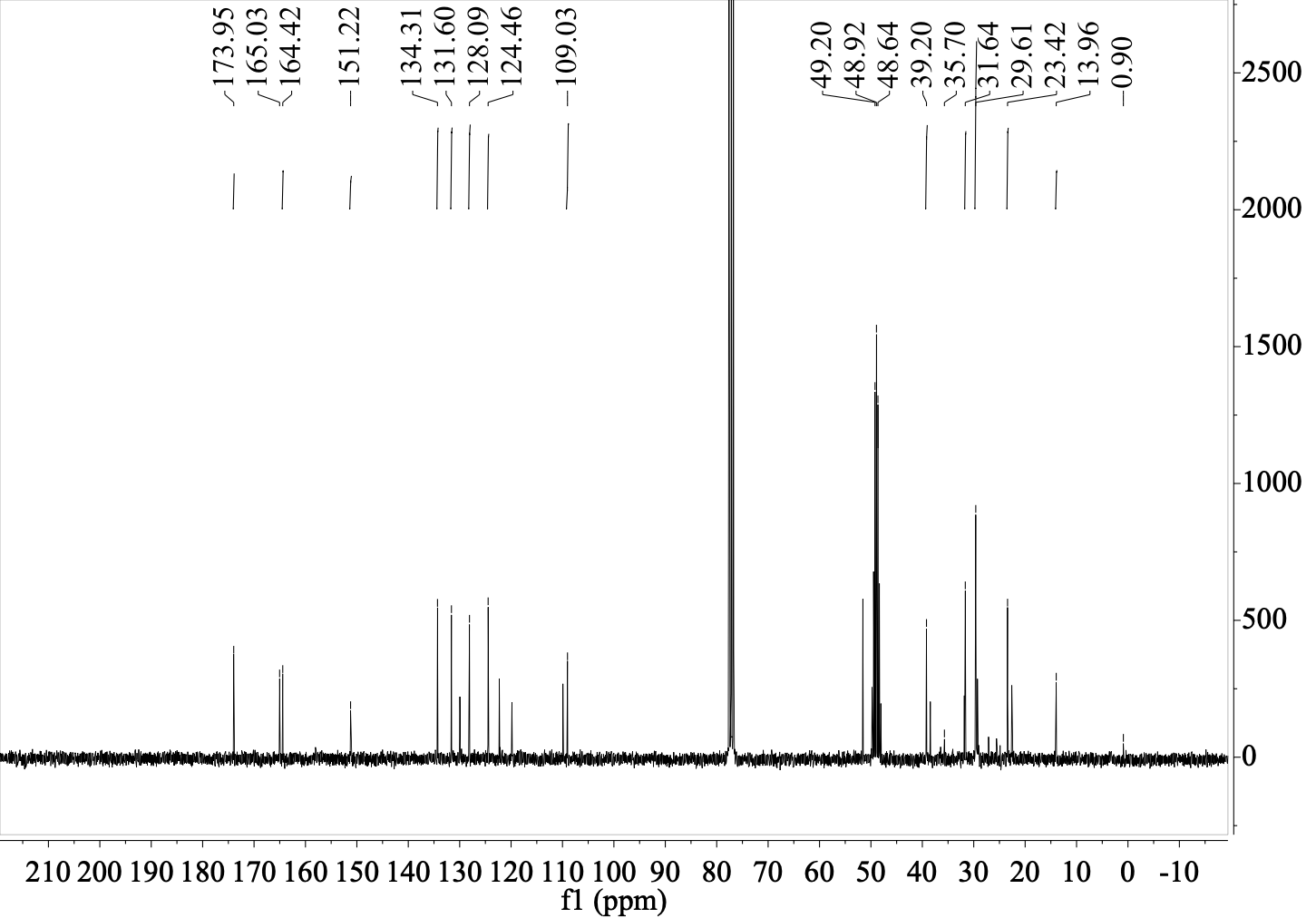


**Figure S6.** ^13^C NMR spectrum of compound **12a**.

**
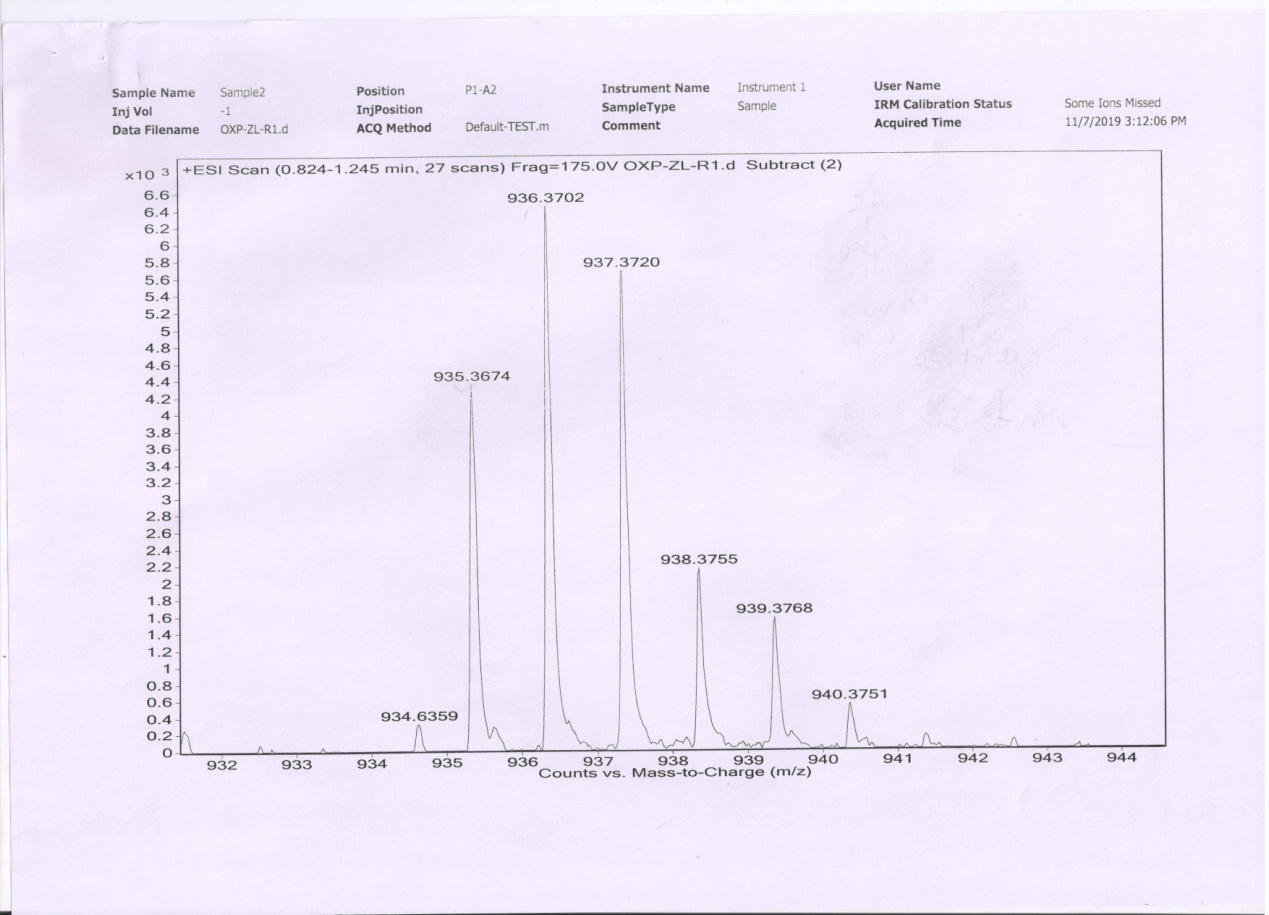
**

**Figure S7.** ESI mass spectrum of compound **12a.**

**
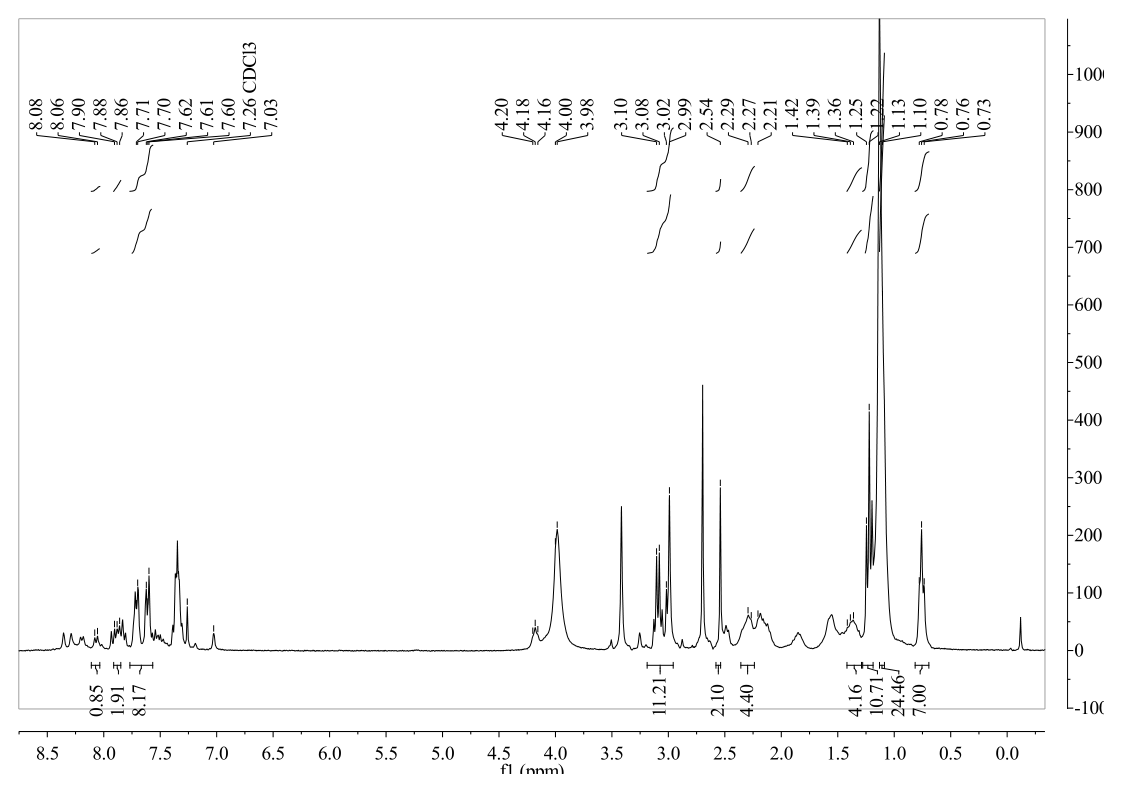
**

**Figure S8.** ^1^H NMR spectrum of compound **7b**.


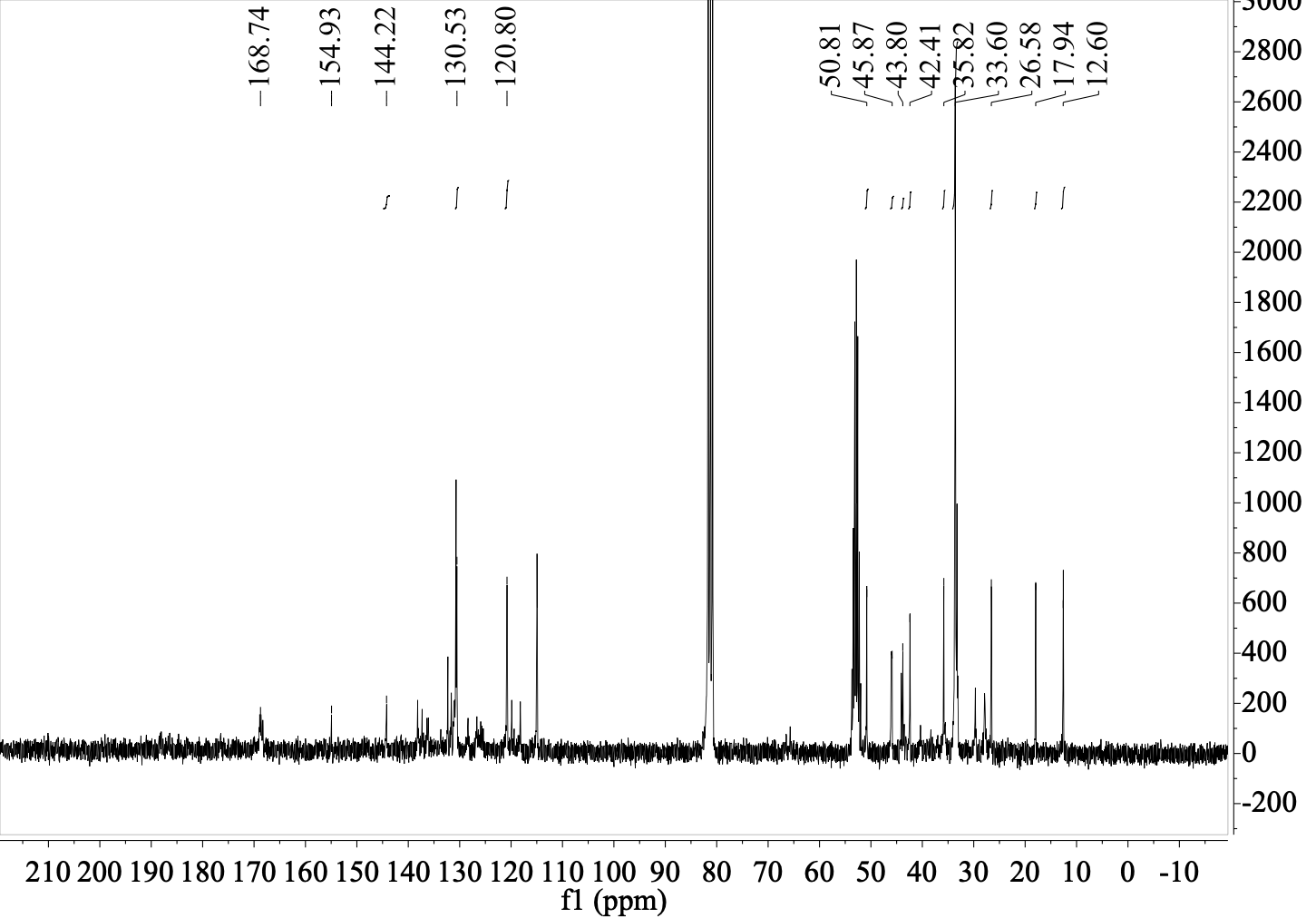


**Figure S9.** ^13^C NMR spectrum of compound **7b**.

**
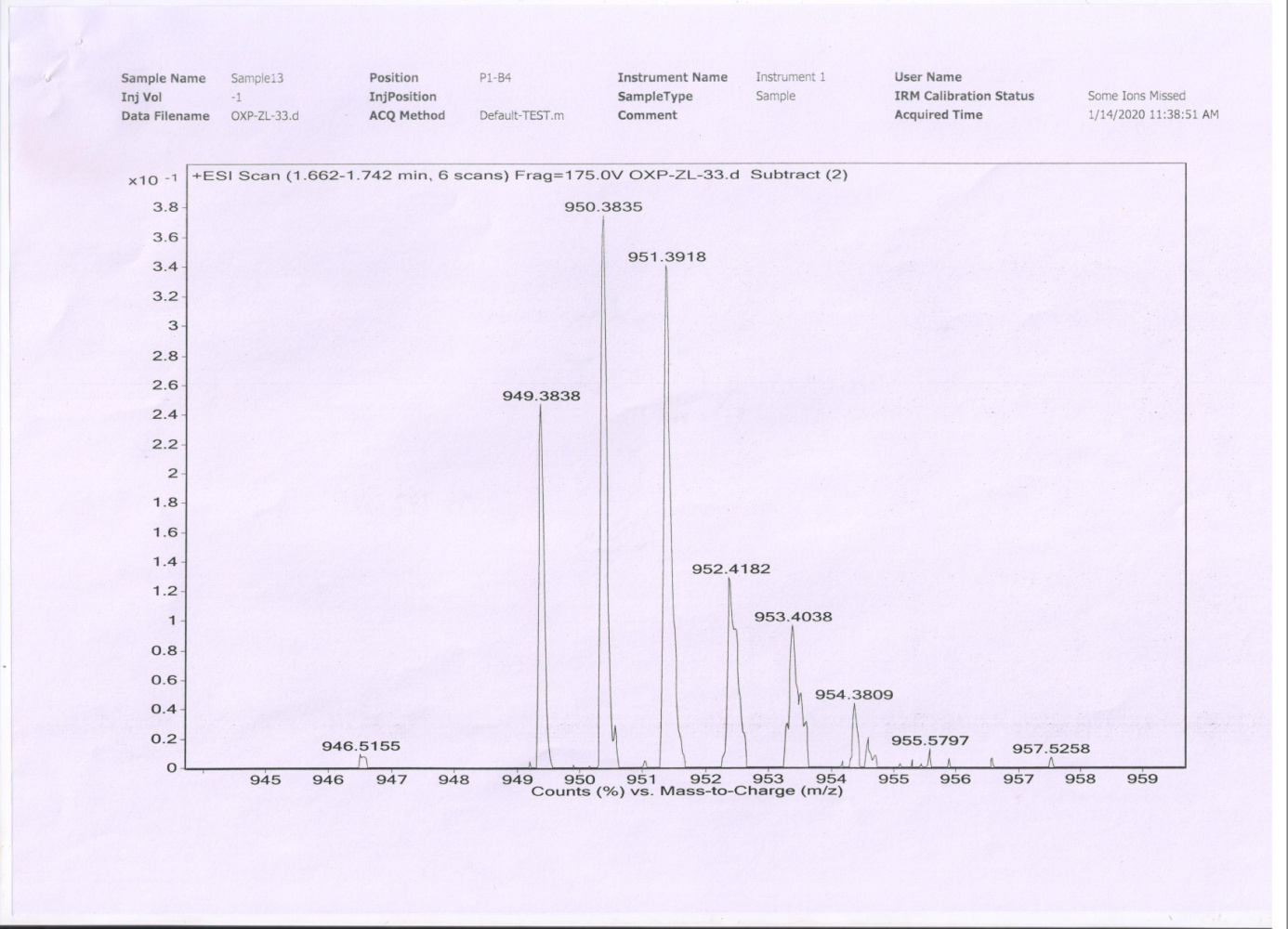
**

**Figure S10.** ESI mass spectrum of compound **7b.**

**
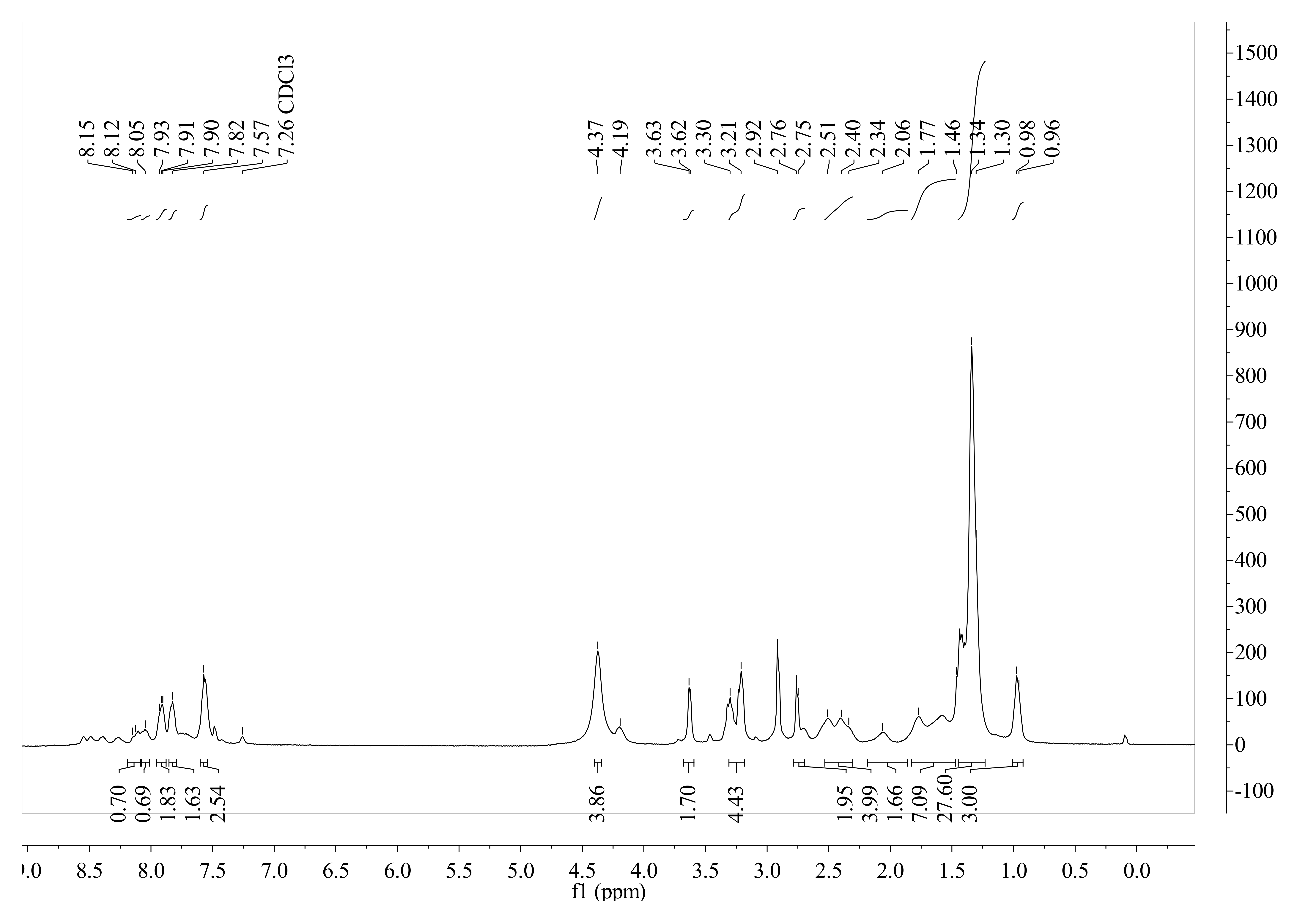
**

**Figure S11.** ^1^H NMR spectrum of compound **12b**.


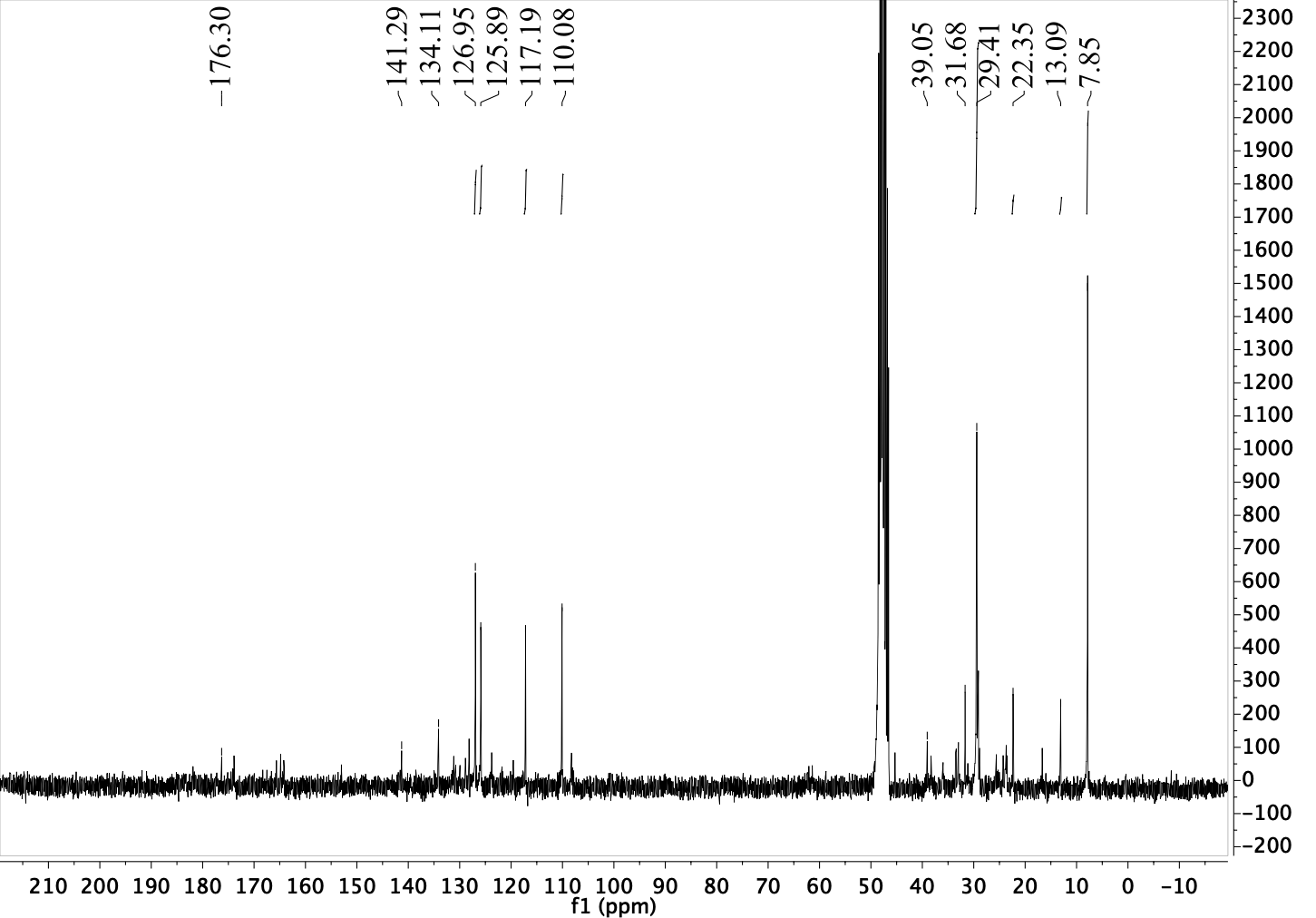


**Figure S12.** ^13^C NMR spectrum of compound **12b**.

**
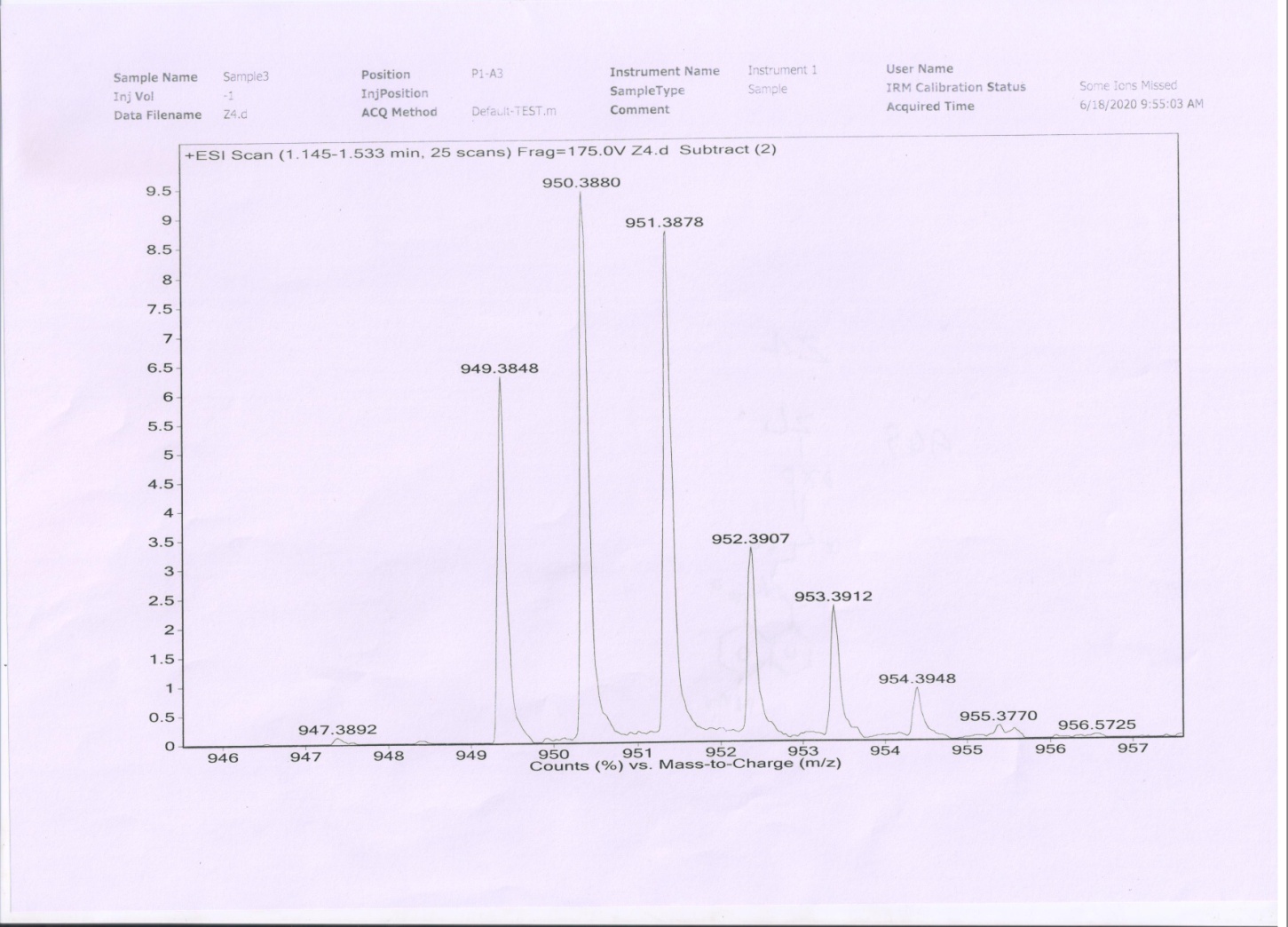
**

**Figure S13.** ESI mass spectrum of compound **12b.**


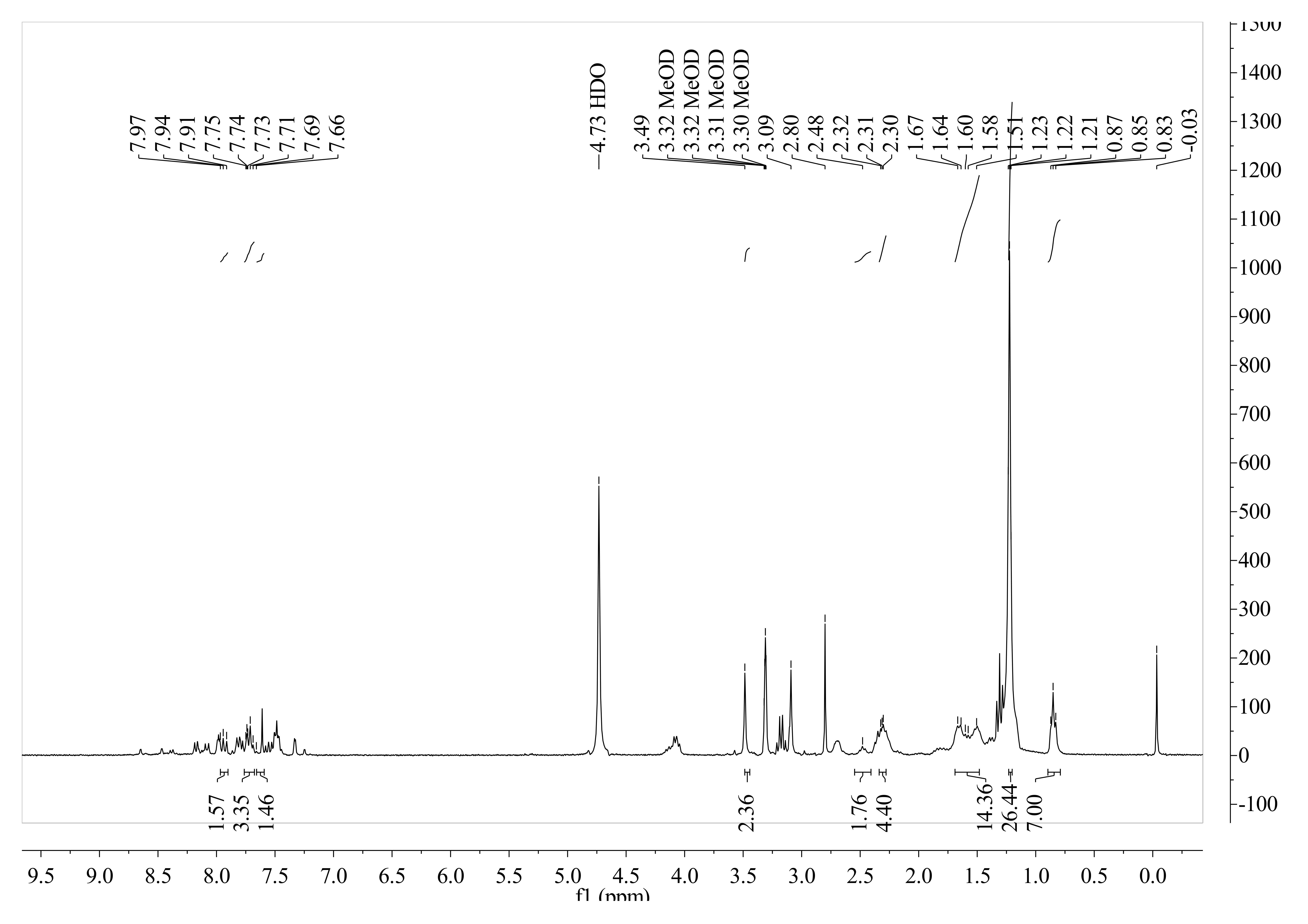


**Figure S14.** ^1^H NMR spectrum of compound **7c**.


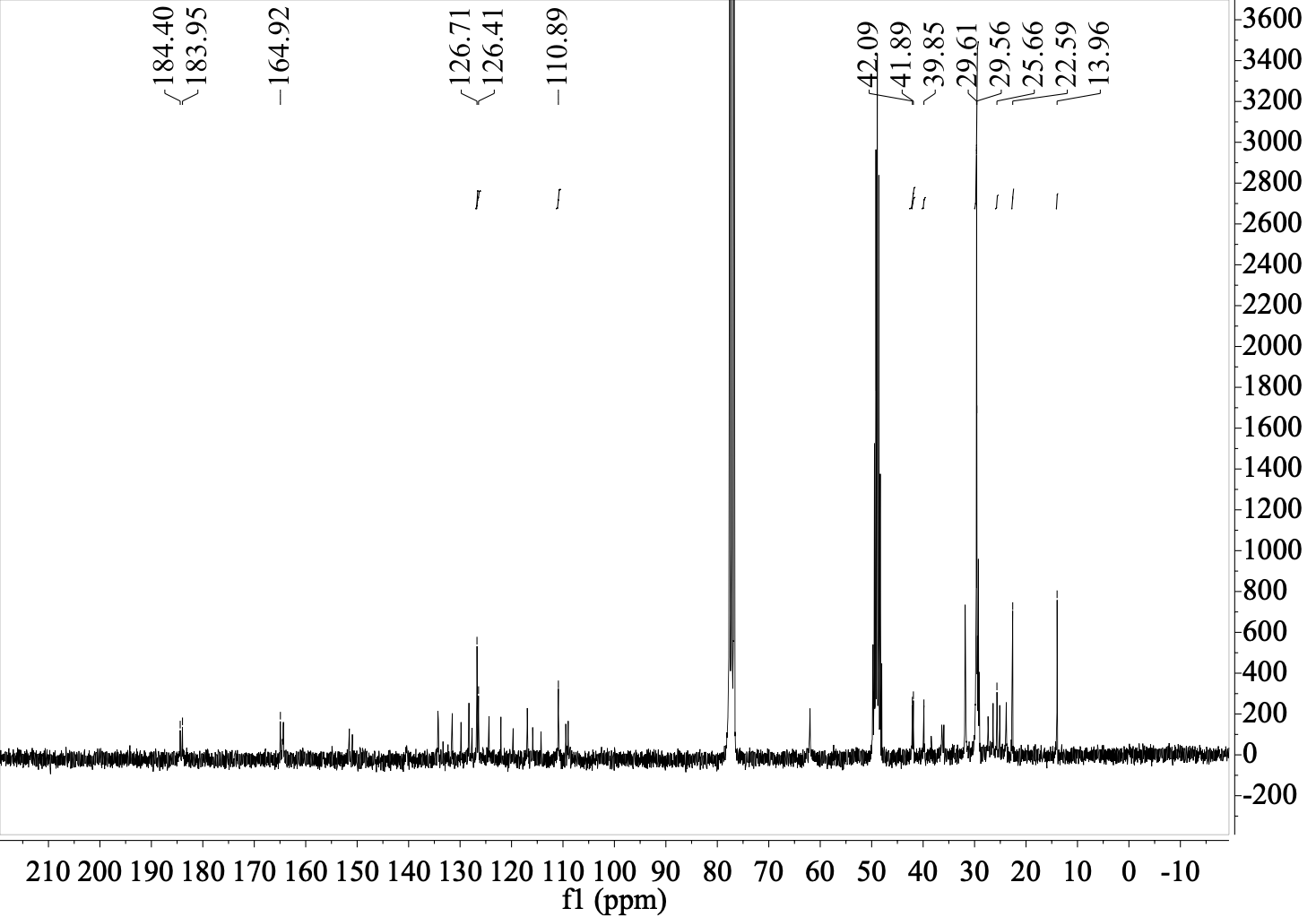


**Figure S15.** ^13^C NMR spectrum of compound **7c**.

**
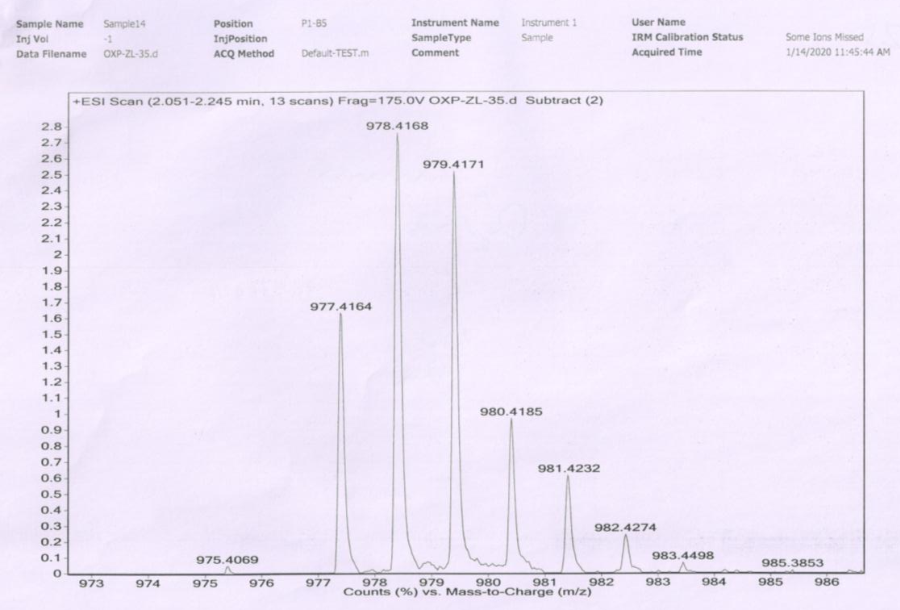
**

**Figure S16.** ESI mass spectrum of compound **7c.**

**
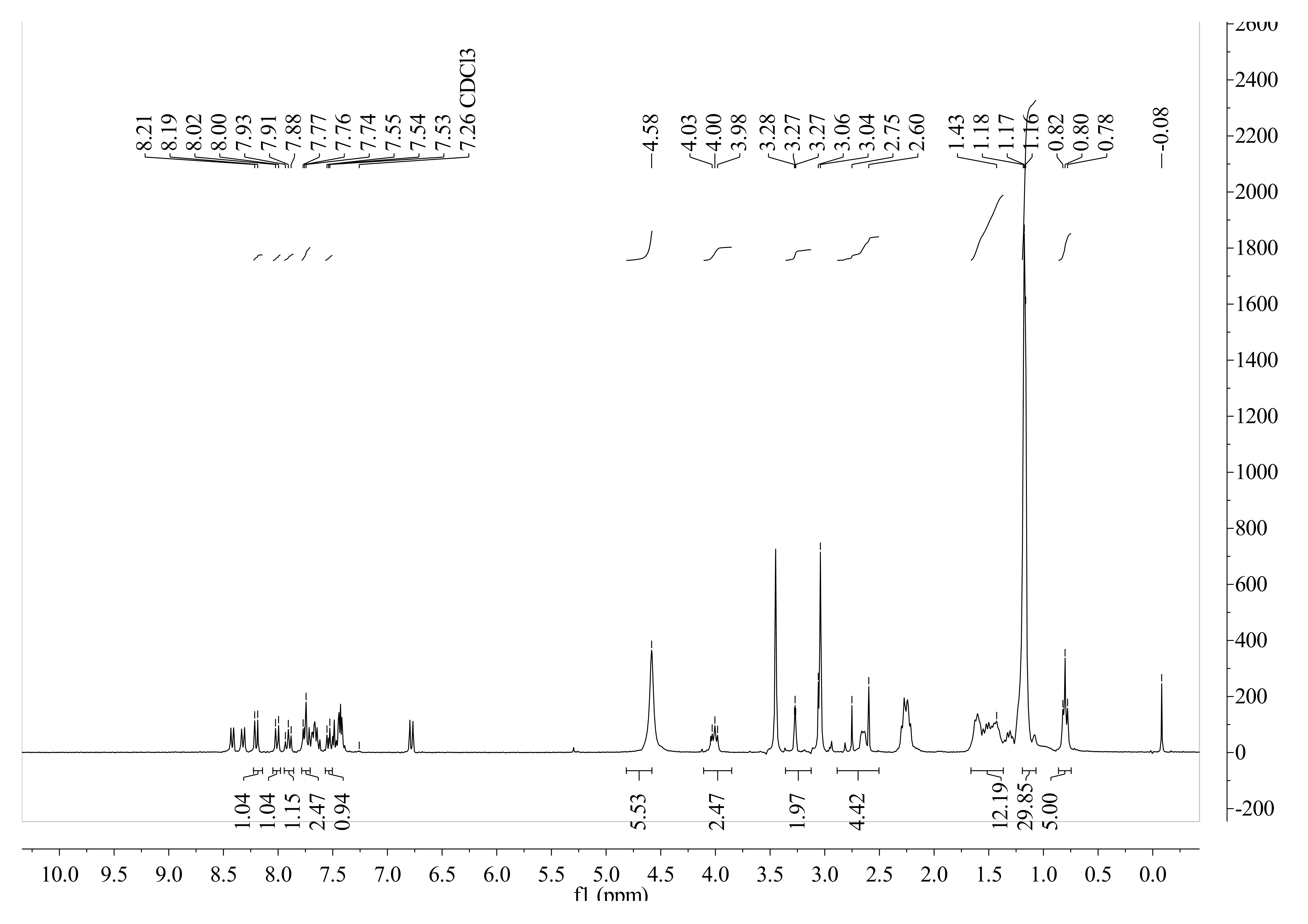
**

**Figure S17.** ^1^H NMR spectrum of compound **12c**.


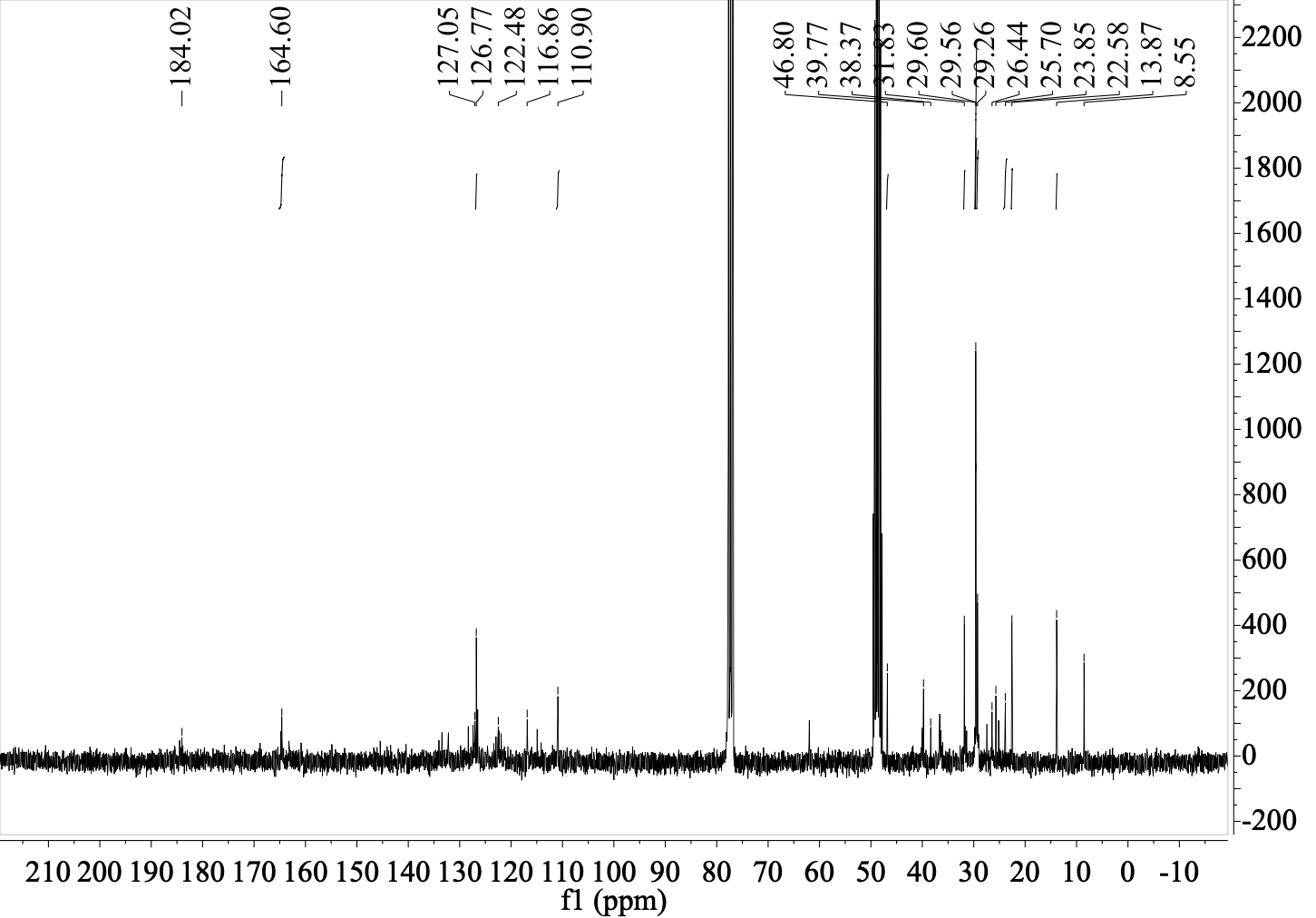


**Figure S18.** ^13^C NMR spectrum of compound **12c**.

**
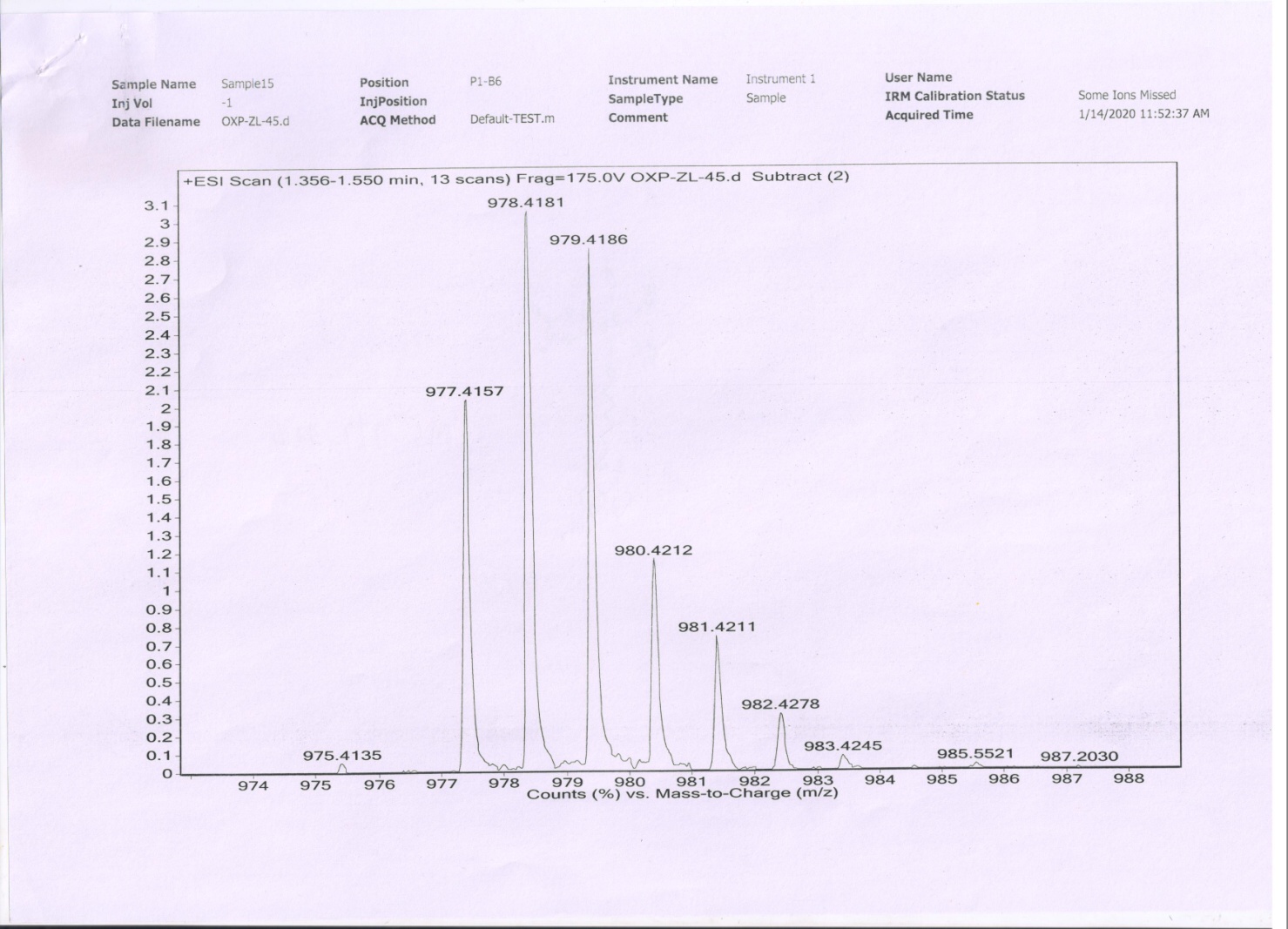
**

**Figure S19.** ESI mass spectrum of compound **12c.**

**
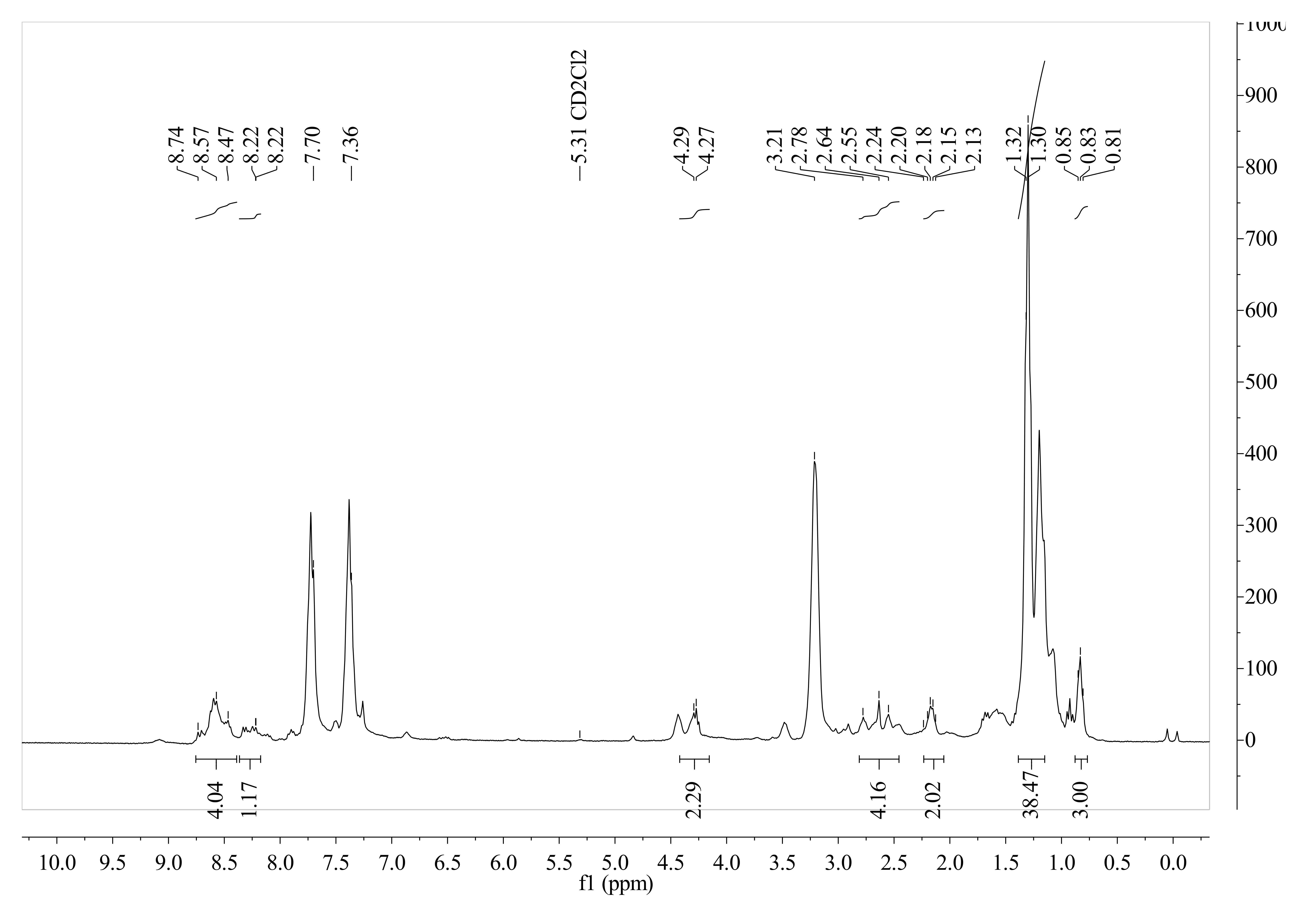
**

**Figure S20.** ^1^H NMR spectrum of compound **12d**.
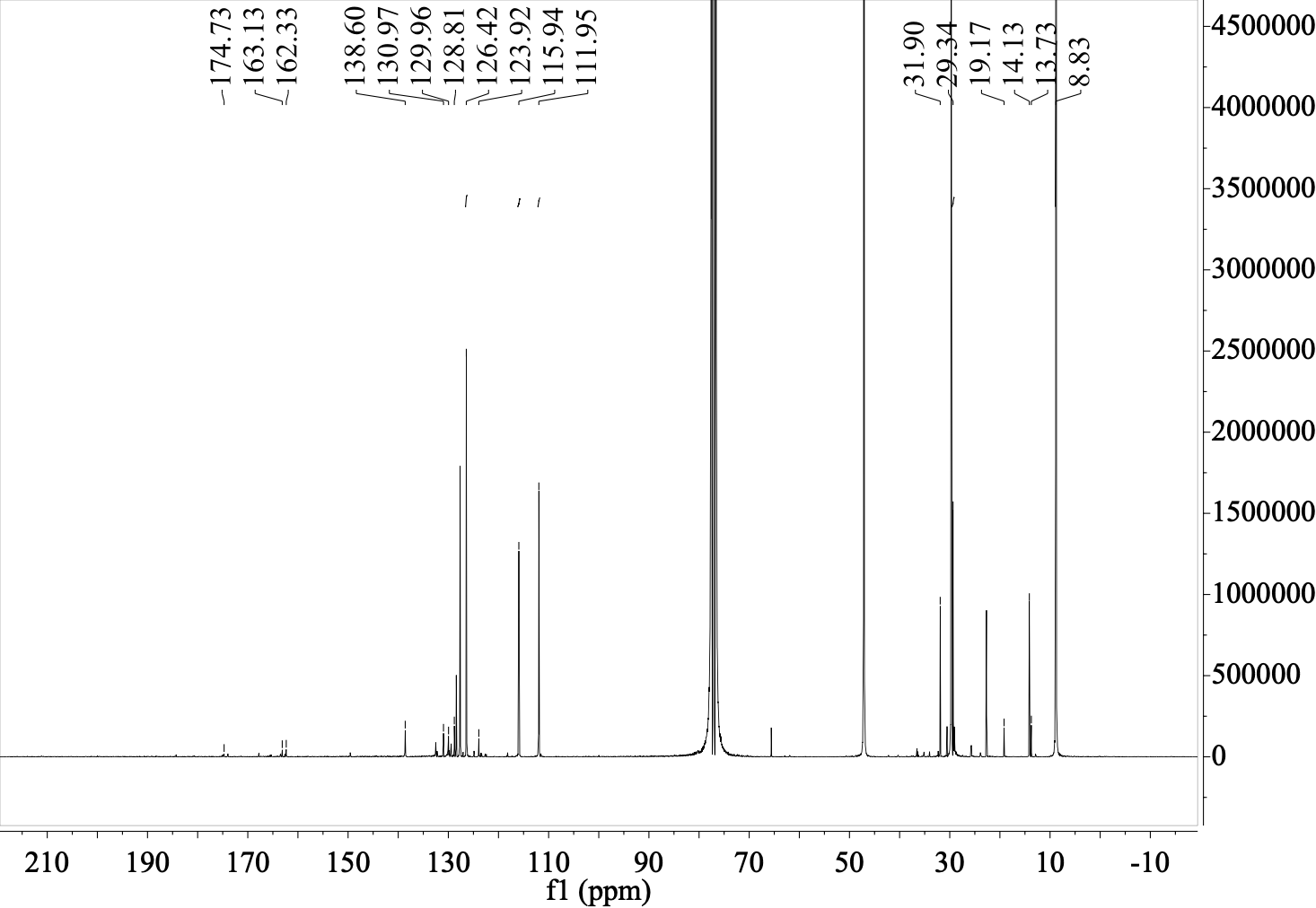


**Figure S21.** ^13^C NMR spectrum of compound **12d.**

**
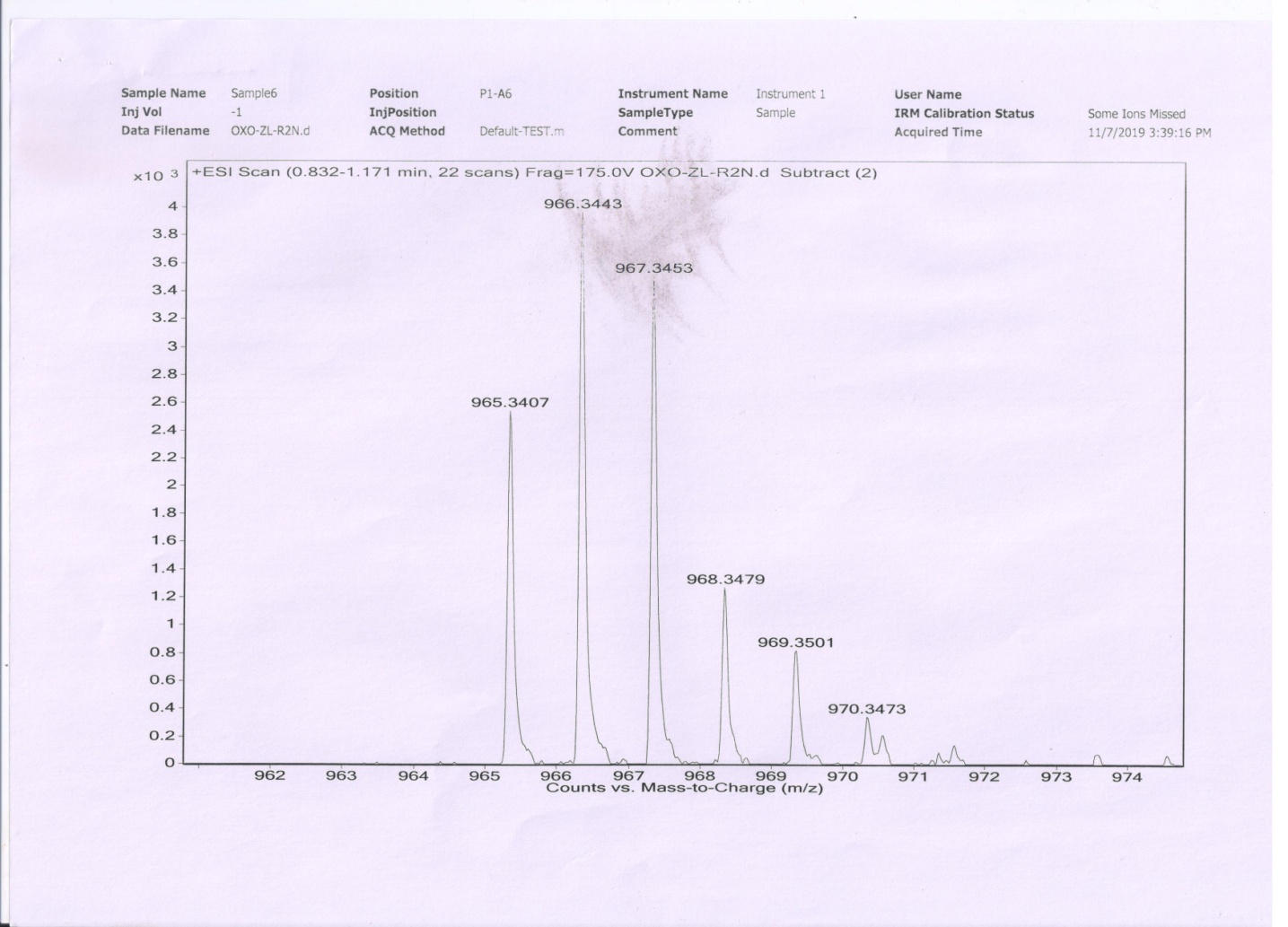
**

**Figure S22.** ESI mass spectrum of compound **12d.**


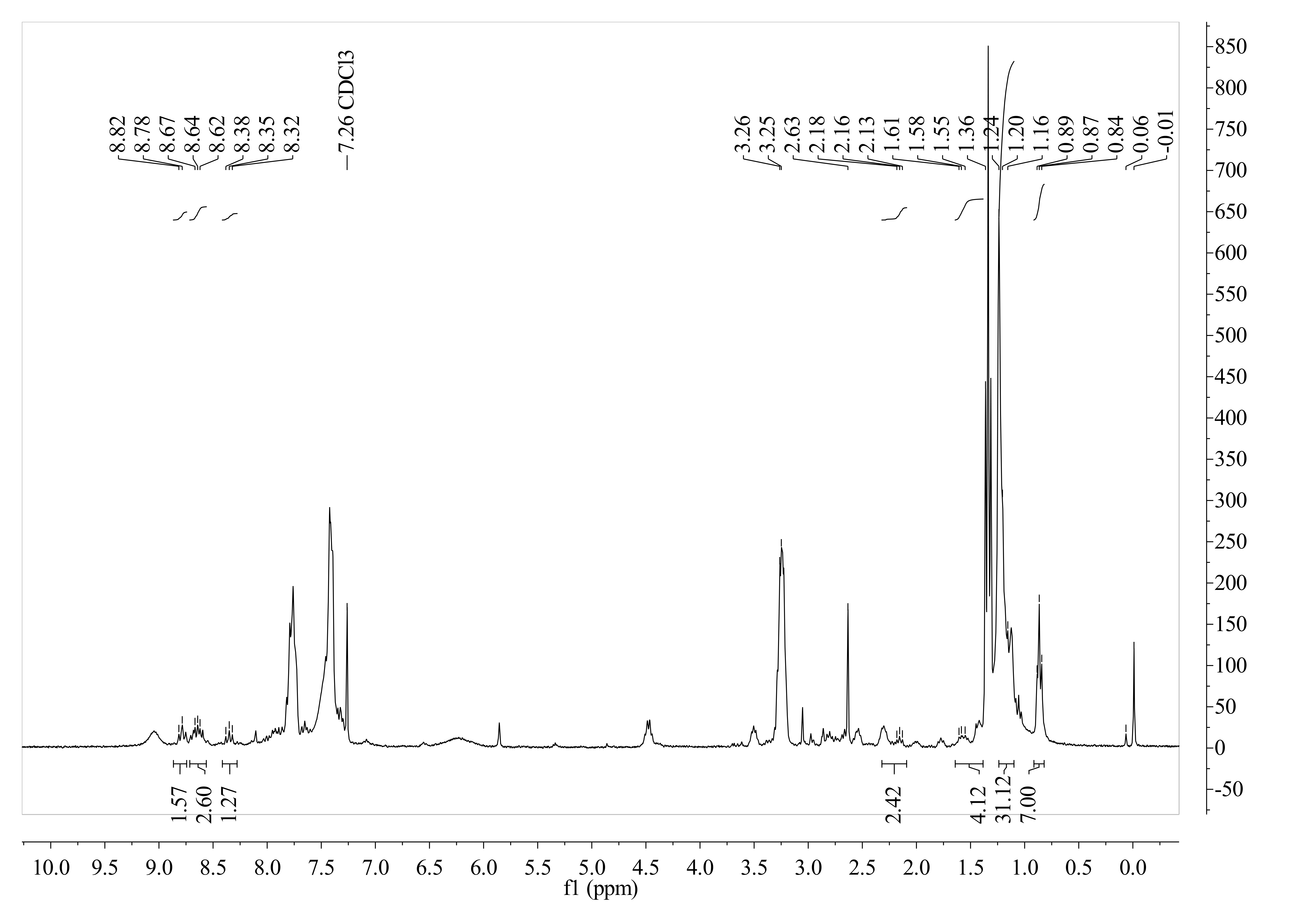


**Figure S23.** ^1^H NMR spectrum of compound **14a**.
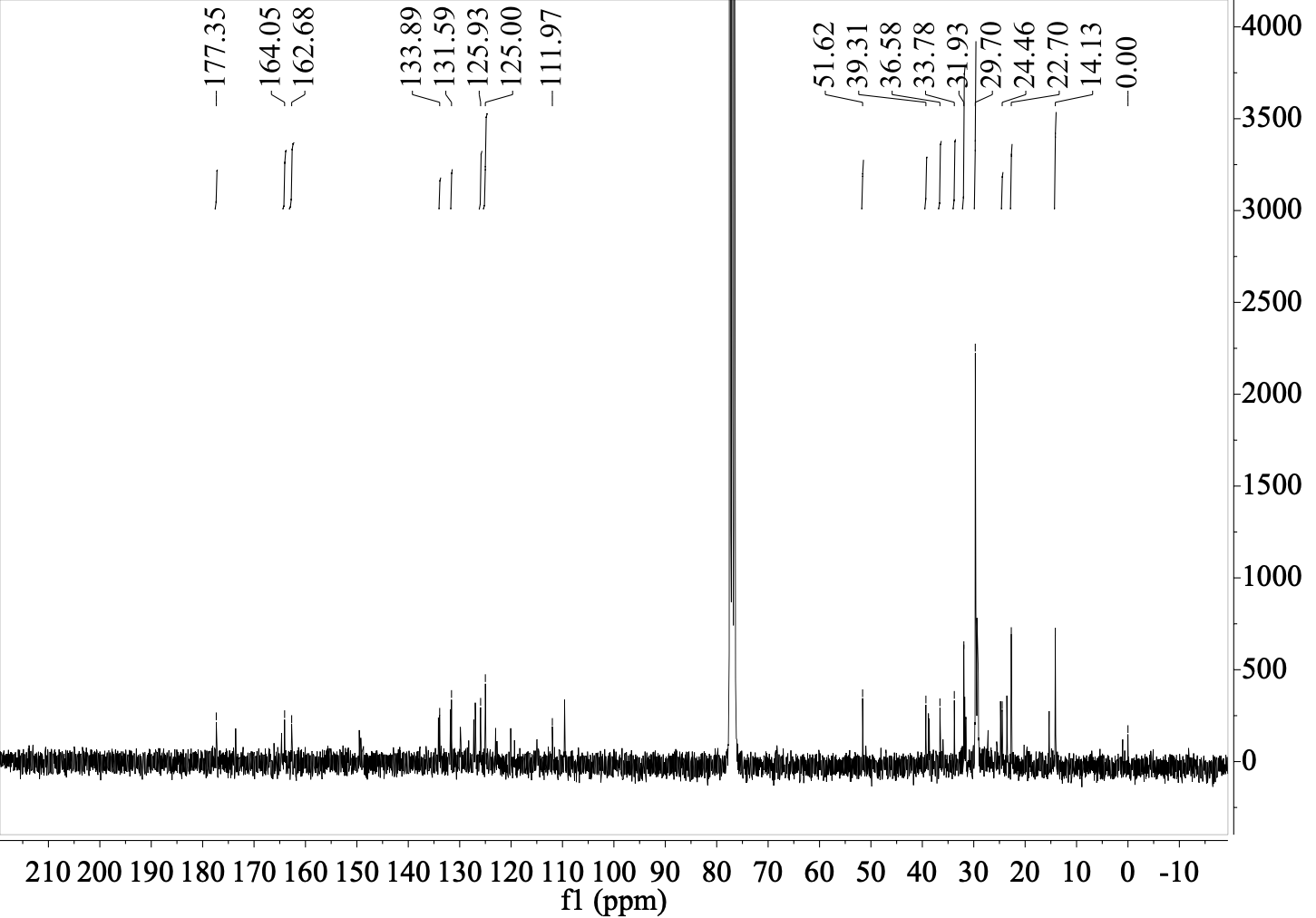


**Figure S24.** ^13^C NMR spectrum of compound **14a.**

**
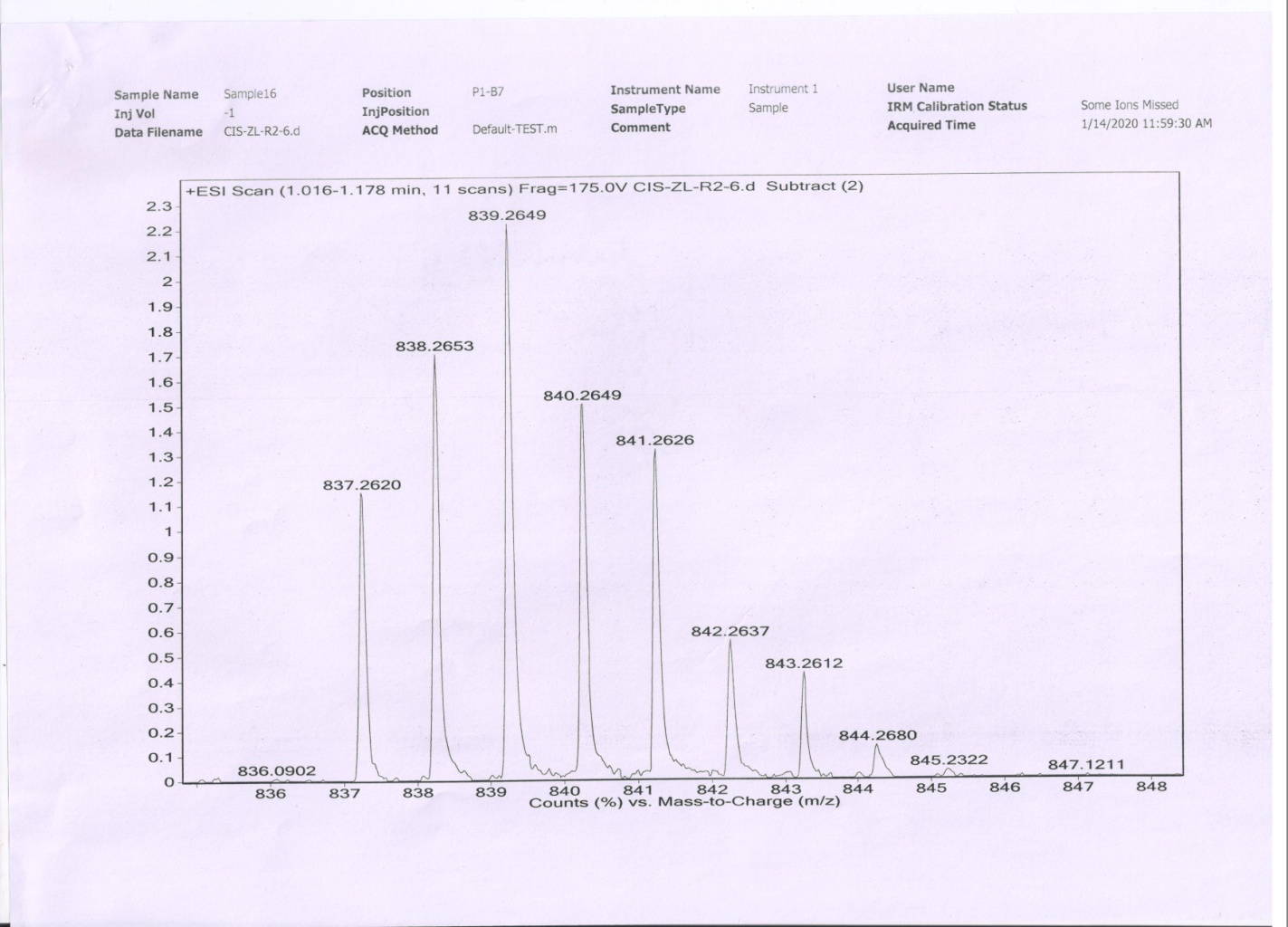
**

**Figure S25.** ESI mass spectrum of compound **14a.**

**
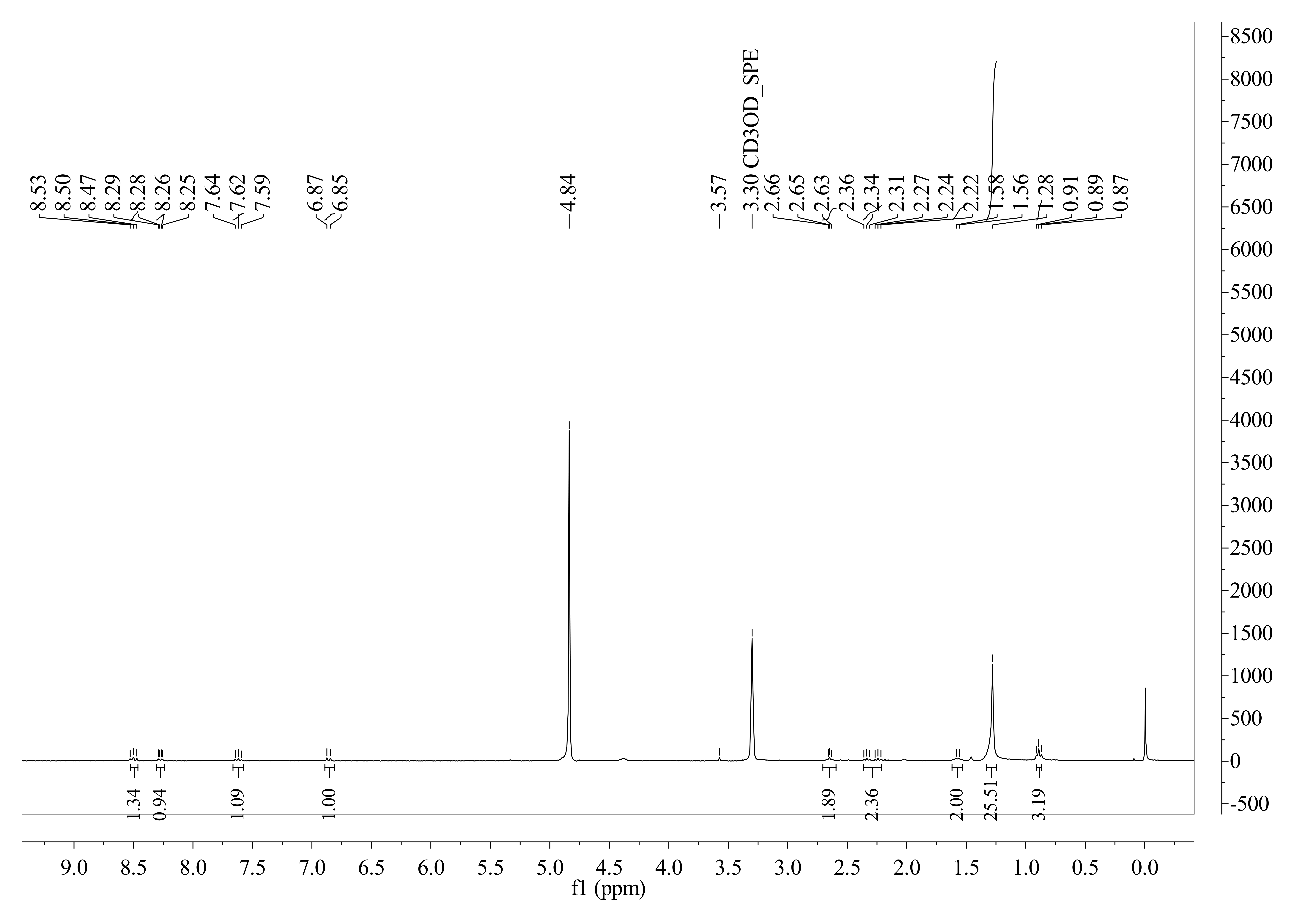
**

**Figure S26.** ^1^H NMR spectrum of compound **naphplatin**.

**
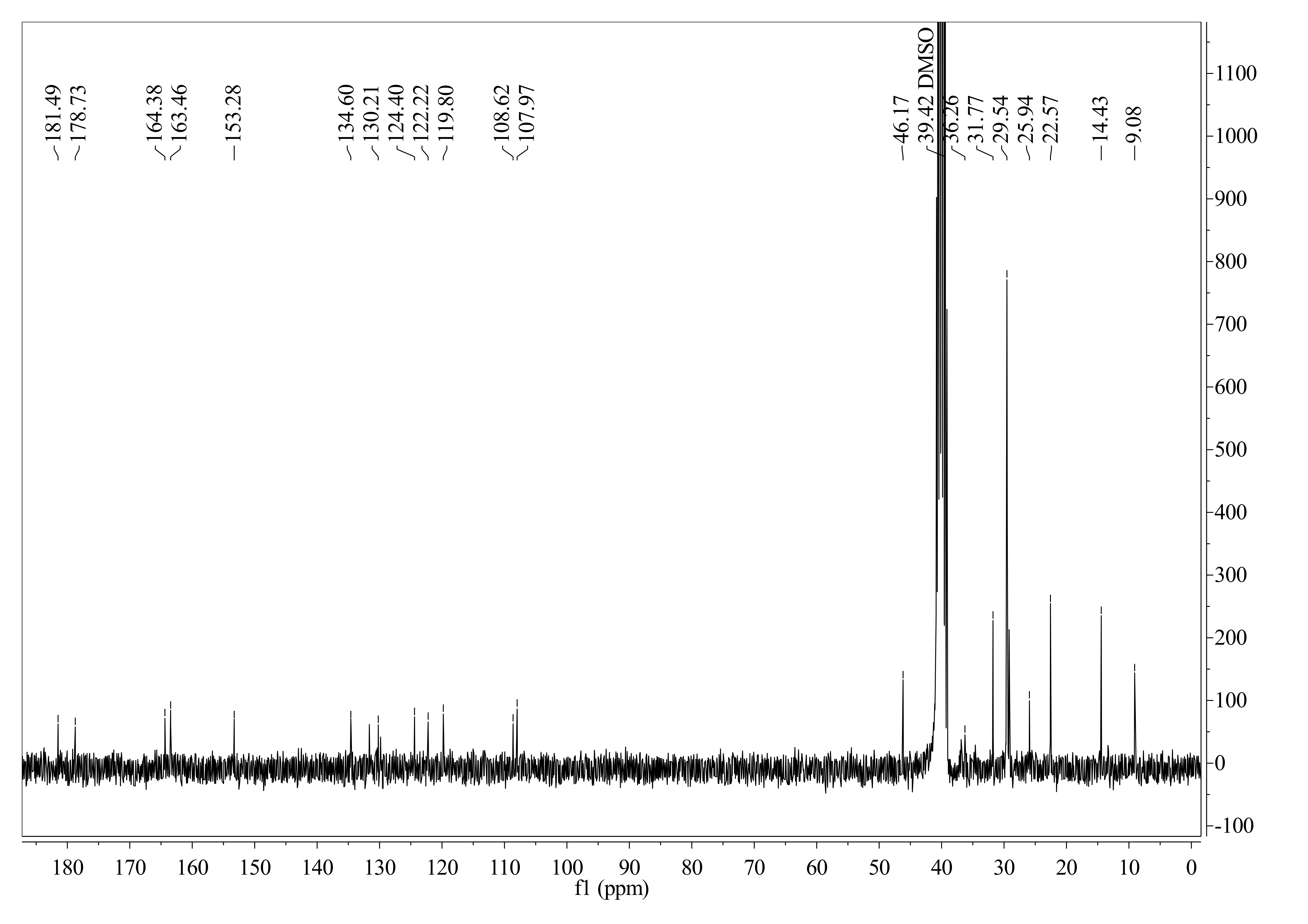
**

**Figure S27.** ^13^C NMR spectrum of compound **naphplatin.**

**
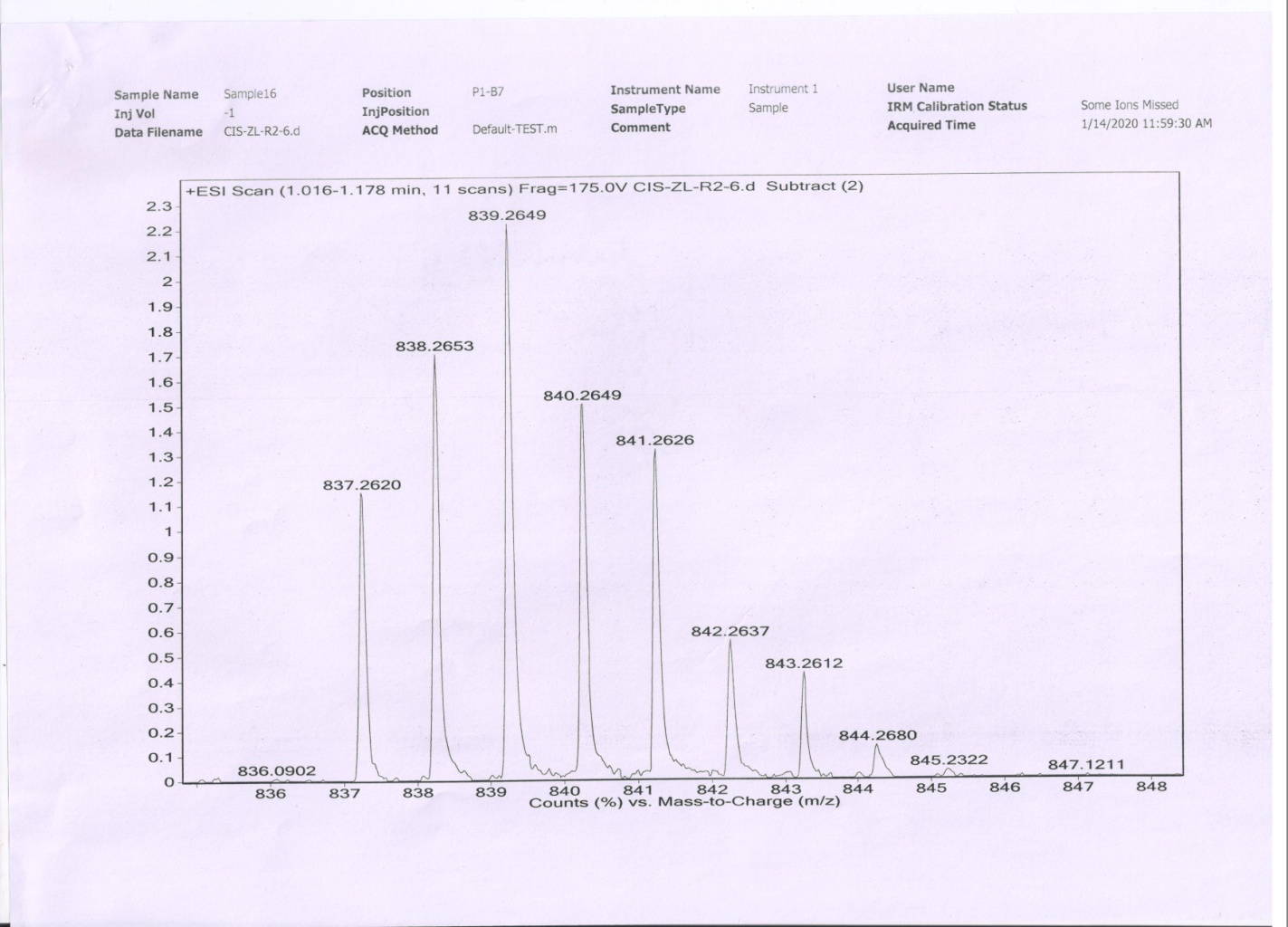
**

**Figure S28.** ESI mass spectrum of compound **naphplatin.**


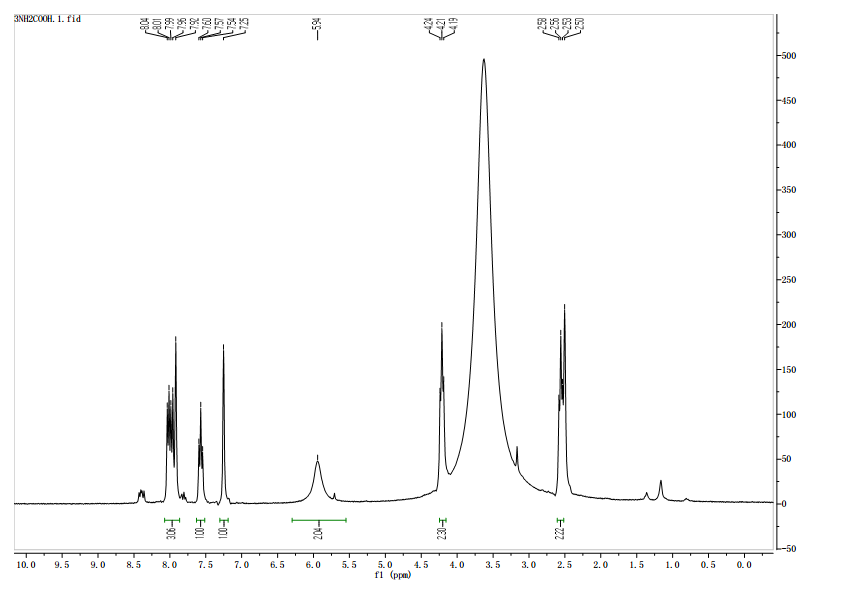


**Figure S29.** ^1^HNMR spectrum of compound **5a**.


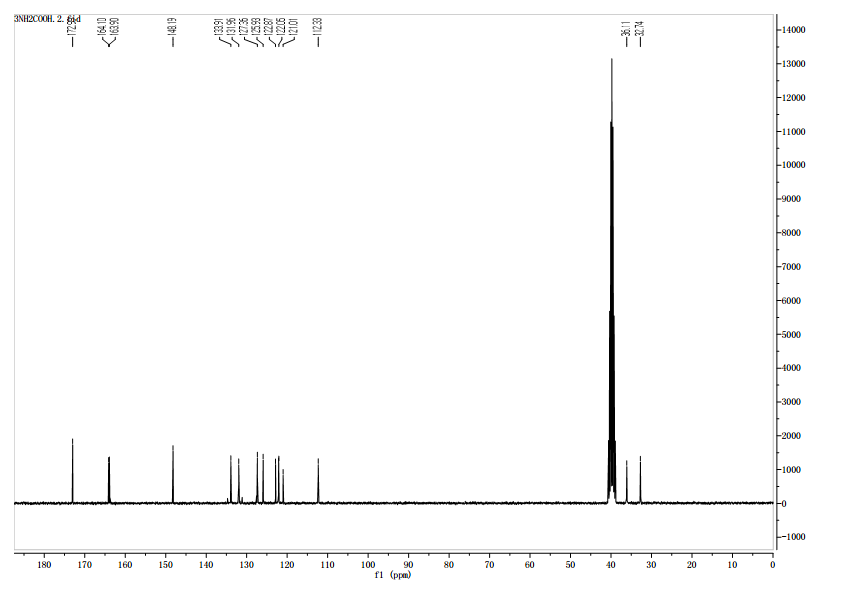


**Figure S30.** ^13^CNMR spectrum of compound **5a**.


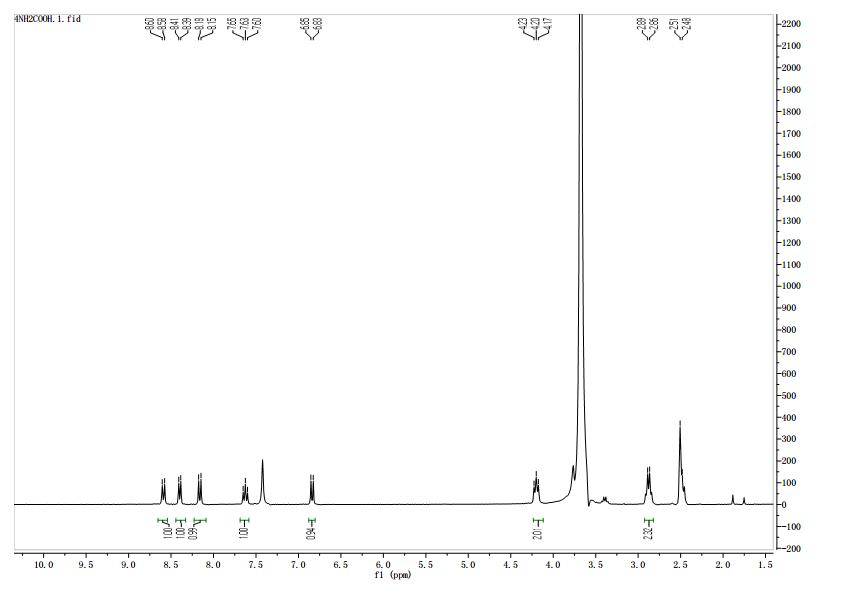


**Figure S31.**^1^HNMR spectrum of compound **11a**.


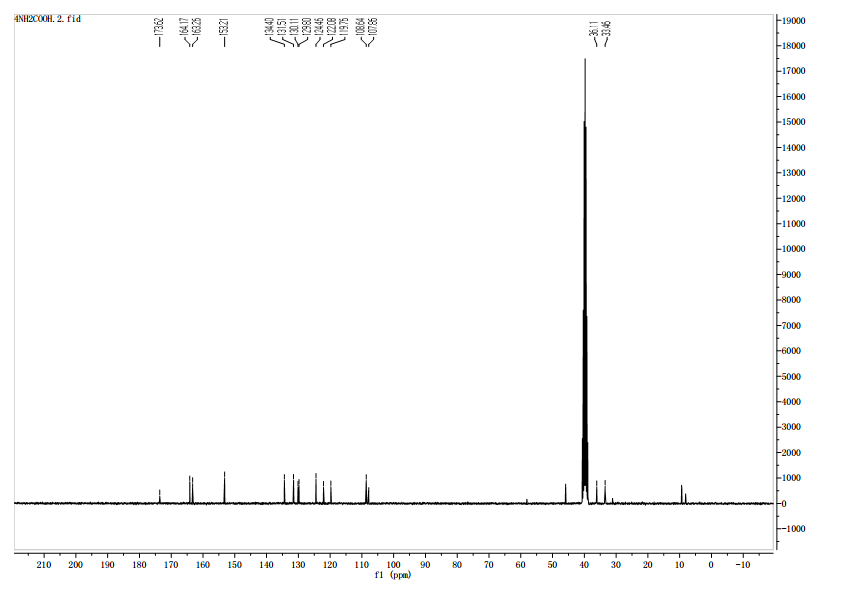


**Figure S32.**^13^CNMR spectrum of compound **11a**.


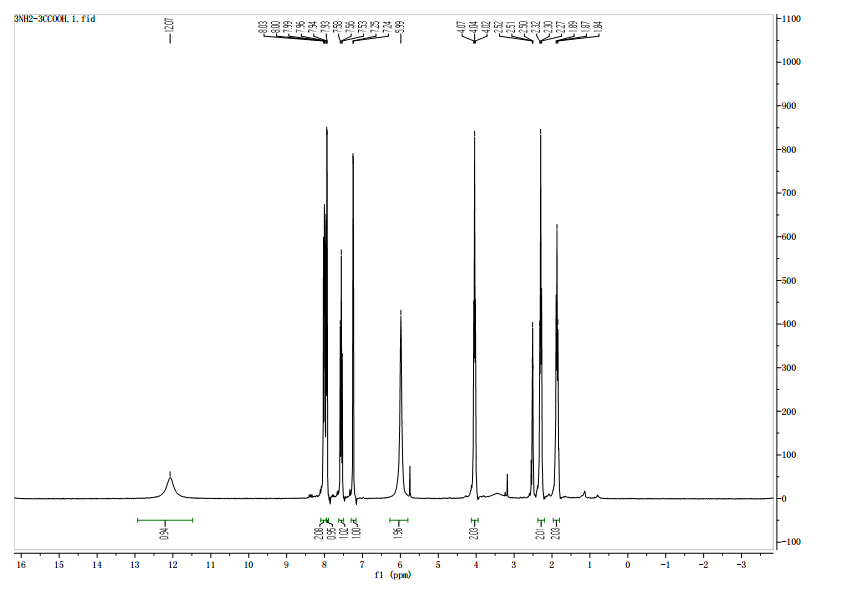


**Figure S33.**^1^HNMR spectrum of compound **5b**.


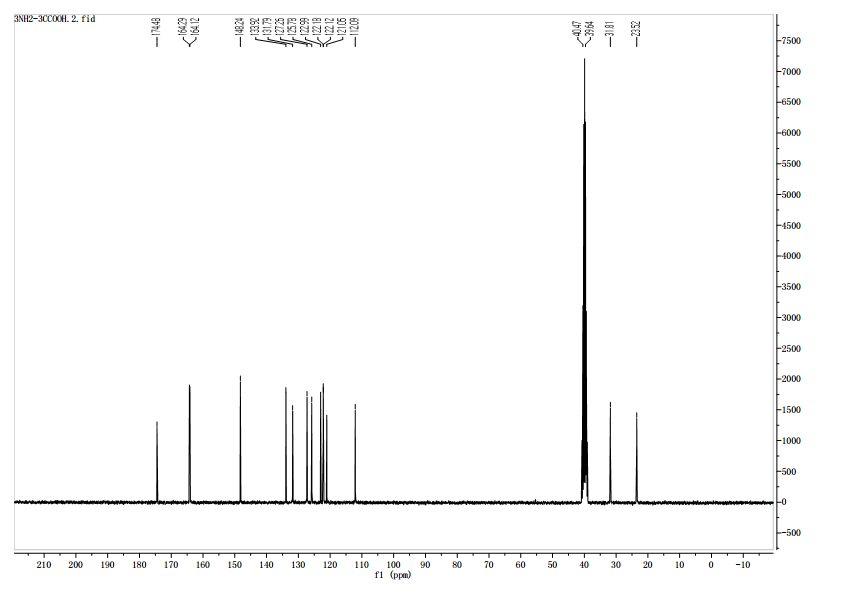


**Figure S34.** ^13^CNMR spectrum of compound **5b**.


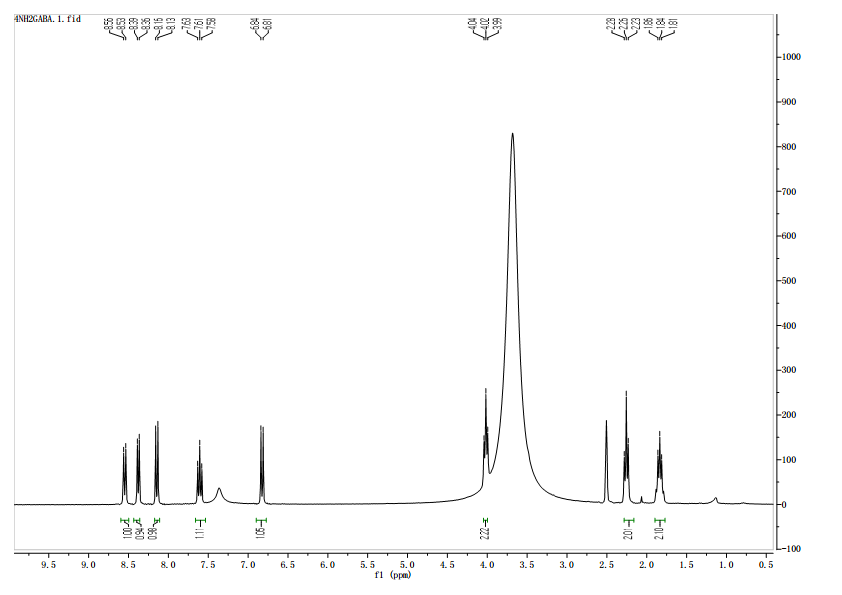


**Figure S35.** ^1^HNMR spectrum of compound **11b**.


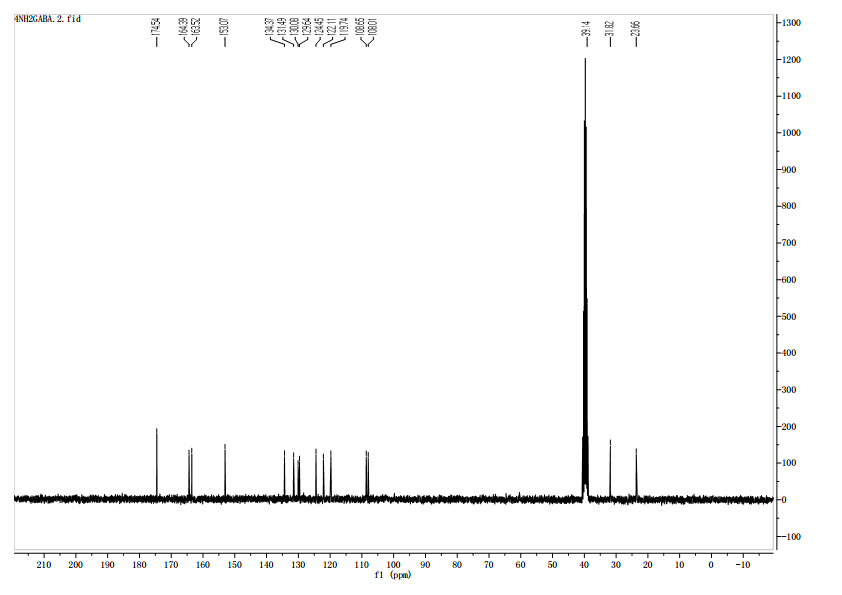


**Figure S36.** ^13^CNMR spectrum of compound **11b**.


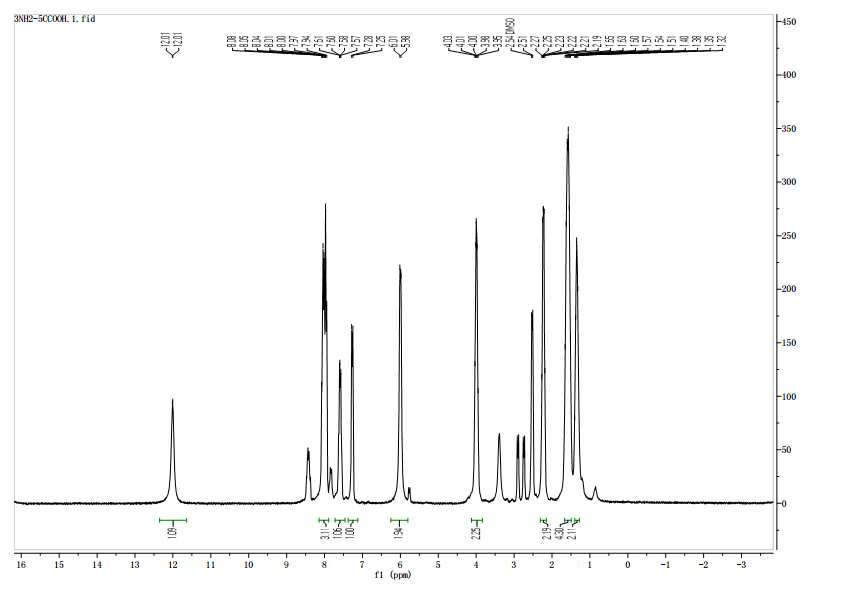


**Figure S37.** ^1^HNMR spectrum of compound **5c**.


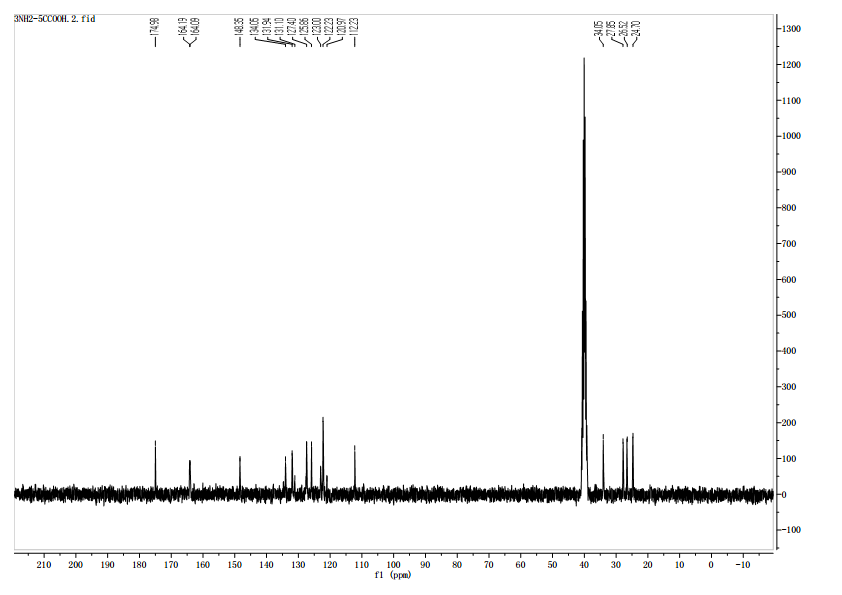


**Figure S38.** ^13^CNMR spectrum of compound **5c**.


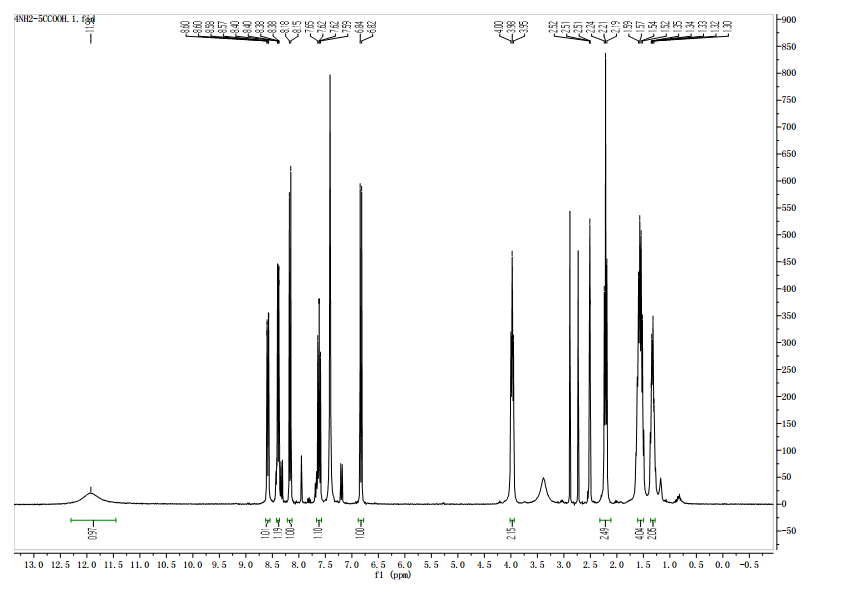


**Figure S39.** ^1^HNMR spectrum of compound **11c**.


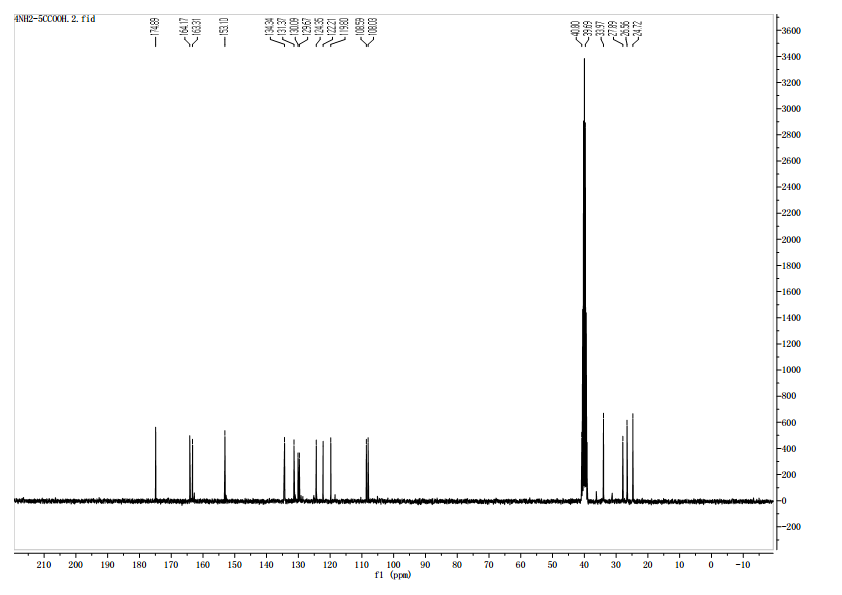


**Figure S40.** ^13^CNMR spectrum of compound **11c**.


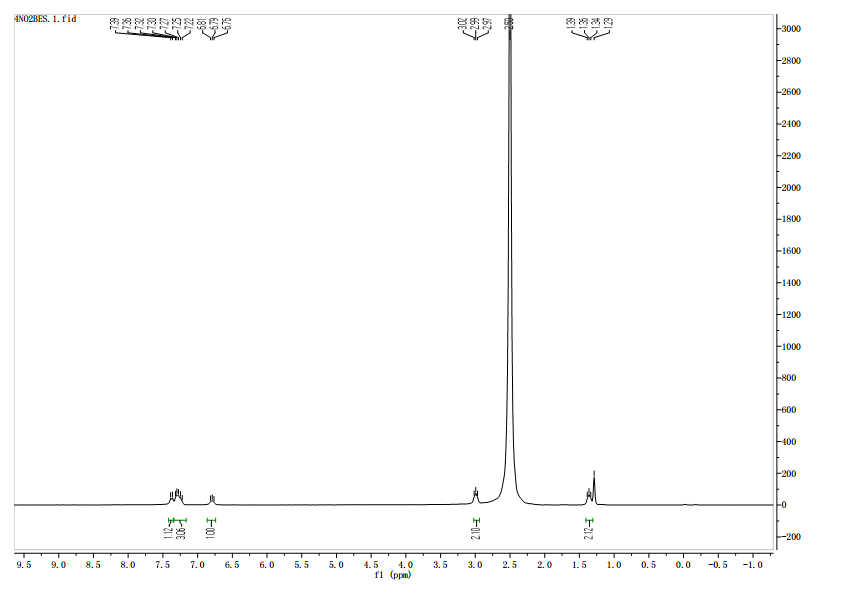


**Figure S41.** ^1^HNMR spectrum of compound **11d**.


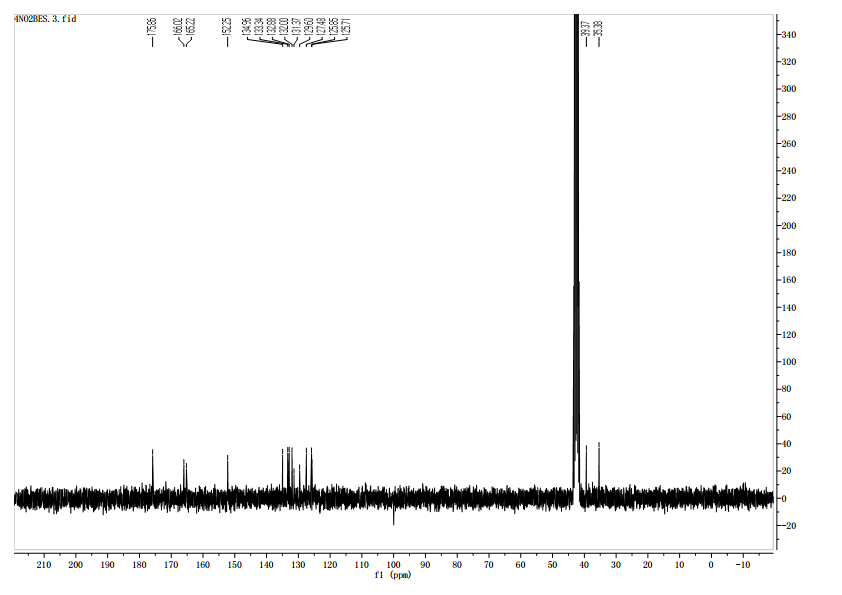


**Figure S42.** ^13^CNMR spectrum of compound **11d**.

**a. b.**

**Figure S43.** Stability of naphplatin in water and RPMI 1640 tested by RP-HPLC at room temperature in dark. **(a).** Stability of compounds naphplatin in water. (**b).** Stability of compounds naphplatin in RPMI-1640.

**Table S4.** IC_50_ values (concentrations of 50% inhibition of cell proliferation) (micromolar) of **7a-7c, 12a-12d** and **14a**, naphplatin, the positive control cisplatin and oxaliplatin. The cell viability was determined by MTT assays^[a]^.

|  |  |  | **Colorectal**  **Cancer** | | **Hepatic**  **Cancer** | | **Breast**  **Cancer** | | **Lung cancer** |
| --- | --- | --- | --- | --- | --- | --- | --- | --- | --- |
|  | m | R | HT-29 | HCT-116 | HepG-2 | Huh-7 | MDA-MB-231 | MCF-7 | A549 |
| **7a** | 0 | / | 2.56±0.30 | 2.59±0.25 | 7.38±0.71 | >30 | 1.41±0.11 | 18.65±1.45 | / |
| **7b** | 1 | / | 9.56± 0.61 | 8.48±0. 65 | 13.11±1.23 | >30 | 2.09±0.21 | 11.81±1.23 | 33.33±1.23 |
| **7c** | 3 | / | 7.89± 0.71 | 9.87±0.32 | 1.84±0.1.25 | >30 | 0.87±0.04 | 10.96±1.02 | / |
| **11a** | 0 | NH_2_ | >30 | >30 | >30 | >30 | >30 | >30 | >30 |
| **12a** | 0 | NH_2_ | 1.23 ± 0.15 | 0.95±0.09 | 2.19±0.22 | 1.33±0.123 | 2.98±0.32 | 8.48±0.81 | 24.4±2.36 |
| **12b** | 1 | NH_2_ | 2.59 ± 0.35 | 2.56±0.34 | 2.88±0.48 | 21.12±2.20 | >30 | 4.69±0.45 | 26.77±2.56 |
| **12c** | 3 | NH_2_ | 7.89± 0.74 | 8.23±0.81 | 8.62±0.85 | >30 | 2.05±0.20 | >30 | / |
| **12d** | 0 | NO_2_ | 10.26 ± 1.02 | 11.25±1.10 | 16.26±1.54 | 19.89±2.20 | >30 | 31.33±3.23 | / |
| **14a** | 0 | NO_2_ | 11.89± 1.15 | 12.56±0.92 | 9.63±0.65 | 11.88±1.10 | 7.16±0.61 | 25.35±2.45 | / |
| **Naph.** | 0 | NH_2_ | 0.09±0.03 | 0.06±0.04 | 0.45±0.06 | 1.34±0.12 | 0.65±0.05 | 2.21±0.22 | 8.63±0.48 |
| **13** | / | / | 16.23±1.25 | 18.56±2.02 | 15.48±2 | 16.23±1.56 | 26.36±2.36 | 19.48±1.89 | / |
| **Cis.** | / | / | 5.70 ± 0.02 | 6.78 ± 0.02 | 8.90±0.84 | 15.02±1.50 | 32.48±1.23 | 9.60±0.69 | 10.20±0.88 |
| **FI^[b]^** | / | / | 63.33 | 113.00 | 19.78 | 11.21 | 49.9 | 4.34 | 0.090 |
| **Oxp.** | / | / | 11.77±2.02 | 9.26± 0.42 | 19.98±1.25 | 16.17±1.60 | 17.72±0.92 | 11.62±0.81 | 10.00±1.00 |
| **FI^[c]^** | / | / | 130.78 | 154.33 | 44.40 | 12.07 | 27.26 | 5.26 | 0.94 |

[a] An average of three measurements. Naph. = Naphplatin. Cis. = Cisplatin. Oxp. = Oxaliplatin.

[b] FI (fold increase) is defined as IC_50_(cisplatin)/IC_50_(Naph.).

[c] FI (fold increase) is defined as IC_50_(oxaliplatin)/IC_50_(Naph.).

**Table S5.** IC_50_ values (concentrations of 50% inhibition of cell proliferation) (micromolar) of naphthalimide intermediates **5a-5c** and **11a-11d**, the positive control amonafide, cisplatin and oxaliplatin. The cell viability was determined by MTT assays^[a]^.

|  | HepG-2 | Huh-7 | MDA-MB-231 | MCF-7 | A549cisR | A549 | RF[b] |
| --- | --- | --- | --- | --- | --- | --- | --- |
| **5a** | >30 | >30 | >30 | >30 | >30 | >30 | >30 |
| **5b** | >30 | >30 | >30 | >30 | >30 | >30 | >30 |
| **5c** | >30 | 4.14±0.40 | >30 | >30 | >30 | >30 | >30 |
| **11a** | >30 | >30 | >30 | >30 | 11.5±1.08 | >30 | >30 |
| **11b** | >30 | >30 | >30 | >30 | >30 | >30 | >30 |
| **11c** | >30 | 3.08±0.40 | >30 | >30 | >30 | >30 | >30 |
| **11d** | 22.61±2.13 | >30 | >30 | >30 | 8.8±1.02 | >30 | >30 |
| Amonafide | 9.38±0.42 | 8.52±0.5 | 10.23±1.02 | 15.93 ±0.5 | 9.56±0.94 | 23.46 ±2 | 0.41 |
| Cisplatin | 8.90±0.88 | 15.02±1 | 32.48±1.23 | 9.60±0.65 | 42.89±4.23 | 10.20±0. 90 | 4.01 |
| Oxaliplatin | 19.98±2.00 | 16.17±2 | 17.72±0. 92 | 11.62±0.70 | 38.97±3.56 | 10.00±1.00 | 3.9 |

[a] An average of three measurements. ND = Not determined.

[b] The RF (resistance factor) is defined as the IC_50_ value in A549cisR cells/IC_50_ value in A549 cells.

**Table S6**. IC50 values (μM) of Pt(IV) prodrugs. Cells were treated for 48 h, and the cell viability was determined by MTT assays[b]

|  | Huh-7 | HL-7702 | SI^[a]^ |
| --- | --- | --- | --- |
| naphplatin | 1.34±0.10 | 6.47±0.52 | 4.83 |
| Cisplatin | 23.37±1.23 | 5.6±0.21 | 0.24 |
| Oxaliplatin | 11.77±2.00 | 6.77±0.82 | 0.58 |

[a] SI(selectivity index) is defined as IC50 in HL-7702/IC50 in Huh-7.

^[b]^ An average of three measurements.


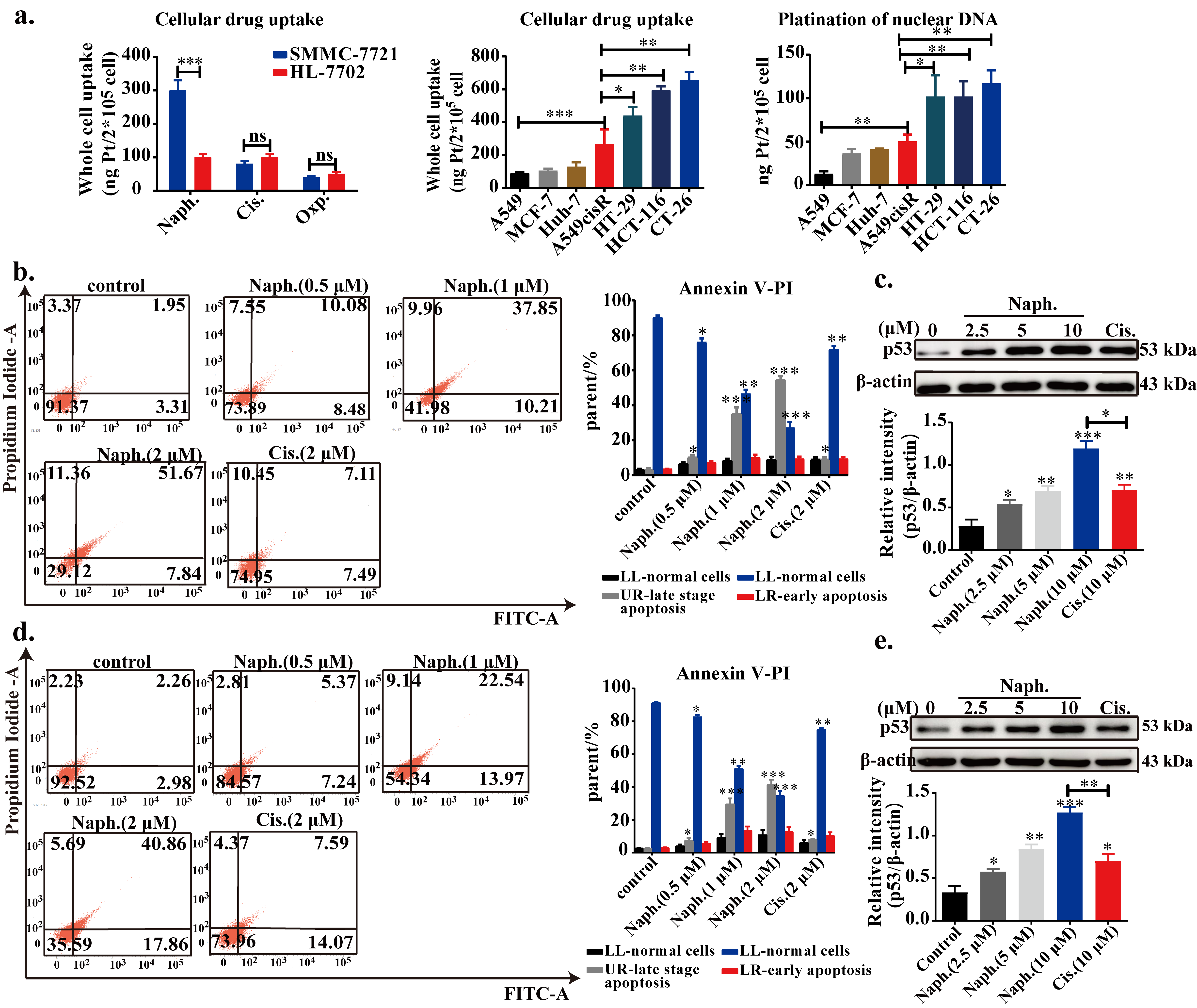


**Figure S44. *In vitro* biological evaluation by ICP-MS, flow cytometry and western blotting. (a, left)** Cellular uptake and distribution in cancer cells and matched normal cells with 10 μM of tested complexes. **(a, middle)** Cellular drug uptake and DNA platination **(a, right)** of naphplatin in lung carcinoma cell A549 and A549cisR, hepatoma carcinoma cell SMMC-7721, breast cancer cell MCF-7 and colorectal cancer HCT-116 by ICP-MS. (**b, d**) The percentage of apoptotic by flow cytometry in CT-26 (**b**) and HCT-116 (**d**) cells treated without or with naphplatin 0.5 μM, 1 μM, 2 μM and cisplatin 2 μM. **(c, e)** Effects of cisplatin and naphplatin on p53 expression in CT-26 (**c**) and HCT-116 (**e**) cells.*, P < 0. 05 **, P < 0.01 ***, P < 0.001.

**Table S7.** *In vivo* maximum tolerated dose and lethal dose values and calculated therapeutic indices (LD_50_/IC_50_) of naphplatin, cisplatin and oxaliplatin. The maximum tolerated dose (MTD) was evaluated by calculating body weight loss (mean weight loss < 15% and < 15% toxic deaths).

| Complexes | MTD  (mg/kg) | LD_50_ (mg/kg) | LD_50_ (µM/kg) | 48 h  Avg  IC_50_(µM) | LD_50_/  IC_50_ |
| --- | --- | --- | --- | --- | --- |
| Naphplatin | 40.00 | 78.00 | 93.17 | 0.030 | 3105.67 |
| Cisplatin | 8.00 | 15.00 | 49.99 | 6.78 | 7.37 |
| Oxaliplatin | 12.00 | 29.00 | 72.99 | 9.26 | 7.88 |

**
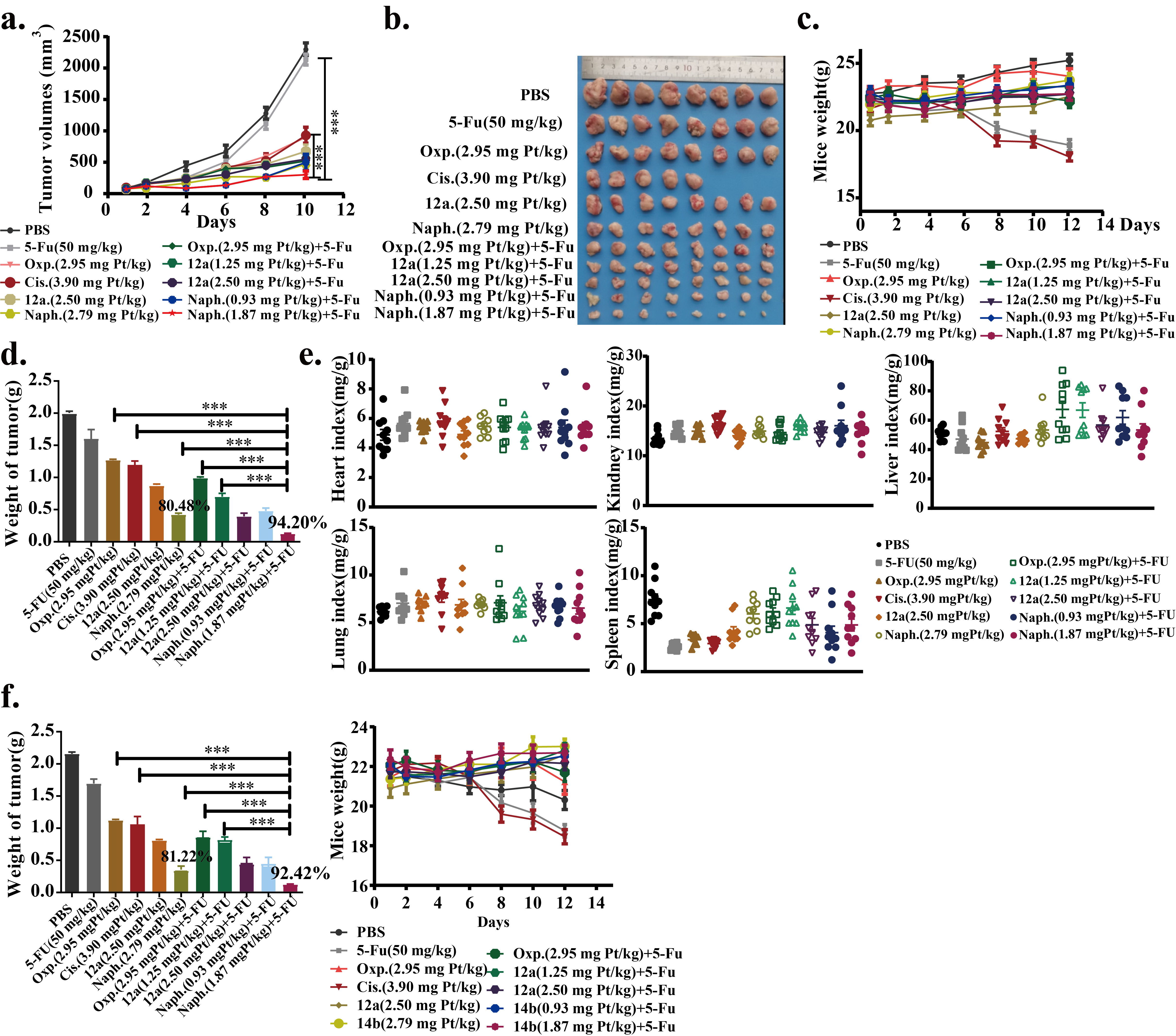
**

**Figure S45.** **The remarkably enhanced anti-tumour (94.20%) activities by naphplatin in colorectal carcinoma (CT-26 a-e and MC-38 f)*.* (a)** Average tumour growth curve with time during anti-tumour growth *in vivo*. **(b)** Images of the tumours at the end of the experiments. The drugs are injected by intravenous once every 2 days (n = 8 mice per group). **(c)** The body weight of the mice during anti-tumour growth *in vivo*. **(d)** The tumour weight in each group at the end of the experiments. **(e)** Organ weight indexes (a. heart, b. liver, c. spleen, d. lung, and e. kidney) after treatment with control group, 5-Fu (50 mg/kg), cisplatin (3.90 mg Pt/kg), oxaliplatin (2.95 mg Pt/kg), **12a**(2.50 mg Pt/kg), naphplatin (2.79 mg Pt/kg), oxaliplatin + 5-Fu, **12a**(1.25 mg Pt/kg) + 5-Fu, **12a**(2.50 mg Pt/kg) + 5-Fu, naphplatin (0.93 mg Pt/kg) + 5-Fu, naphplatin (1.87 mg Pt/kg) + 5-Fu for the anti-tumour activity *in vivo*. **(f)** The body weight of the mice and the tumour weight in each group at the end of the experiments during anti-tumour growth in vivo (MC-38). *, P < 0. 05 **, P < 0.01 ***, P < 0.001.

**
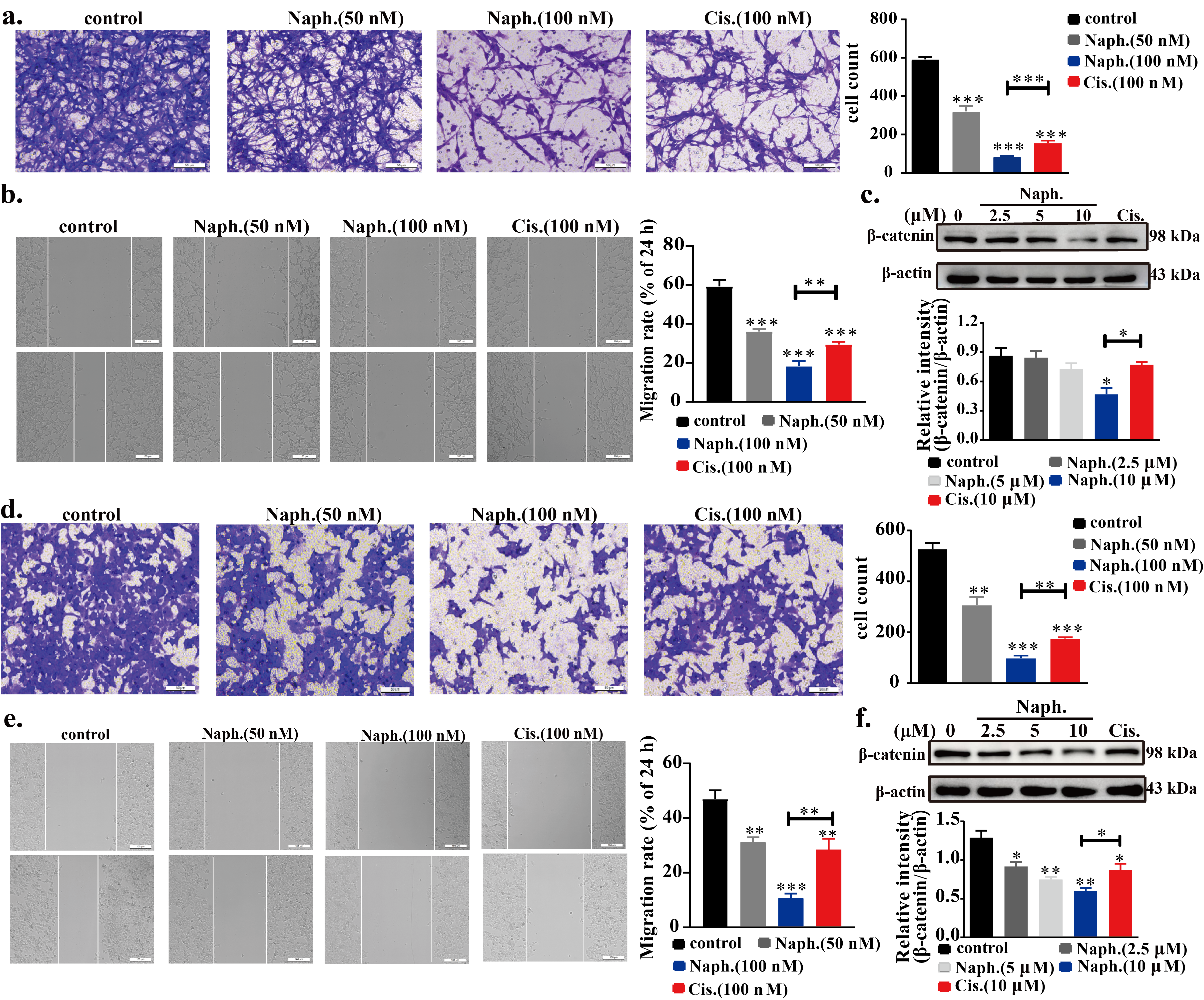
**

**Figure S46.** ***In vitro* anti-metastasis assays.** The invasion and migration of CT-26 (**a-c**) and HCT-116 (**d-f**) cells after treatment with naphplatin (2.5 µM, 5 µM, 10 µM) or cisplatin (10 µM) for 24 h by transwell **(a, d)**, wound healing **(b, e)** assays and the expression of β-catenin. *, P < 0. 05 **, P < 0.01 ***, P < 0.001.

**
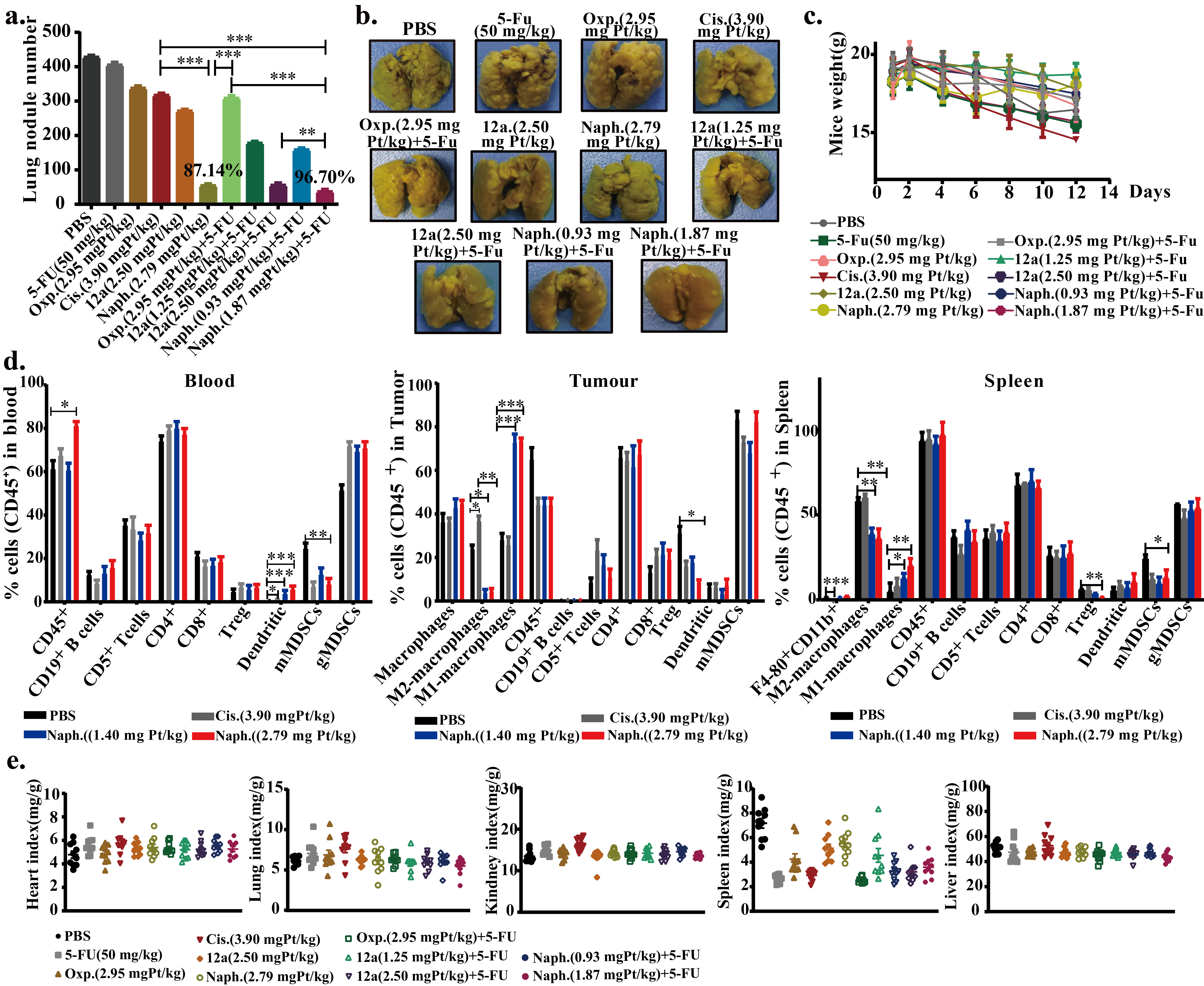
**

**Figure S47.** **The remarkably enhanced anti-metastasis (96.70%) activities and reinvigoration of tumour-associated macrophages by naphplatin in colorectal carcinoma*.*** **(a)** Statistics of lung metastasis nodules from mice after 14 days. **(b)** Representative images of pulmonary metastasis from mice at the end of the experiments. (**c**) Body weight of mice after various treatments for 14 days in the model of anti-tumour metastases *in vivo*. **(d)** Changes in the proportion of Macrophages, M2-macrophages, M1-macrophages, CD45^+^, CD19^+^B cells, CD5^+^Tcells, CD4^+^Tcells, CD8^+^Tcells, Treg, Dendritic, mMDSCs and gMDSCs in blood, tumour and spleen of the above anti-tumor model. **(e)** Organ weight indexes (heart, lung, kidney, spleen, and liver) after treatment with control group, 5-Fu (50 mg/kg), cisplatin (3.90 mg Pt/kg), oxaliplatin (2.95 mg Pt/kg), **12a**(2.50 mg Pt/kg), naphplatin (2.79 mg Pt/kg), oxaliplatin + 5-Fu, **12a**(1.25 mg Pt/kg) + 5-Fu, **12a**(2.50 mg Pt/kg) + 5-Fu, naphplatin (0.93 mg Pt/kg) + 5-Fu, naphplatin (1.87 mg Pt/kg) + 5-Fu for the anti-metastasis activity *in vivo*. *, P < 0. 05 **, P < 0.01 ***, P < 0.001.

**Table S8.** The life prolonging rate of BALB/c mice (n = 10) in the anti-tumour model.

|  | 5-Fu | Cis.  (3.90 mgPt/kg) | Oxp.  (2.95 mgPt/kg) | Oxp.  +5-Fu | Naph. (1.40 mg Pt/kg) | Naph. (2.79 mgPt/kg) | Naph. (0.93  mg Pt/kg)  +5-Fu | Naph. (1.87  mg Pt/kg)  +5-Fu |
| --- | --- | --- | --- | --- | --- | --- | --- | --- |
| Life prolonging rate | 0.37% | 19.17% | 45.11% | 67.29% | 62.78% | 82.71% | 98.49% | 136.84% |

**
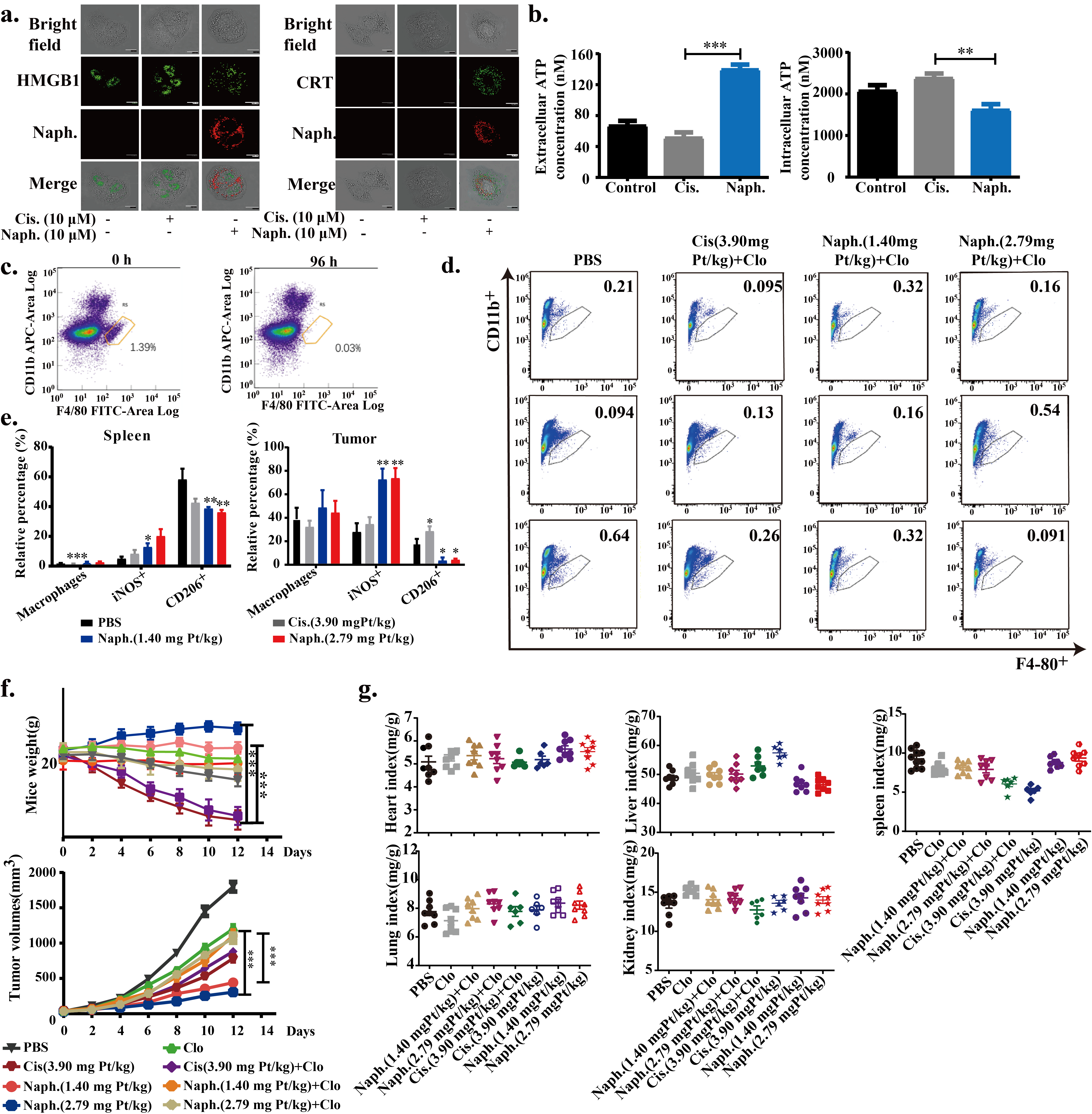
**

**Figure S48**. ***In vitro* and *in vivo* macrophage-deficient antitumor assay. (a)** HMGB1 release, CRT exposure, and **(b)** extracellular and intracellular ATP concentration of CT-26 colon cancer cells after different treatments. Scale bar represents 10 μm. (**c**) The proportion changes of macrophages and T cells by flow cytometry after intra-peritoneal injection of Clo (macrophage scavenger). (**d**) The clear effect of macrophages from the spleen by flow cytometry. (**e**) Changes in the proportion of macrophage in the spleen and tumour of sacrificed mice, including M2 macrophage markers CD206^+^ and M1 macrophage markers iNOS2. **(f)** Body weight of the mice and tumor growth during treatment in *in vivo* macrophage-deficient antitumor assay. (**g**) Organ weight indexes (heart, liver, spleen, lung, and kidney) after treatment with Clo, naphplatin, cisplatin, and normal saline for the naphplatin-reset macrophages to ameliorate tumor immuno-suppression *in vivo*. *, P < 0. 05 **, P < 0.01 ***, P < 0.001.

**
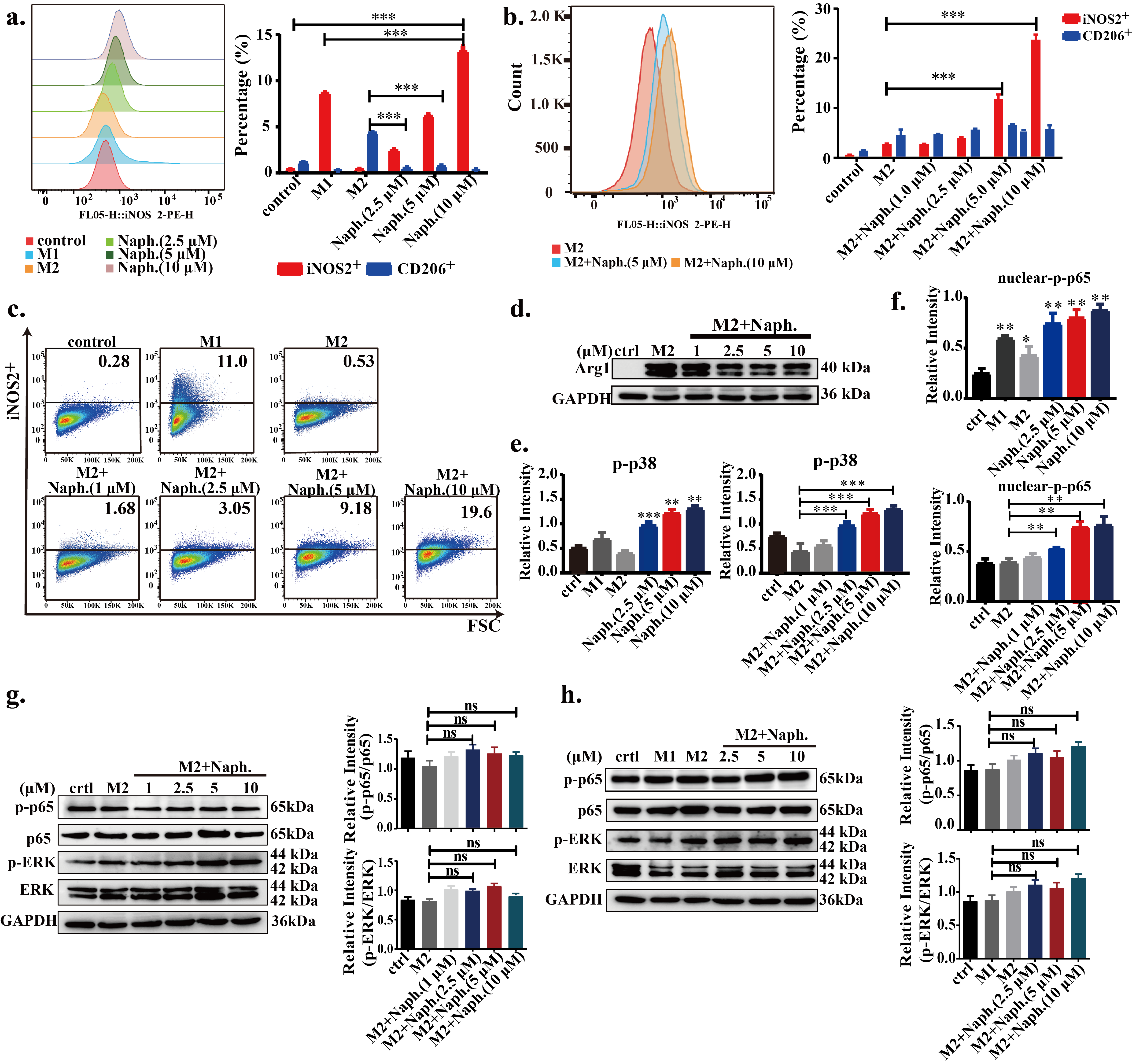
**

**Figure S49.** **Lysosome-mediated MAPK and NF-κB activation for M2 macrophage polarization.** **(a)** Representative flow cytometric analysis and quantification of iNOS and CD206 in BMDM isolated from mice. **(b)** Representative flow cytometric analysis and quantification of iNOS and CD206 in M2-BMDM. **(c)** Images of the expression of iNOS2 in M2-BMDM by naphplatin. **(d)** The protein expressions of Arg1 in the same F4/80+ ascites macrophages by western blotting. **(e-f)** The expressions of p-p38, total p38, nuclear p-p65, and total p65 in BMDM and M2-BMDM cells with or without naphplatin treatment. **(g-h)** The changes of cytoplasm p-p65, total p65, p-ERK and ERK by western blotting in BMDM and M2-BMDM stimulated with IL-4 and naphplatin (1 μM, 2.5 μM, 5 μM and 10 μM) for 24h . *, P < 0. 05 **, P < 0.01 ***, P < 0.001.


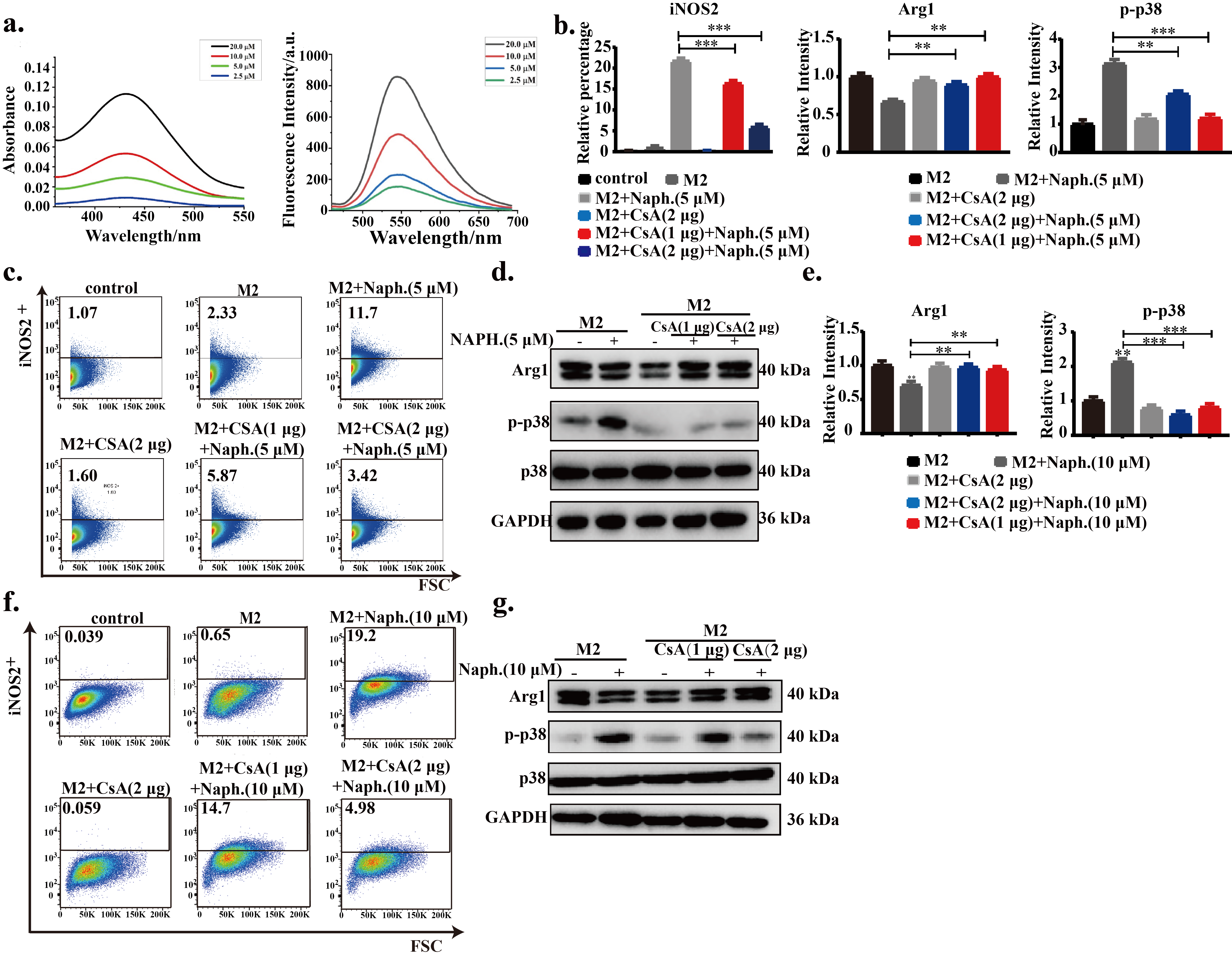


**Figure S50. The reversion of Cyclosporin A (CsA), a Ca^2+^ signaling inhibitor, on the effect of naphplatin-induced p38 phosphorylation and M2 macrophage polarization.** (**a**) The changes in the absorption and emission spectra of naphplatin in H_2_O and PBS. **(b-d)** The expression of iNOS , Arg1 and p-p38/p38 in BMDM-M2 cells pre-treated for 2 h with CsA (1 μg or 2μg) before addition of naphplatin (5 μM) or PBS (n = 3). **(e-g)** The expression of iNOS, Arg1 and p-p38/p38 in BMDM-M2 cells pre-treated for 2 h with CsA (1 μg or 2μg) or not before addition of naphplatin (10 μM) (n = 3). *, P < 0. 05 **, P < 0.01 ***, P < 0.001.


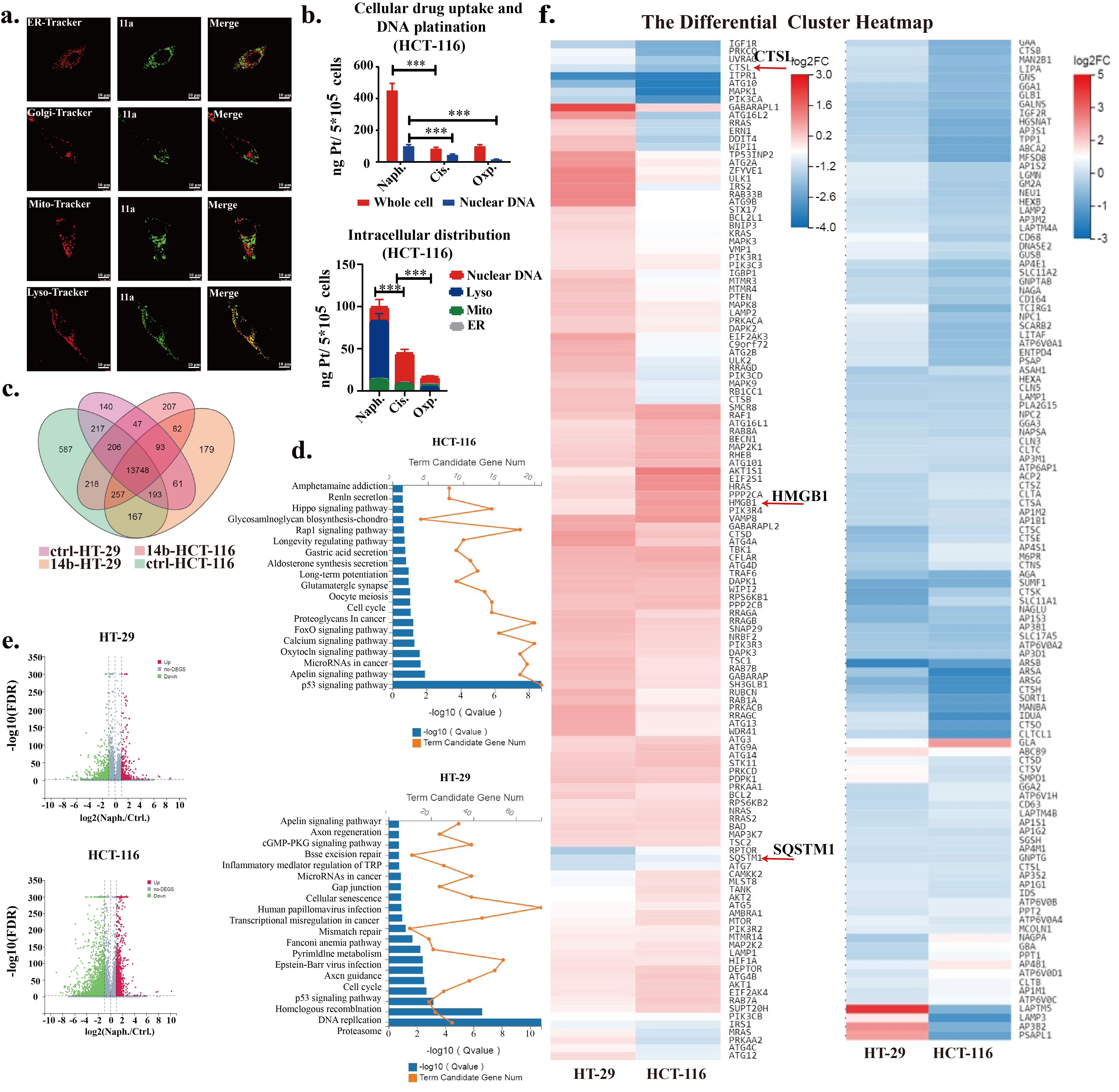


**Figure S51. The changes of the related genes after treatment with naphplatin in HCT-116 and HT-29 by RNA-Seq.** **(a)** Images of sub-organelle localization of the ligand **11a** in CT-26 cells by confocal microscopy. CT-26 cells were treated with **11a** for 2 h followed by staining with Mito-tracker, Golgi-tracker, ER-tracker, and Lyso-traker. **(b)** Cellular drug uptake, DNA platination and intracellular distribution of naphplatin, cisplatin and oxaliplatin in CT-26 by ICP-MS. **(c)** Venn diagram showing the number of overlapping DEGs in naphplatin-treated HCT-116 and HT-29 cells. **(d)** The top 20 pathways in both HCT-116 and HT-29 colon cancer cells. **(e)** Two MA plots, depicting the log-fold changes with signifcant DEGs marked in colour. Cells were treated with naphplatin (10 μM) for 24 h in both CT-26 and HCT-116 colon cancer cells. **(f, left)** The expression of genes related to lysosomal function and autophagy from RNA-seq analysis. **(f, right)** The expression of 113 genes related to lysosomal function from RNA-seq analysis.*, P < 0. 05 **, P < 0.01 ***, P < 0.001.


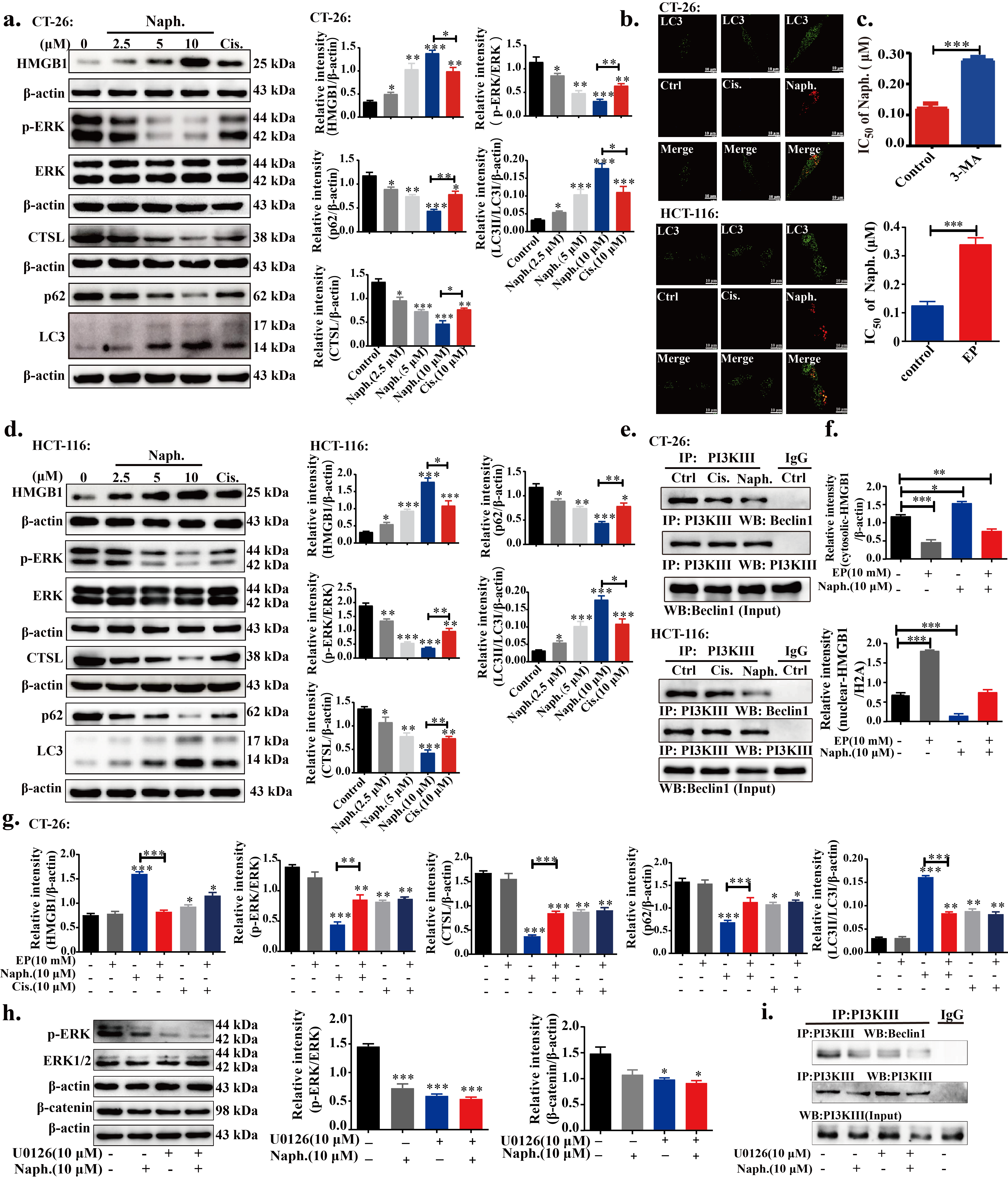


**Figure S52. HMGB1-mediated CTSL-lysosome function blockade in colorectal cancer. (a, d)** The expression of proteins related to HMGB1-mediated CTSL and autophagy-lysosome function *via* Beclin-1-PI3K-III/MEK/ERK signal pathway by western blotting and immunofluorescence in CT-26 cells (**a**) and HCT-116 cells (**d**). **(b)** The immunofluorescence of LC3I/II in both CT-26 (up) and HCT-116 (down) cells. **(c)** Cell viability by MTT assay with or without 3-MA and IC_50_ of naphplatin with or without EP. **(e)** Co-immunoprecipitation analysis of PI3KIII binding to Beclin1 in both CT-26 and HCT-116 cells with 10 µM naphplatin after 24 h treatment. **(f)** The statistics of whole cell lysates, nuclear extracts and cytoplasmic fractions for HMGB1by western blot. CT-26 cells were pre-treated with or without ethyl pyruvate (EP, 10 mM, 1 h) before addition of naphplatin (10 μM) for 24 h. **(g)** The statistics of HMGB1, p-ERK, ERK1/2, CTSL, p62 and LC3II/LC3I treated with 10 μM naphplatin by western blot analysis in the presence or absence of EP (10 mM, 2 h) in CT-26 cells. **(h)** The expression of p-ERK, ERK1/2, and β-catenin in the presence or absence of the MEK/ERK1/2 pathway U0126 in CT-26 cells **(i)** Co-immunoprecipitation analysis of PI3KIII binding to Beclin1 in CT-26 cells with 10 µM naphplatin after 24 h treatment. Each point or bar represents mean ± S.D. of triplicate determinations. *, P < 0. 05 **, P < 0.01 ***, P < 0.001.


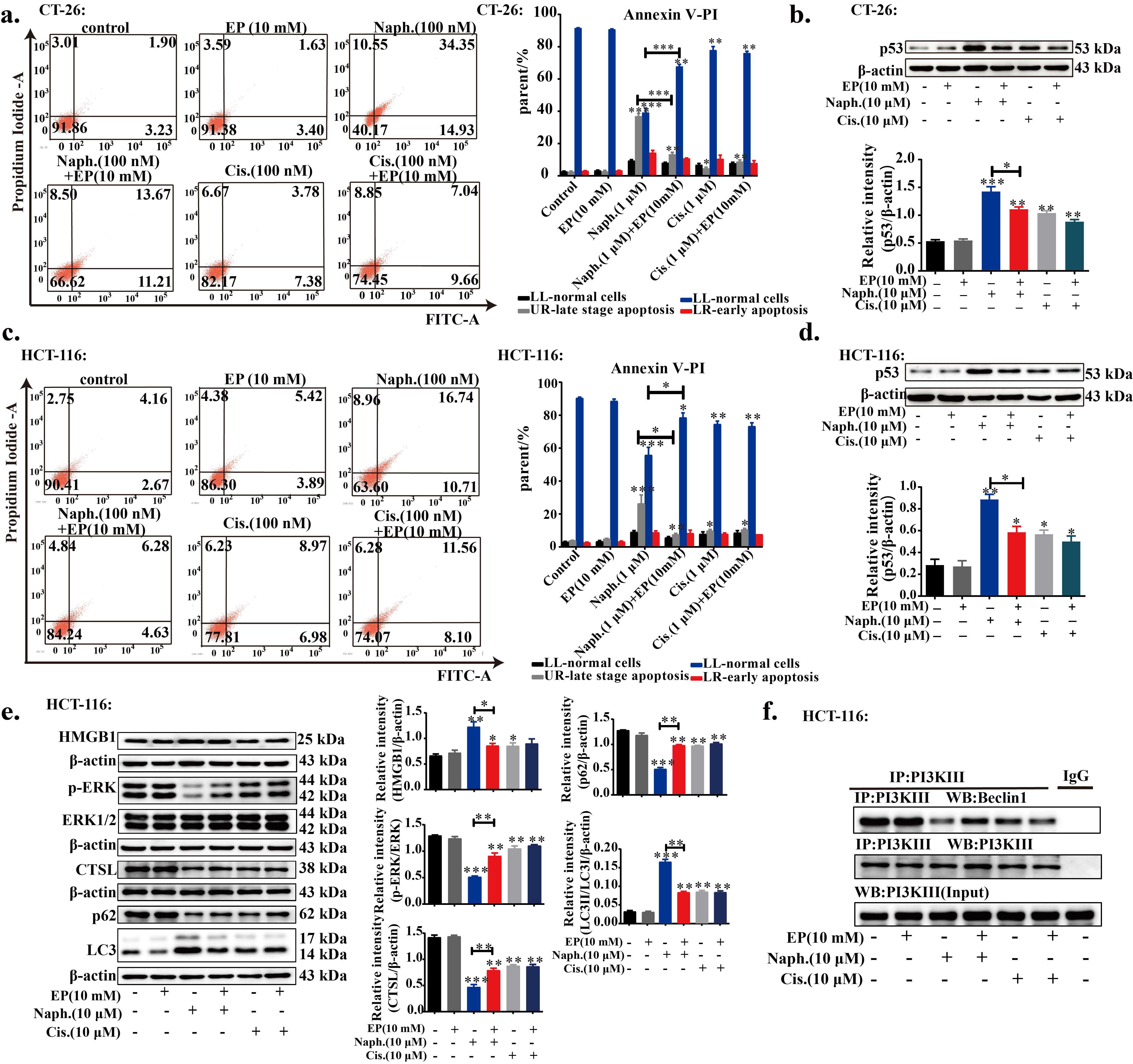


**Figure S53. The changes of HMGB1-mediated CTSL-lysosome signal pathway and apoptosis in the presence or absence of EP (10 mM, 2 h) in colorectal cancer.** (**a-d**) Apoptosis by flow cytometric analysis of Annexin-V/PI staining **(a, c)** and p53 expression with and without EP by western blot analysis **(b, d)** in CT-26 **(a-b)** and HCT-116 **(c-d)** colon cancer cells. **(e)** The expression of HMGB1, p-ERK, ERK1/2, CTSL, p62 and LC3II/LC3I in the presence or absence of EP (10 mM, 2 h) in HCT-116 cells. **(f)** Co-immunoprecipitation analysis of PI3KIII binding to Beclin1 in HCT-116 cells with 10 µM naphplatin after 24 h treatment. *, P < 0. 05 **, P < 0.01 ***, P < 0.001.


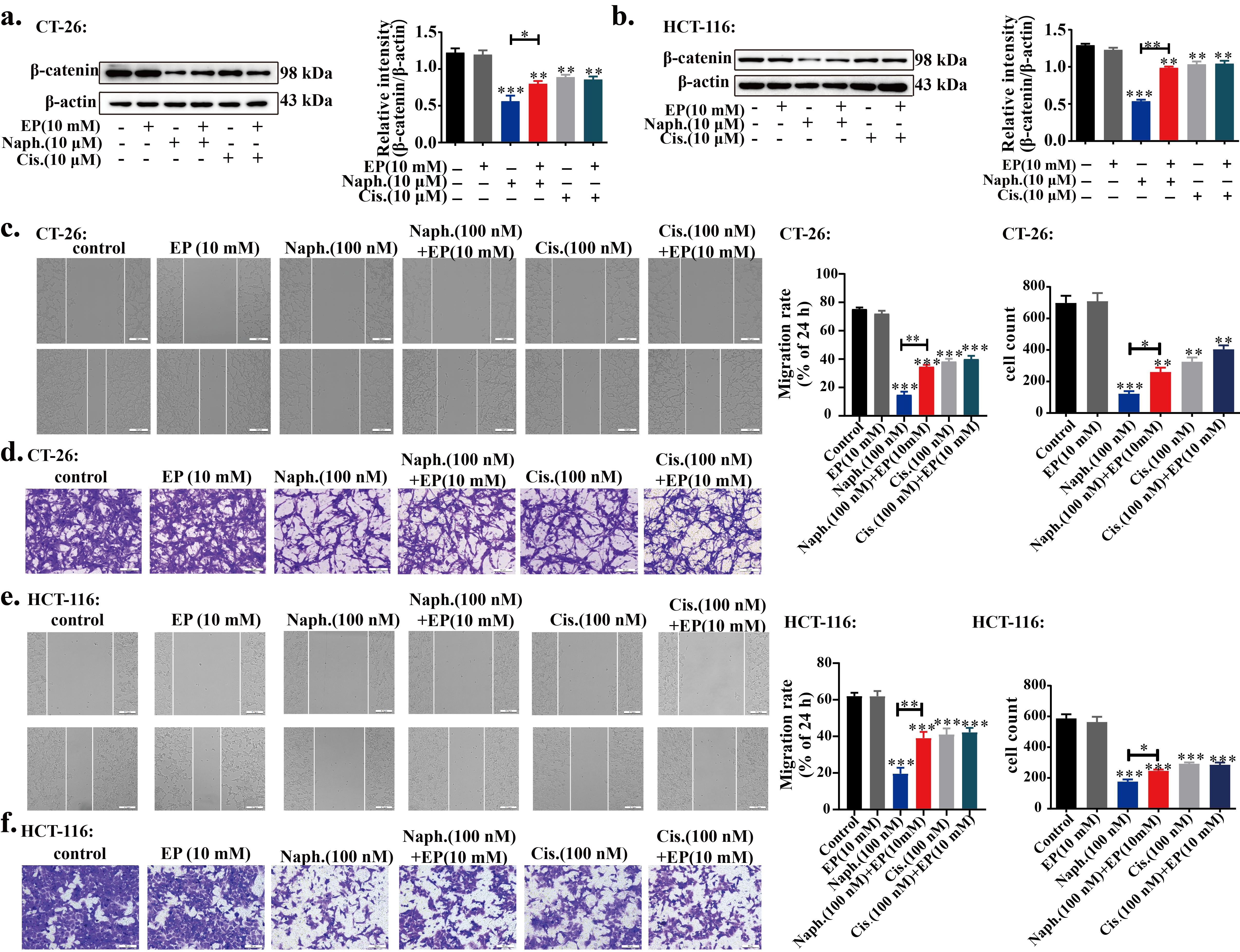


**Figure S54.** **The changes of invasion and migration in the presence or absence of EP (10 mM, 2 h) in colorectal cancer.** The expression of β-catenin **(a, b)** by western blot and invasion and migration by wound healing **(c, e)** and transwell assays **(d, f)** in CT-26 and HCT-116 colon cancer cells cells after treatment with naphplatin or cisplatin in the presence and absence of EP. *, P < 0. 05 **, P < 0.01 ***, P < 0.001.

REFERENCES

1. a) Chen Y, Heeg MJ, Braunschweiger PG, Xie W, Wang PG. A Carbohydrate-Linked Cisplatin Analogue Having Antitumor Activity. *Angew Chem Int Ed Engl.* **38**，1768-1769(1999). b) Ma J, Wang Q, Huang Z, Yang X, Nie Q, Hao W, Wang PG, Wang X. Glycosylated Platinum(IV) Complexes as Substrates for Glucose Transporters (GLUTs) and Organic Cation Transporters (OCTs) Exhibited Cancer Targeting and Human Serum Albumin Binding Properties for Drug Delivery. *J Med Chem.* **60**, 5736-5748(2017). c) Liu H, Ma J, Li Y, Yue K, Li L, Xi Z, Zhang X, Liu J, Feng K, Ma Q, Liu S, Guo S, Wang PG, Wang C, Xie S. Polyamine-Based Pt(IV) Prodrugs as Substrates for Polyamine Transporters Preferentially Accumulate in Cancer Metastases as DNA and Polyamine Metabolism Dual-Targeted Antimetastatic Agents. *J Med Chem.* ***62*,** 11324-11334(2019). d) Ma J, Li L, Yue K, Zhang Z, Su S, Chen Y, Yu L, Zhang P, Ma R, Li Y, Ma Y, Jia H, Wang C, Wang J, Xie S. A naphthalimide-polyamine conjugate preferentially accumulates in hepatic carcinoma metastases as a lysosome-targeted antimetastatic agent. *Eur J Med Chem.* **221**, 113469(2021). e) Ma J, Yang X, Hao W, Huang Z, Wang X, Wang PG. Mono-functionalized glycosylated platinum(IV) complexes possessed both pH and redox dual-responsive properties: Exhibited enhanced safety and preferentially accumulated in cancer cells in vitro and in vivo. *Eur J Med Chem.* **128**, 45-55(2017). f) Ma J, Wang Q, Yang X, Hao W, Huang Z, Zhang J, Wang X, Wang PG. Glycosylated platinum(iv) prodrugs demonstrated significant therapeutic efficacy in cancer cells and minimized side-effects. *Dalton Trans.* **45**, 11830-8(2016). g). Wang Q, Huang Z, Ma J, Lu X, Zhang L, Wang X, George Wang P. Design, synthesis and biological evaluation of a novel series of glycosylated platinum(iv) complexes as antitumor agents. *Dalton Trans.* **45**, 10366-74(2016). h). Ma J, Li L, Yue K, Li Y, Liu H, Wang PG, Wang C, Wang J, Luo W, Xie S. Bromocoumarinplatin, targeting simultaneously mitochondria and nuclei with p53 apoptosis pathway to overcome cisplatin resistance. *Bioorg Chem.* **99**, 103768(2020). i) Li Y, Yue K, Li L, Niu J, Liu H, Ma J, Xie S. A Pt(IV)-based mononitro-naphthalimide conjugate with minimized side-effects targeting DNA damage response via a dual-DNA-damage approach to overcome cisplatin resistance. *Bioorg Chem.* **101**, 104011(2020).
2. a) Wang, Y. X.; Zhang, X.; Zhao, J.; Xie, S. Q.; Wang, C. J. *J. Med. Chem.* **2012**, *55*, 3502−3512. Wang Y, Zhang X, Zhao J, Xie S, Wang C. Nonhematotoxic naphthalene diimide modified by polyamine: synthesis and biological evaluation. *J Med Chem.* **55**, 3502-12(2012). b) Dai F, Li Q, Wang Y, Ge C, Feng C, Xie S, He H, Xu X, Wang C. Design, Synthesis, and Biological Evaluation of Mitochondria-Targeted Flavone-Naphthalimide-Polyamine Conjugates with Antimetastatic Activity. *J Med Chem.* **60**, 2071-2083(2017). c). Li J, Tian R, Ge C, Chen Y, Liu X, Wang Y, Yang Y, Luo W, Dai F, Wang S, Chen S, Xie S, Wang C. Discovery of the Polyamine Conjugate with Benzo[ cd]indol-2(1 H)-one as a Lysosome-Targeted Antimetastatic Agent. *J Med Chem.* **61**, 6814-6829(2018). d). Li Q, Zhai Y, Luo W, Zhu Z, Zhang X, Xie S, Hong C, Wang Y, Su Y, Zhao J, Wang C. Synthesis and biological properties of polyamine modified flavonoids as hepatocellular carcinoma inhibitors. *Eur J Med Chem.* **121**, 110-119(2016).
